# Development and initial psychometric evaluation of a self-reported sustainable sport talent development literacy scale for physical education teachers

**DOI:** 10.3389/fpsyg.2026.1902630

**Published:** 2026-08-13

**Authors:** Xiaodan Jiang, Rui Xue

**Affiliations:** 1 College of P.E. and Sport, Beijing Normal University, Beijing, China; 2 School of Education, Beijing Sport University, Beijing, China

**Keywords:** measurement invariance, physical education teachers, psychometrics, scale development, sustainable sport talent development, teacher literacy, youth sport

## Abstract

**Background:**

School sport aims to identify and develop students’ sport potential while maintaining health, motivation, educational engagement, and equitable participation. However, few instruments capture how physical education (PE) teachers report the judgments and practice orientations through which they support sport potential under these developmental conditions.

**Objective:**

This study developed and conducted an initial psychometric evaluation of a self-report scale designed to assess PE teachers’ self-reported sustainable sport talent development literacy, operationalized as reported judgments and practice orientations.

**Methods:**

The scale was developed through a literature review, expert content review, pilot testing, and a formal online survey. Of 858 submitted questionnaires, 800 were retained after data-quality screening and randomly allocated to exploratory factor analysis (*n* = 400) and confirmatory factor analysis (*n* = 400) subsamples.

**Results:**

Exploratory factor analysis supported a six-factor structure comprising health protection, motivational support, education–training balance, potential cultivation, character guidance, and collaborative support. The correlated six-factor model also showed good fit in the confirmatory analysis (robust comparative fit index (CFI) = 0.985, robust Tucker-Lewis index (TLI) = 0.984, and robust root mean square error of approximation (RMSEA) = 0.018). Cronbach’s alpha coefficients ranged from 0.790 to 0.822 across the six dimensions, and composite reliability estimates were acceptable. Average variance extracted values ranged from 0.395 to 0.459, indicating limited evidence of convergent validity. The six dimension scores correlated between 0.465 and 0.537 with a separate, five-item study-developed sustainable PE practice-intention measure; the exploratory correlation with the unit-weighted total score was 0.727. Because the two measures were completed by the same respondents in the same survey and covered closely related self-reported orientations, these associations were interpreted as preliminary evidence of theoretical consistency rather than evidence of concurrent, criterion-related, or predictive validity. Observed-score comparisons showed little differentiation between teachers with and without experience guiding sport-talented or high-performing students. Because measurement invariance was not tested for this grouping variable, the results were not interpreted as indicating a latent group difference.

**Conclusion:**

The findings provide preliminary psychometric evidence for a six-dimensional measure of PE teachers’ self-reported sustainable sport talent development literacy. Scores reflect teachers’ reported judgments and practice orientations; they do not directly indicate demonstrated knowledge, judgment accuracy, professional competence, or observed teaching behavior. The scale may support exploratory research and teacher development, but validation against independent external criteria and behavioral assessments remains necessary.

## Introduction

School sport is expected to identify and cultivate students’ sport potential while protecting health, supporting inclusion, sustaining participation, and contributing to students’ wider development. These expectations are consistent with the United Nations’ sustainable development agenda, which links education with health, wellbeing, and equitable opportunities (United Nations, 2015), and with UNESCO’s view of quality physical education (PE) as a setting for lifelong participation, safety, equal access, and whole-person development (UNESCO, 2015). In this study, “sustainable” refers not only to policy-level sustainability but also to the developmental condition in which sport potential is cultivated without undermining students’ long-term participation, health and wellbeing, motivation, educational development, or equitable access to opportunities. Sport talent development in schools, therefore, cannot be reduced to early selection or performance acceleration. Its educational value depends on whether students’ potential is cultivated within conditions that also protect their long-term physical, psychological, social, and academic development.

Talent development environment studies link young athletes’ progress with a long-term developmental focus, broad preparation, communication, support networks, and aligned expectations among stakeholders (Martindale et al., 2010; Li et al., 2015; Andronikos et al., 2017). However, early specialization and intensive youth sport participation have been associated with injury risk, attrition, exhaustion, and psychological pressure (DiSanti and Erickson, 2019). Evidence on elite athletes’ mental health further indicates that intensive performance pathways may involve burnout and other psychological risks (Rice et al., 2016). Research on training load in adolescent athletes further emphasizes the need to monitor internal and external load in relation to physical qualities, injury, and illness (Dudley et al., 2023). Schools, therefore, need to recognize students’ sport potential without weakening health, motivation, learning, or fair participation.

PE teachers often observe students before formal talent pathways begin, across lessons, school teams, extracurricular activities, and class-based physical activity. This pre-pathway position gives PE teachers a distinctive influence that is not fully captured by instruments designed for athletes’ perceptions of talent environments or by broad measures of PE teacher professionalism. Their judgments influence how students experience competence, effort, comparison, safety, belonging, and access. Research in school PE has shown that autonomy, competence, and relatedness support are closely associated with students’ autonomous motivation and adaptive outcomes (Vasconcellos et al., 2020). Sustainable sport talent development in ordinary school settings, therefore, depends partly on how teachers identify potential, regulate participation demands, protect wellbeing, sustain motivation, and coordinate support.

Students differ in physical condition, movement experience, sex, family resources, motivation, and academic demands. A narrow focus on current performance may overlook late-developing students, expose students to inappropriate training expectations, or reinforce restricted pathways. PE teachers’ role in sustainable sport talent development is, therefore, not simply one of general PE professionalism. It requires teachers to weigh performance, health, motivation, education, character development, and equity together when making judgments about students’ sport potential.

The Talent Development Environment Questionnaire (TDEQ) assesses athletes’ perceptions of talent development environments (Andronikos et al., 2017). PE teacher literacy instruments address broader professional qualities or core literacy (Zeng et al., 2022; Li et al., 2026). Other measures focus on specific teaching domains, such as self-efficacy or competence in adapting PE activities (Reina et al., 2019; Xue et al., 2023). What remains undermeasured is how PE teachers report the judgments and practice orientations they bring to supporting students’ sport potential in developmental, school-based, and sustainable ways. Assessing these self-reports may help researchers and teacher educators identify areas in which teachers perceive a need for further support. Such scores do not, however, establish what teachers objectively know or can demonstrate in practice.

This study developed the Self-Reported Sustainable Sport Talent Development Literacy Scale for Physical Education Teachers and examined its initial psychometric properties across six theoretically derived dimensions. The focal construct was self-reported literacy, operationalized as teachers’ reports of their judgments and practice orientations. The evaluation addressed item development, internal structure, score reliability, discriminant evidence, measurement invariance, and the plausibility of a general-score interpretation. Associations with a separate practice-intention measure and the observed-score group comparisons were treated as exploratory because the study did not include an independent external criterion.

### Conceptual framework and item generation

In this study, self-reported sustainable sport talent development literacy is defined as the judgments and practice orientations that PE teachers report using when identifying, guiding, protecting, and supporting students’ sport potential. These reports concern developmental conditions intended to sustain participation, health and wellbeing, education–training balance, personal and social development, and equitable opportunities. The construct was assessed through first-person Likert items. Dimension scores, therefore, indicate the extent to which respondents endorse literacy-related judgments and practice orientations. They do not directly measure factual knowledge, the accuracy of professional judgment, demonstrated competence, or observed teaching behavior.

The term literacy was retained because PE teacher research uses it to describe an integrated domain of understanding, values, judgment, and context-sensitive practice rather than factual knowledge alone (Zeng et al., 2022; Li et al., 2026). The modifier self-reported specifies the level of inference supported by the present instrument. It distinguishes the reported orientations assessed here from competence demonstrated through knowledge tests, performance tasks, classroom observation, or independent ratings.

Sport talent in school settings is treated as developmental rather than fixed. It includes emerging sport potential, motor competence, motivation, persistence, learning responsiveness, and growth potential. The construct, therefore, concerns how PE teachers recognize and support developing potential rather than how they select already high-performing students.

The six domains were organized around the conditions that make school sport talent development sustainable. One condition is protection from avoidable developmental harm. Health protection items were grounded in research on early specialization, training load, injury, fatigue, recovery, psychological pressure, and burnout risk (DiSanti and Erickson, 2019; Dudley et al., 2023; Rice et al., 2016). This domain also reflects talent development environment research that emphasizes long-term development and broad preparation rather than narrow early concentration (Martindale et al., 2010; Li et al., 2015; Andronikos et al., 2017).

Sustained motivation is equally central to the construct. Self-determination theory proposes that autonomy, competence, and relatedness support help maintain adaptive motivation. Evidence from organized physical activity indicates that SDT-based instruction can improve intrinsic motivation and identified regulation (Manninen et al., 2022). In school PE, this implies teacher practices such as explaining the purpose of practice, supporting meaningful goals, giving competence-supportive feedback, reducing controlling comparison, and creating an accepting environment.

Education–training balance was included because school-based sport talent development occurs within students’ wider educational lives. Talent development environment research emphasizes long-term focus, broad preparation, communication, and alignment of expectations among stakeholders (Martindale et al., 2010; Li et al., 2015; Andronikos et al., 2017). In school contexts, these ideas concern teachers’ ability to coordinate sport participation with academic learning, rest, recovery, and broader student development.

Potential cultivation was included because identifying talent and developing talent are not the same task. Developmental models of youth sport emphasize that sport potential emerges through interactions among individual characteristics, learning opportunities, motivation, and supportive environments (Côté and Vierimaa, 2014; Varghese et al., 2022). PE teachers, therefore, need to observe current skill, effort, interests, persistence, and learning responsiveness, and then adapt tasks or provide developmental suggestions. This domain also guards against premature concentration on a single sport or narrow selection based only on current performance.

Character guidance reflects the personal and social purposes of school sport. Positive youth development through sport emphasizes that adult support, peer relationships, parental involvement, and life-skill learning can contribute to positive personal, social, and physical outcomes (Holt et al., 2017). In school PE, this domain covers rule awareness, responsibility, cooperation, resilience, respect, and the transfer of sport learning to broader school life.

Collaborative support reflects the ecological nature of sustainable sport talent development. Talent development environment research emphasizes communication, support networks, and stakeholder alignment (Martindale et al., 2010; Li et al., 2015; Andronikos et al., 2017). In school PE, this domain concerns teachers’ coordination with parents, class teachers, coaches, and school administrators, as well as their efforts to adapt activities and protect fair access to development opportunities.

Together, the six domains define the content of the self-report instrument. They cover teachers’ reported orientations toward protecting developmental conditions, supporting motivation, coordinating sport participation with education, cultivating potential, guiding character development, and mobilizing school and family support.

## Methods

### Scale development procedure

Scale development began with a review of the literature on talent development environments, youth sport specialization, self-determination theory, positive youth development through sport, and school PE. The review was used to define the construct and specify six content domains. The construct was positioned as school-based and teacher-centered, with a focus on teachers’ self-reported judgments and practice orientations concerning the identification and support of emerging sport potential in ordinary educational contexts.

An initial item pool was then generated following recommended procedures for construct measurement and scale development (MacKenzie et al., 2011; Boateng et al., 2018; Stefana et al., 2025). The construct definition was translated into six content domains, and behavioral indicators were extracted for each domain. Health protection items were informed by research on early specialization, training load, injury, fatigue, recovery, psychological pressure, and burnout risk (DiSanti and Erickson, 2019; Rice et al., 2016; Dudley et al., 2023). Motivational support items were based on self-determination theory and school PE research on autonomy, competence, and relatedness support (Manninen et al., 2022; Vasconcellos et al., 2020). Education–training balance and potential cultivation items drew on talent development environment research and developmental models of youth sport (Martindale et al., 2010; Li et al., 2015; Andronikos et al., 2017; Côté and Vierimaa, 2014; Varghese et al., 2022). Character guidance items were developed from positive youth development through sport, especially life skills, responsibility, peer relations, and transfer of learning (Holt et al., 2017). Collaborative support items were informed by talent development environment research on communication, support networks, and stakeholder alignment (Martindale et al., 2010; Andronikos et al., 2017).

Items were written as first-person statements about teachers’ judgments and reported practices in school PE, school teams, or extracurricular sport. Each item addressed one idea, described a judgment or practice within the teacher’s influence, and avoided claims about demonstrated competence or student outcomes. Existing instruments were used as conceptual references rather than as item sources. The TDEQ informed the environmental logic of long-term development, communication, and support, but the items were rewritten from the teacher perspective to elicit teachers’ self-reports rather than athletes’ perceptions of the environment. PE teacher literacy scales informed the broader professional framing, while the present item pool focused specifically on sustainable sport talent development. Because the instrument relies on self-report, item endorsement represents a stated judgment or practice orientation rather than direct evidence of professional competence.

Five experts were purposively selected because each had sustained research or professional experience in an area directly relevant to the construct and item review. The panel comprised a specialist in PE curriculum and pedagogy with experience in school curriculum standards, instructional design, and teacher behavior; a specialist in educational and psychological measurement with expertise in scale development and item construction; a specialist in youth sport training and talent development; a specialist in sport psychology and positive youth development; and a specialist in inclusive education and education for sustainable development. This composition provided complementary perspectives on the school PE context, psychometric item quality, youth talent development, motivational and psychosocial development, and educational inclusion. The experts completed their ratings independently.

Each expert rated every item for relevance, clarity, and necessity on a four-point scale, with higher scores indicating a more favorable assessment. Ratings of 3 or 4 were treated as endorsement of the item. For each criterion, the item-level content-validity index (I-CVI) was calculated as the proportion of experts assigning a rating of 3 or 4. With five experts, an I-CVI of 0.80 or higher, corresponding to endorsement by at least four experts, was considered acceptable. An S-CVI/Ave of 0.90 or higher was used as the scale-level criterion. Relevance, clarity, and necessity were quantified separately. I-CVI values ranged from 0.80 to 1.00 for each criterion. The S-CVI/Ave values were 0.981 for relevance, 0.986 for clarity, and 0.990 for necessity; the corresponding S-CVI/UA values were 0.905, 0.929, and 0.952.

Four items had a relevance I-CVI of 0.80 (HWB7, PEB5, PCS7, and CIS7), three had a clarity I-CVI of 0.80 (AMS2, AMS5, and CIS5), and two had a necessity I-CVI of 0.80 (PID4 and PCS7). Because PCS7 was identified under both relevance and necessity, eight unique items did not receive unanimous endorsement on at least one criterion. The remaining 34 items received I-CVI values of 1.00 for all three criteria.

The quantitative thresholds were used to identify items for closer review rather than as automatic deletion rules. The ratings were considered together with the experts’ written comments. AMS2 and AMS5 were retained after wording revisions to improve clarity. PID4 was retained after abstract terminology was replaced with a more concrete judgment, and CIS5 was retained after the item was focused more clearly on teacher intervention. HWB7, PEB5, PCS7, and CIS7 were not retained in their original wording because of concerns about conceptual fit, cross-domain overlap, behavioral framing, or the extent of teacher control. The review also prompted broader content balancing to strengthen the representation of relatedness support, avoidance of early specialization, and equitable participation. Overall, the process produced a net reduction from 42 to 36 items, with six items assigned to each domain. No item was removed solely because its I-CVI was 0.80.

A pilot survey involving 40 PE teachers was then conducted to examine missing responses, response distributions, corrected item–total correlations, and preliminary reliability. Wording was refined for several items, particularly within the health protection domain. No items were added or removed at this stage. The formal survey, therefore, comprised the fixed 36-item core scale, with six items assigned to each of the six prespecified domains, together with five items assessing sustainable PE practice intention. The item set, domain assignments, and domain labels were finalized before the formal survey data were analyzed.

## Participants and procedure

The formal survey used convenience sampling. Data were collected through Wenjuanxing, a widely used online survey platform in China, between February and March 2026. Participants were recruited through PE teacher professional networks and school contacts in Beijing, Fujian, Shandong, Sichuan, Shanxi, and Liaoning. Recruitment was non-probabilistic, and the sample was not intended to represent the national population of PE teachers. Teachers who were more engaged in professional learning or interested in sport talent development may have been more likely to participate.

The survey yielded 858 submitted questionnaires. Data-quality screening was completed before examining item-level statistics, reliability estimates, or factor-analytic results. The screening rules were applied sequentially so that each excluded questionnaire was assigned one primary reason. Duplicate submissions were identified using a pseudonymous device/browser token recorded by the survey platform. When the same token appeared more than once, the earliest complete submission was retained, and each subsequent submission was excluded. This rule resulted in the exclusion of 16 questionnaires. Questionnaires completed in less than 180 s were then excluded. This cutoff was established before the psychometric analyses based on the 40-participant pilot study, in which the shortest completion time was 186 s. A threshold of 180 s was, therefore, adopted as a conservative rounded lower bound. Twenty-seven questionnaires fell below this threshold. Straight-lining was defined as selecting the same response category for all 36 core scale items, equivalent to zero within-person response variance. Fifteen questionnaires met this criterion. Together, the three mutually exclusive screening rules resulted in the exclusion of 58 questionnaires. No separate exclusion rule was applied to “extreme or implausible” response records. In particular, no questionnaire was excluded because of an unusually high or low scale score, statistical rarity, or divergence from the majority response pattern. After data-quality screening, 800 questionnaires were retained, corresponding to a retention rate of 93.2%.

Eligible participants were in-service PE teachers working in primary, junior high, senior high, 9-year, complete secondary, or other school settings. The first page of the online questionnaire described the academic purpose of the study, the voluntary nature of participation, anonymous handling of responses, and the right to withdraw before submission. Participants indicated electronic informed consent before accessing the questionnaire. No personally identifying information was collected. The retained questionnaires contained no missing responses because the online form required completion of each item before submission. The retained dataset contained no out-of-range responses, platform-detected duplicate records, or straight-line response patterns across all 36 core items. The questionnaire included demographic items, the 36-item Self-Reported Sustainable Sport Talent Development Literacy Scale for Physical Education Teachers, and five items measuring sustainable PE practice intention.

The retained sample was divided into non-overlapping exploratory factor analysis (EFA) and confirmatory factor analysis (CFA) subsamples using a reproducible randomization procedure. After the random seed was set to 20,260,531 in R, the 800 records were randomly permuted. The first 400 permuted records were assigned to the EFA subsample, and the remaining 400 were assigned to the CFA subsample. The primary CFA was conducted using only the designated CFA subsample.

Table 1 reports the core demographic characteristics, school location, and variables used in subsequent group-based analyses.

**Table 1:** Participant characteristics.

Characteristic	Category	*n*	%
Sex	Male	477	59.6
	Female	310	38.8
	Other/prefer not to say	13	1.6
Age	25 years or below	71	8.9
	26–35 years	292	36.5
	36–45 years	301	37.6
	46 years or above	136	17.0
Teaching experience	5 years or below	192	24.0
	6–10 years	192	24.0
	11–20 years	292	36.5
	21 years or above	124	15.5
Education	Junior college or below	59	7.4
	Bachelor’s degree	547	68.4
	Master’s degree	186	23.2
	Doctoral degree	8	1.0
School stage	Primary school	259	32.4
	Junior high school	284	35.5
	Senior high school	154	19.2
	9-year/complete secondary school	86	10.8
	Other	17	2.1
School location	Urban	367	45.9
	County/town	258	32.2
	Rural	175	21.9
School team coaching role	Yes	321	40.1
	No	479	59.9
Experience guiding sport-talented or high-performing students	Yes	239	29.9
	No	561	70.1

### Measures

The Self-Reported Sustainable Sport Talent Development Literacy Scale for Physical Education Teachers contained 36 first-person items across six dimensions: health protection, motivational support, education–training balance, potential cultivation, character guidance, and collaborative support. Each dimension contained six items Representative items from the six dimensions are presented in Table 2. Responses were recorded on a five-point Likert scale ranging from 1 (strongly disagree) to 5 (strongly agree). Each dimension score was calculated as the mean of its six items. Higher scores indicate stronger endorsement of the corresponding self-reported judgments and practice orientations; they should not be interpreted as direct estimates of demonstrated knowledge or professional competence. The six dimension scores are the primary interpretive units. A unit-weighted total score may be reported only in exploratory analyses. No cutoff scores were derived or validated, and neither dimension nor total scores are intended for classifying individual teachers or making decisions about them.

**Table 2:** Representative items from the six dimensions of the self-reported literacy scale.

Dimension	Representative items
Health protection	I adjust exercise load according to students’ age, fitness, and health status
	I adjust PE teaching or training arrangements according to students’ physical and psychological conditions
Motivational support	I help students understand the purpose of each PE practice task
	I help students set sport development goals that consider both effort and development outcomes
Education–training balance	I remind students to maintain a reasonable balance among training, learning, and rest
	I help students understand that sport development is not limited to competitive sport
Potential cultivation	I provide different practice tasks or development suggestions according to students’ individual differences
	I can judge which sport events or activity types may suit students according to their physical characteristics and interests
Character guidance	I guide students to learn cooperation and communication in team activities
	I pay attention to the role of physical activity in promoting students’ confidence and social adaptation
Collaborative support	I collaborate with class teachers, coaches, or school administrators to support students’ sport development
	I provide equal opportunities for sport participation and development for students of different sexes

Sustainable PE practice intention was assessed with a separate five-item measure developed for the present study. The items addressed teachers’ intentions to support long-term physical activity participation, attend to students’ health and academic development, collaborate with parents and colleagues, reduce reliance on performance-only evaluation, and provide differentiated support. The measure was included to examine the theoretically expected association between self-reported literacy and intended practice. Because it was completed by the same respondents in the same survey and covered content closely related to the focal scale, it was not treated as an independent external criterion or as a measure of observed teaching behavior. Its correlations with the focal scale were examined only as exploratory associations between related self-reported constructs

### Data analysis

Analyses were conducted in R 4.6.1 using the psych 2.6.5, lavaan 0.6–21, and semTools 0.5–9 packages. Descriptive statistics, corrected item–total correlations, inter-item correlations, and Cronbach’s alpha coefficients were calculated. The 800 retained cases were randomly divided into non-overlapping EFA and CFA subsamples of 400 using a fixed seed (20260531), providing approximately 11 cases per item in each subsample.

In the EFA subsample, sampling adequacy was evaluated using the Kaiser–Meyer–Olkin statistic and Bartlett’s test of sphericity. Parallel analysis compared the observed principal-axis reduced-correlation eigenvalues with the 95th-percentile eigenvalues generated from 100 randomized versions of the observed data and 100 random normal datasets. Principal-axis factoring was conducted on the Pearson item-correlation matrix using oblimin rotation. The number of factors was determined from the parallel analysis, and each empirical factor was matched to its prespecified domain according to the items with the salient loadings. All pattern coefficients were retained without suppression.

In the CFA subsample, the prespecified correlated six-factor model was estimated using weighted least squares mean- and variance-adjusted with delta parameterization and standardized latent-variable scaling (std.lv = TRUE). The 36 items were treated as ordered-categorical indicators. Each item loaded only on its intended factor; the factors were freely correlated, and no correlated residuals, cross-loadings, or *post hoc* modifications were introduced. Model evaluation considered scaled and robust comparative fit index (CFI), Tucker-Lewis index (TLI), and root mean square error of approximation (RMSEA), together with the standardized root mean square residual (SRMR).

Composite reliability (CR) and average variance extracted (AVE) were calculated from the standardized CFA loadings. Convergent validity evidence was evaluated from the loadings and AVE estimates, while discriminant validity was assessed using latent factor correlations and heterotrait–monotrait ratios (HTMT). A second-order model and an exploratory bifactor model were also estimated. Omega total, omega hierarchical, explained common variance (ECV), and the percentage of uncontaminated correlations were used to evaluate the plausibility of a general-score interpretation. Dimension scores were designated as the primary interpretive units, while total-score analyses were treated as exploratory.

Pearson correlations with the separate five-item sustainable PE practice-intention measure were calculated for the six dimension scores and the exploratory unit-weighted total score. These correlations were specified as exploratory associations between related self-reported constructs; no concurrent, criterion-related, or predictive-validity inference was made. Exploratory observed-score comparisons by school team coaching role and experience guiding sport-talented or high-performing students were conducted using Welch’s independent-samples t tests and Cohen’s d. These comparisons were not interpreted as latent group differences or as evidence of known-groups validity.

Sensitivity analyses re-estimated the six-factor model using theta parameterization and unweighted least squares mean- and variance-adjusted and examined its stability across 20 additional random half-samples. A theoretically plausible one-factor model was estimated for comparison. Because all core items were self-reported, Harman’s single-factor analysis and the single-factor CFA were used only to assess whether one general factor could reproduce the item covariance; they were not treated as evidence that common-method or response-style effects were absent.

Measurement-invariance analyses reused the full retained sample as a secondary analysis and were conducted across sex, school team coaching role, and school stage. Following recommendations for ordered-categorical indicators (Wu and Estabrook, 2016; Svetina et al., 2020), configural, threshold-invariance, and threshold-plus-loading models were tested sequentially using WLSMV. Thresholds were constrained before loadings, and latent means remained fixed at zero and were not compared. Changes in CFI, RMSEA, and SRMR were evaluated relative to the preceding model using approximate guidelines (Chen, 2007). Complete analysis settings, syntax, and reproducibility files are provided in the Supplementary appendices.

## Results

### Data screening and item independence

Application of the prespecified screening criteria resulted in the exclusion of 58 of the 858 submitted questionnaires: 16 duplicate submissions, 27 questionnaires completed in less than 180 s, and 15 questionnaires with straight-line responses across all 36 core items. No questionnaire was excluded solely because of response extremity. The final analytic sample comprised 800 questionnaires, corresponding to a retention rate of 93.2%. The retained dataset contained no missing or out-of-range responses. Responses to the 36 core items spanned the full 1–5 scale. No inter-item correlation exceeded 0.70, and the highest observed correlation was 0.472, indicating that excessive item redundancy was not evident. Item means ranged from 3.33 to 3.50, standard deviations from 1.08 to 1.14, and skewness values from −0.30 to −0.12. Corrected item–total correlations ranged from 0.502 to 0.615, indicating that each item was adequately related to the overall scale.

### Reliability

Cronbach’s alpha coefficients indicated acceptable to good internal consistency. Alpha values were 0.790 for health protection, 0.799 for motivational support, 0.794 for education–training balance, 0.792 for potential cultivation, 0.822 for character guidance, and 0.800 for collaborative support. Alpha for the 36-item core scale was 0.910. The five-item sustainable PE practice intention measure also showed acceptable reliability (alpha = 0.786) (Table 3).

**Table 3:** Internal consistency of the six dimensions of the self-reported literacy scale and the separate practice-intention measure.

Scale or subscale	Number of items	Cronbach’s alpha
Health protection	6	0.790
Motivational support	6	0.799
Education–training balance	6	0.794
Potential cultivation	6	0.792
Character guidance	6	0.822
Collaborative support	6	0.800
Sustainable PE practice intention	5	0.786

### Exploratory factor analysis

The EFA subsample was suitable for factor analysis (KMO = 0.916), and Bartlett’s test of sphericity was significant, *χ*^2^(630) = 4,608.316, *p* < 0.001. Parallel analysis retained six factors. The observed reduced-correlation eigenvalue for factor 6 was 0.867, above the corresponding 95th-percentile values from the randomized observed data (0.397) and random normal data (0.398); the eigenvalue for factor 7 was 0.127, below both reference values (0.361 and 0.356, respectively). Complete parallel-analysis results are provided in Appendix B.

The six-factor principal-axis solution reproduced the prespecified item-domain structure. Each of the 36 items had its largest pattern coefficient on the intended factor. Primary loadings ranged from 0.487 to 0.695, all absolute cross-loadings were 0.160 or lower, communalities ranged from 0.264 to 0.521, and uniqueness values ranged from 0.479 to 0.736. Factor correlations ranged from 0.290 to 0.447. No item was considered for removal or removed after the EFA because every item showed a clear primary loading and no problematic cross-loading. Thus, the EFA evaluated the fixed 36-item structure rather than determining the item set. Model-based fit indices were RMSR = 0.017 (df-corrected RMSR = 0.021), TLI = 0.994, and RMSEA = 0.009, 90% CI [0.000, 0.020] (Table 4).

**Table 4:** Exploratory factor analysis summary.

Indicator	Result
Subsample size	400
Correlation matrix	Pearson item-correlation matrix
KMO	0.916
Bartlett’s test	*χ*^2^(630) = 4,608.316, *p* < 0.001
Extraction	Principal-axis factoring; squared multiple correlations initial communalities
Rotation	Oblimin; gamma = 0; no Kaiser normalization
Parallel analysis	Six factors; 100 randomized + 100 normal datasets; 95th percentile
Post-EFA item removal	None
Primary loading range	0.487–0.695
Largest absolute cross-loading	0.160
Communality range	0.264–0.521
Uniqueness range	0.479–0.736
Factor-correlation range	0.290–0.447
RMSR (df-corrected)	0.017 (0.021)
TLI	0.994
RMSEA (90% CI)	0.009 [0.000, 0.020]

### Confirmatory factor analysis

The rerun reproduced the reported results in the designated CFA subsample (*n* = 400): scaled *χ*^2^(579) = 598.042, *p* = 0.283, CFI = 0.997, TLI = 0.996, RMSEA = 0.009, 90% CI [0.000, 0.019], and SRMR = 0.040. The corresponding robust indices were CFI = 0.985, TLI = 0.984, and RMSEA = 0.018, 90% CI [0.000, 0.027] (Table 5). These estimates were obtained from the CFA subsample rather than the full retained sample. The model converged after 21 iterations, produced no estimation warnings, and yielded no inadmissible residual variances. Standardized loadings ranged from 0.540 to 0.749, standardized residual variances from 0.438 to 0.708, and latent factor correlations from 0.369 to 0.572.

**Table 5:** Confirmatory factor analysis fit indices.

Model	*χ* ^2^	df	*p*	CFI	TLI	RMSEA	SRMR
Six-factor model (scaled indices)	598.042	579	0.283	0.997	0.996	0.009	0.040
Six-factor model (robust indices)	–	–	–	0.985	0.984	0.018	–

Local-fit diagnostics did not suggest that the favorable global fit was driven by a cluster of wording-related residual dependencies. The largest absolute residual correlation was 0.138 between CIS5 and HWB4. The largest normalized residuals were 3.44 between PID6 and HWB4 and −3.32 between CIS5 and HWB4. The largest residual-covariance modification index was 8.15 for HWB4 and PID6, with a standardized expected parameter change of 0.232. Within factors, the largest absolute residual correlation was 0.087 between PID5 and PID6. Residual correlations for the content-similar pairs HWB1–HWB6, HWB2–HWB4, PEB1–PEB2, PEB4–PEB6, PID1–PID2, AMS4–AMS5, and CIS3–CIS4 were all 0.060 or lower. These diagnostics were not used to free parameters or respecify the model.

The sensitivity analyses were broadly consistent with the primary results. Re-estimating the WLSMV model under theta parameterization produced the same fit indices after rounding. ULSMV with theta parameterization yielded robust CFI = 0.985, robust TLI = 0.983, robust RMSEA = 0.018, and SRMR = 0.040. Across 20 additional random half-samples of 400 participants, the median robust CFI was 0.986 (range 0.972–1.000), the median robust TLI was 0.985 (range 0.969–1.000), the median robust RMSEA was 0.017 (range 0.002–0.025), and the median SRMR was 0.039 (range 0.037–0.042). The fit estimates from the original CFA subsample fell within these ranges. In contrast, the one-factor model fit the archived CFA subsample poorly, with robust CFI = 0.604, robust TLI = 0.580, robust RMSEA = 0.090, and SRMR = 0.093.

**Figure 1:**
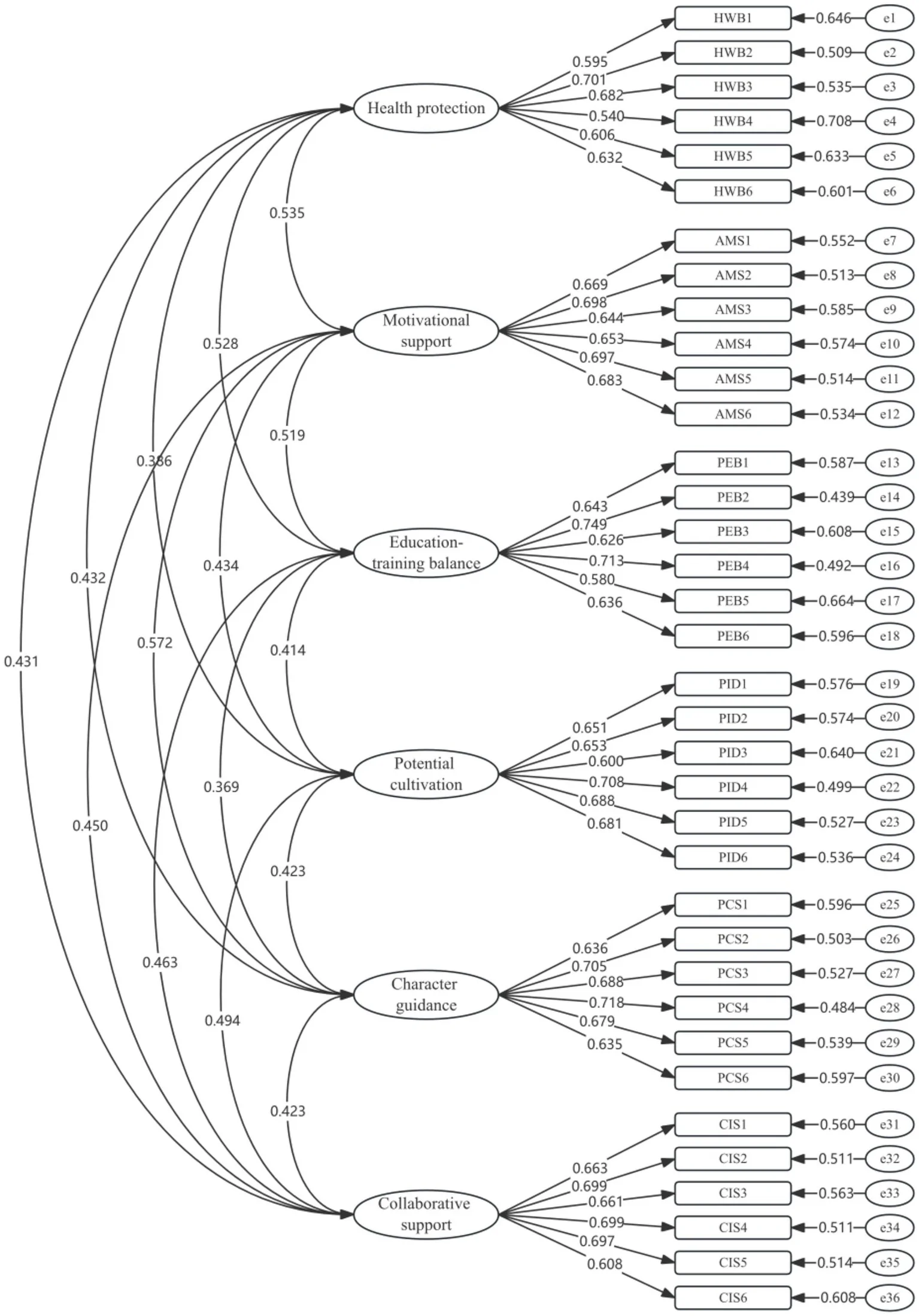
Standardized solution for the correlated six-factor model of self-reported literacy.

All standardized factor loadings were significant. Loadings ranged from 0.540 to 0.701 for health protection, 0.644 to 0.698 for motivational support, 0.580 to 0.749 for education–training balance, 0.600 to 0.708 for potential cultivation, 0.635 to 0.718 for character guidance, and 0.608 to 0.699 for collaborative support. The lowest-loading item within each dimension was HWB4 for health protection (*λ* = 0.540), AMS3 for motivational support (*λ* = 0.644), PEB5 for education–training balance (*λ* = 0.580), PID3 for potential cultivation (*λ* = 0.600), PCS6 for character guidance (*λ* = 0.635), and CIS6 for collaborative support (*λ* = 0.608). Latent factor correlations ranged from 0.369 to 0.572. Figure 1 presents the standardized six-factor solution. Full item-level estimates and confidence intervals are reported in Appendix C.

The second-order model showed acceptable fit, scaled *χ*^2^ = 638.249, df = 588, *p* = 0.074, CFI = 0.992, TLI = 0.991, RMSEA = 0.015, SRMR = 0.044; robust CFI, TLI, and RMSEA were 0.983, 0.982, and 0.019. The bifactor model also fit acceptably, scaled *χ*^2^ = 621.233, df = 558, *p* = 0.033, CFI = 0.989, TLI = 0.988, RMSEA = 0.017, SRMR = 0.042; robust CFI, TLI, and RMSEA were 0.980, 0.978, and 0.021. The bifactor indices were *ωt* = 0.939, *ωh* = 0.784, ECV = 0.459, and PUC = 0.857. Although the omega coefficients indicated substantial reliable variance at the total-score level, the ECV showed that the general factor accounted for less than half of the common variance. The scale was, therefore, not treated as essentially unidimensional. Dimension scores were treated as the primary interpretive units, while total-score analyses remained exploratory (Table 6).

**Table 6:** Higher-order model comparison.

Model	*χ*^2^ (df), *p*	CFI/TLI	RMSEA/SRMR	Robust CFI/TLI/RMSEA	General-score indices
Six-factor correlated model	598.042 (579), *p* = 0.283	0.997/0.996	0.009/0.040	0.985/0.984/0.018	–
Second-order model	638.249 (588), *p* = 0.074	0.992/0.991	0.015/0.044	0.983/0.982/0.019	*ωt* = 0.939
Bifactor model	621.233 (558), *p* = 0.033	0.989/0.988	0.017/0.042	0.980/0.978/0.021	*ωt* = 0.939; ECV = 0.459; *ωh* = 0.784; PUC = 0.857

*ωt*, omega total; *ωh*, omega hierarchical; ECV, explained common variance; PUC, percentage of uncontaminated correlations.

### Composite reliability, AVE, and discriminant evidence

CR values ranged from 0.795 to 0.836. AVE values ranged from 0.395 to 0.459, with all six estimates falling below the conventional 0.50 benchmark (Fornell and Larcker, 1981). The findings indicate limited convergent validity evidence because each factor accounted for less than half of the variance in its indicators, on average. The CR estimates support dimension-score reliability but do not offset the low AVE values. All standardized loadings were significant and exceeded 0.50, so the low AVE pattern was not attributable to a single extremely weak indicator. Further item refinement and replication in independent samples are needed before stronger claims regarding convergent validity can be made.

The moderate latent factor correlations supported discriminant validity. No latent factor correlation suggested construct redundancy. Heterotrait–monotrait ratio ranged from 0.420 to 0.556, which were below commonly used thresholds of 0.85 or 0.90. These results supported discriminant validity among the six dimensions. HTMT results are reported in Appendix D.

### Relations with sustainable PE practice intention

The separate five-item sustainable PE practice-intention measure showed acceptable internal consistency (alpha = 0.786). Its correlations with the six self-reported literacy dimensions ranged from 0.465 to 0.537. For completeness, the exploratory correlation with the unit-weighted total score was 0.727. These analyses quantified associations between closely related self-reported constructs. Because both measures were developed for this study, completed by the same respondents in the same survey, and partially overlapped in content, the correlations may reflect common-method variance and conceptual proximity. They were not interpreted as evidence of concurrent, criterion-related, or predictive validity (Table 7).

**Table 7:** Composite reliability, AVE, HTMT, and exploratory associations with sustainable PE practice intention.

Factor	CR	AVE	Highest HTMT	Exploratory correlation with the separate sustainable PE practice-intention measure
Health protection	0.795	0.395	0.514	0.509
Motivational support	0.833	0.455	0.556	0.506
Education–training balance	0.822	0.436	0.485	0.465
Potential cultivation	0.825	0.441	0.513	0.495
Character guidance	0.836	0.459	0.556	0.520
Collaborative support	0.831	0.452	0.513	0.537

CR, composite reliability; AVE, average variance extracted; HTMT, heterotrait–monotrait ratio. All AVE estimates were below 0.50, indicating limited convergent validity evidence. Correlations with the practice-intention measure represent associations between related self-reported constructs and were not interpreted as evidence of concurrent, criterion-related, or predictive validity.

The observed-score comparisons were exploratory and descriptive. The ordered-categorical invariance sequence did not test latent means or establish unbiased observed-score comparisons, and measurement invariance was not evaluated for the grouping based on experience guiding sport-talented or high-performing students. The results were, therefore, not interpreted as latent group differences. For the school team coaching role, standardized mean differences across the six dimension scores ranged from *d* = 0.063 to 0.195, and the exploratory total-score effect was *d* = 0.216. Although several comparisons reached statistical significance, the effects were trivial to small. For experience guiding sport-talented or high-performing students, dimension-level effects ranged from *d* = −0.029 to 0.073, and the exploratory total-score effect was *d* = 0.041; none of these comparisons were statistically significant. Taken together, these descriptive analyses did not establish known-groups sensitivity.

### Diagnostic checks for a single-factor account

Harman’s single-factor analysis indicated that one unrotated factor explained 22.1% of the total variance. The single-factor CFA model showed poor fit (scaled *χ*^2^ = 4,007.086, df = 594, CFI = 0.721, TLI = 0.704, RMSEA = 0.085, SRMR = 0.084). By contrast, the theoretically specified six-factor model fit substantially better in the full sample (scaled *χ*^2^ = 608.171, df = 579, CFI = 0.998, TLI = 0.997, RMSEA = 0.008, SRMR = 0.028). These findings indicate that a single-factor representation did not adequately reproduce the covariance among the 36 items. They do not rule out common-method variance, same-source bias, social desirability, acquiescent responding, or other response-style effects.

### Measurement invariance

These secondary analyses drew on the full retained sample of 800 teachers, although the analytic sample size varied across grouping variables. The sex analysis included 477 male and 310 female teachers (*N* = 787); 13 teachers who selected “other / prefer not to say” were excluded. The school team coaching-role analysis included 321 teachers with coaching responsibilities and 479 without such responsibilities (*N* = 800). The school-stage analysis compared 259 primary school teachers with 438 secondary-school teachers (*N* = 697), with junior high and senior high teachers combined as the secondary-school group. Teachers in 9-year or complete secondary schools (*n* = 86) and other school settings (*n* = 17) were excluded from this comparison.

All three configural models showed good fit, with CFI values ranging from 0.993 to 0.997, RMSEA values from 0.009 to 0.013, and SRMR values from 0.039 to 0.042. Constraining the item thresholds to equality produced little change in fit. Adding equality constraints to the factor loadings while retaining the threshold constraints likewise resulted in only small changes: across the two comparison steps, |ΔCFI| was no greater than 0.002, |ΔRMSEA| was no greater than 0.002, and |ΔSRMR| was no greater than 0.001. The findings were, therefore, consistent with configural, threshold, and threshold-plus-loading invariance across sex, school team coaching role, and school stage. This conclusion is limited to the parameters tested. The analyses do not establish full measurement equivalence, scalar invariance in the conventional continuous-indicator sense, latent-mean comparability, or unbiased comparison of observed scores. Latent means were fixed at 0 and were not compared (Table 8).

**Table 8:** Ordered-categorical measurement-invariance models.

Group comparison	Model	*χ*^2^ (df)	CFI/TLI	RMSEA/SRMR	ΔCFI/ΔRMSEA/ΔSRMR
Sex	Configural	1,191.842 (1,158)	0.997/0.997	0.009/0.039	–
	Threshold	1,266.384 (1,230)	0.997/0.997	0.009/0.039	0.000/0.000/0.000
	Threshold + loading	1,294.069 (1,260)	0.997/0.997	0.008/0.040	0.000/0.000/0.001
School team coaching role	Configural	1,239.826 (1,158)	0.993/0.993	0.013/0.041	–
	Threshold	1,310.131 (1,230)	0.994/0.993	0.013/0.041	0.000/−0.001/0.000
	Threshold + loading	1,319.871 (1,260)	0.995/0.995	0.011/0.041	0.002/−0.002/0.000
School stage	Configural	1,210.927 (1,158)	0.995/0.994	0.011/0.042	–
	Threshold	1,280.700 (1,230)	0.995/0.995	0.011/0.042	0.000/−0.001/0.000
	Threshold + loading	1,293.543 (1,260)	0.997/0.997	0.009/0.042	0.002/−0.002/0.000

All models were estimated using WLSMV with delta parameterization and standardized latent-variable scaling (std.lv = TRUE). The nested sequence comprised a configural model, a model with equal item thresholds, and a model with equal thresholds and equal factor loadings; both sets of equality constraints were retained in the final model. The reference groups were male teachers, teachers without a school team coaching role, and primary school teachers. Latent means were fixed at 0 in every model. No between-group tests of item intercepts, residual variances, latent means, or latent covariances were conducted. Analytic sample sizes were 787 for sex, 800 for school team coaching role, and 697 for school stage. Changes in fit indices were calculated relative to the preceding model using unrounded estimates.

## Discussion

The present study identified six related dimensions within PE teachers’ self-reported sustainable sport talent development literacy: health protection, motivational support, education–training balance, potential cultivation, character guidance, and collaborative support. These dimensions summarize how teachers describe their judgments and practice orientations across six areas of school-based sport talent development. They should not be interpreted as direct evidence that respondents possess or demonstrate the corresponding competencies. The content of the six dimensions is consistent with research emphasizing long-term development, appropriate support, communication, and coordination among stakeholders in youth sport (Martindale et al., 2010; Li et al., 2015; Andronikos et al., 2017). Compared with measures of athletes’ perceptions of talent development environments (Li et al., 2015; Andronikos et al., 2017) and broader PE teacher literacy instruments (Zeng et al., 2022; Li et al., 2026), the present scale focuses specifically on teachers’ self-reported judgments and practice orientations for sustainable sport talent development in school settings.

The six dimensions cover related but distinct areas of perceived professional responsibility. Health protection, motivational support, and education–training balance concern the conditions under which students can participate in sport without compromising their broader developmental needs. Potential cultivation, character guidance, and collaborative support address teachers’ reported orientations toward recognizing individual development opportunities, supporting personal and social growth, and coordinating resources within the school environment. This structure is consistent with developmental accounts that view sport potential as emerging through interactions among individual characteristics, learning opportunities, and social contexts (Côté and Vierimaa, 2014; Varghese et al., 2022). It also aligns with positive youth development research highlighting the contribution of structured sport experiences and supportive adults to young people’s personal and social development outcomes (Holt et al., 2017).

The psychometric evidence was stronger for the six-factor structure and score reliability than for convergent validity within the dimensions. The correlated six-factor model showed good global fit, and the latent correlations and HTMT ratios did not indicate redundancy among the dimensions. However, all AVE estimates remained below the conventional 0.50 benchmark. Global fit and AVE address different properties of a measurement model: global fit concerns how closely the model reproduces the observed association structure, whereas AVE reflects the proportion of indicator variance attributable to each factor. Favorable global fit, therefore, does not offset the limited convergent validity evidence. Although the local-fit diagnostics did not reveal large residual correlations among content-similar item pairs, response-style effects cannot be excluded because all items were positively worded. Further item refinement and independent replication are needed before stronger evidence of convergent validity can be claimed.

The association with sustainable PE practice intention was compatible with the theoretical basis of the focal scale. Research on self-determination, positive youth development, and talent development environments links supportive teaching orientations with adaptive developmental practices (Vasconcellos et al., 2020; Holt et al., 2017; Martindale et al., 2010). This literature provides a plausible context for the observed association between teachers’ self-reported judgments and their stated intentions. The two measures, however, were completed by the same respondents within the same survey and covered closely related content. The association is, therefore, best understood as preliminary evidence of theoretical consistency, not as evidence of concurrent, criterion-related, or predictive validity.

The exploratory observed-score comparisons provided little evidence that the scale differentiated teachers according to professional experience. Differences associated with school team coaching were trivial to small, whereas teachers with and without experience guiding sport-talented or high-performing students reported essentially similar scores. These comparisons should not be interpreted as latent group differences or as evidence of known-groups validity. One possible explanation is that experience with high-performing students in Chinese schools may emphasize training and competition rather than the broader developmental orientations covered by the scale. This interpretation remains tentative because the duration, intensity, and content of teachers’ relevant responsibilities were not measured.

The higher-order and bifactor analyses suggested that the six dimensions share a general component while retaining meaningful domain-specific variance. Omega coefficients supported the reliability of a general score, but the ECV indicated that the general factor accounted for less than half of the common variance. The six dimension scores should, therefore, remain the primary interpretive units, while total-score analyses should be treated as exploratory. This interpretation is consistent with the conceptual breadth of the instrument, which covers several related areas of self-reported judgment and practice orientations rather than a narrowly homogeneous attribute.

The secondary measurement-invariance analyses were consistent with configural, threshold, and threshold-plus-loading invariance across sex, school team coaching role, and school stage. This evidence indicates that the tested category thresholds and item–factor relations were broadly comparable across these groups. It does not establish latent-mean comparability or guarantee unbiased observed-score comparisons because latent means were not tested and stronger equality constraints were not evaluated. Independent replication and invariance models tailored to the intended comparisons are required before the scale is used to support substantive between-group conclusions.

### Intended use and interpretive limits

The intended use of the instrument should remain limited to exploratory research, aggregate description, hypothesis generation, and preliminary group-level assessment of areas for teacher development. The present evidence does not support high-stakes evaluation or decisions about individual teachers. Specifically, the scale should not be used for teacher certification; decisions about employment or promotion; performance appraisal; ranking individual teachers; labeling teachers as deficient; diagnostic classification; or establishing or applying cutoff scores. Any group-level interpretation should be descriptive and developmental rather than evaluative.

The six dimension scores are the primary interpretive units, whereas the total score remains exploratory. Broader use would require independent evidence of test–retest reliability, external validity, correspondence with observed behavior, cross-cultural validity, and sensitivity to change. Until these properties have been established independently, use should remain confined to exploratory research and aggregate-level teacher-development applications; the scale should not be used for high-stakes evaluation or individual teacher-level diagnostic decisions.

### Limitations and future research

Several limitations should be acknowledged. First, the scale measures self-reported literacy, operationalized as teachers’ endorsements of judgment and practice-orientation statements. It does not directly assess objective knowledge, demonstrated judgment, professional competence, or observed teaching behavior. The scores may also be influenced by social desirability, acquiescent responding, and other same-source effects. The separate practice-intention measure was completed by the same respondents in the same survey and covered closely related content. Its association with the focal scale should, therefore, be interpreted as preliminary evidence of theoretical consistency rather than as external validation. Future studies should compare scale scores with knowledge tests, performance-based assessments, classroom observations, independent ratings, and developmental outcomes assessed from other sources.

Second, the current evidence is based on a convenience sample of PE teachers recruited through professional networks in China. The sample may not fully represent PE teachers across different regions, school contexts, or levels of involvement in sport talent development. In addition, the known-groups analysis relied on broad experience categories and did not capture the duration, intensity, or quality of teachers’ involvement in supporting talented or high-performing students. More precise classifications of teachers’ developmental roles may provide a clearer test of group differences.

Third, the rerun and sensitivity analyses supported the reproducibility of the six-factor solution, but they do not resolve all measurement concerns. All items were positively worded, so the present design cannot separate acquiescent responding from substantive covariance. Although content-similar item pairs did not show large residual correlations, several normalized residuals were non-zero, and the AVE values remained below 0.50. Future studies should test revised or balanced item wording, model response-style effects directly, and replicate the CFA in independent samples using external and behavioral criteria.

Fourth, the measurement-invariance analyses reused the full retained sample after the cases had been divided for the primary EFA and CFA. These analyses should, therefore, be regarded as secondary full-sample evaluations rather than independent confirmatory tests. They examined only configural, threshold, and threshold-plus-loading invariance. Because latent means remained fixed and no equality tests were conducted for residual variances or other parameters required for stronger score-comparison claims, the results do not establish latent-mean comparability or unbiased observed-score comparisons. Future research should replicate the ordered-categorical invariance sequence in an independent sample and extend it according to the specific group comparisons to be undertaken.

### Conclusion

This study developed and conducted an initial psychometric evaluation of the Self-Reported Sustainable Sport Talent Development Literacy Scale for Physical Education Teachers. The results supported a six-factor structure comprising health protection, motivational support, education–training balance, potential cultivation, character guidance, and collaborative support, and the six dimension scores showed acceptable internal consistency. Secondary analyses were consistent with configural, threshold, and threshold-plus-loading invariance across sex, school team coaching role, and school stage, although they did not establish latent-mean comparability or unbiased observed-score comparisons. The scale provides a framework for examining how PE teachers report their judgments and practice orientations regarding sustainable sport talent development. Its scores should not be interpreted as direct measures of demonstrated competence or observed practice. Independent replication using external criteria, behavioral assessments, and longitudinal designs is needed before stronger evidence of validity or group-comparison claims can be made. Accordingly, the scale should not currently be used for high-stakes evaluation or individual teacher-level diagnostic decisions, and no cutoff scores should be applied.

## Data Availability

The raw data supporting the conclusions of this article will be made available by the authors, without undue reservation.
