# Supplementary material for: Development and initial psychometric evaluation of a self-reported sustainable sport talent development literacy scale for physical education teachers
Source: Front Psychol. 2026 Aug 13;17:1902630. doi: 10.3389/fpsyg.2026.1902630 (PMC13518115; doi:10.3389/fpsyg.2026.1902630)
Supplement: Supplementary file 1 [file Data_Sheet_1.ZIP › Supplementary_Appendices_A-I/Appendix A Self-report scale and separate practice-intention measure.docx]

Appendix A. Item Wording for the 36-Item Self-Report Scale and the Separate Sustainable PE Practice-Intention Measure

# A1. Self-Reported Sustainable Sport Talent Development Literacy Scale for Physical Education Teachers (36 Items)

Administration and scoring. Respondents rate each item from 1 (strongly disagree) to 5 (strongly agree). Calculate each dimension score as the mean of the six items assigned to that dimension. The six dimension scores are the primary interpretive units. A unit-weighted total score, if reported, should be treated as exploratory. No cutoff scores have been validated or should be proposed. Scores should not be used to classify, rank, diagnose, or support high-stakes decisions about individual teachers.

# Health protection

**HWB1.** I pay attention to students' physical fatigue and recovery during PE teaching or training.

**HWB2.** I adjust exercise load according to students' age, fitness, and health status.

**HWB3.** I can identify students' anxiety, resistance, or other psychological stress responses during PE learning or training.

**HWB4.** I adjust PE teaching or training arrangements according to students' physical and psychological condition.

**HWB5.** I remind students to prevent common sport injuries during physical activity.

**HWB6.** I pay attention to fatigue, aversion, or burnout that may result from sustained high-intensity practice.

# Motivational support

**AMS1.** I encourage students to choose suitable physical activities according to their interests.

**AMS2.** I help students understand the purpose of each PE practice task.

**AMS3.** I help students set sport development goals that consider both effort processes and development outcomes.

**AMS4.** I use positive feedback to strengthen students' confidence in continuing physical activity.

**AMS5.** I use encouragement and guidance to promote students' progress rather than simply comparing students' performance.

**AMS6.** I help students feel accepted and supported during physical activity.

# Education-training balance

**PEB1.** I consider students' academic development when cultivating their sport ability.

**PEB2.** I remind students to maintain a reasonable balance among training, learning, and rest.

**PEB3.** When students' sport performance improves rapidly, I still pay attention to their life adjustment and psychological state.

**PEB4.** I avoid evaluating students' sport development value only by competition results.

**PEB5.** I help students understand that sport development is not limited to competitive sport.

**PEB6.** I pay attention to the long-term developmental meaning of students' sport participation rather than only short-term performance.

# Potential cultivation

**PID1.** I can identify students' sport potential from multiple aspects, including sport skills, learning attitudes, and developmental possibilities.

**PID2.** I do not judge students' future sport development only by their current performance.

**PID3.** I provide different practice tasks or development suggestions according to students' individual differences.

**PID4.** I can judge sport events or activity types that may suit students according to their physical characteristics and interests.

**PID5.** I pay attention to students who are not currently outstanding but show persistence and learning ability.

**PID6.** I avoid having students concentrate too early or too intensively on training for a single sport.

# Character guidance

**PCS1.** I cultivate students' rule awareness and fair play through physical activity.

**PCS2.** I guide students to learn cooperation and communication in team activities.

**PCS3.** I treat failure and setbacks as important learning content in PE.

**PCS4.** I guide students to respect peers with different ability levels.

**PCS5.** I cultivate students' responsibility and self-management through physical activity.

**PCS6.** I pay attention to the role of physical activity in promoting students' confidence and social adaptation.

# Collaborative support

**CIS1.** I actively communicate with parents about students' sport interests, exercise load, and developmental needs.

**CIS2.** I collaborate with class teachers, coaches, or school administrators to support students' sport development.

**CIS3.** I provide suitable physical activity opportunities for students with different physical conditions and sport abilities.

**CIS4.** I adjust activities according to students' physical condition, sport foundation, or special needs.

**CIS5.** When students experience exclusion or labeling in physical activity, I intervene actively.

**CIS6.** I provide equal opportunities for sport participation and development for students of different genders.

# A2. Separate Sustainable PE Practice-Intention Measure (5 Items)

Note. These five items form a separate measure developed for the present study. They assess intended future practice rather than the focal self-reported literacy construct. Because both measures were completed by the same respondents in the same survey and cover closely related content, their correlations were examined only as exploratory associations between related self-reports and were not treated as criterion-related validity evidence.

# Sustainable PE practice intention

**SPEI1.** I am willing to give greater attention to students' long-term participation in physical activity in future PE teaching.

**SPEI2.** When I identify students with sport potential, I am willing to attend to their physical and mental health and academic development.

**SPEI3.** I am willing to work with parents and other teachers to support students' sport development.

**SPEI4.** I am willing to reduce single performance-based evaluation in PE teaching.

**SPEI5.** I am willing to provide differentiated sport development support for students with different ability levels.
