# Supplementary material for: Development and initial psychometric evaluation of a self-reported sustainable sport talent development literacy scale for physical education teachers
Source: Front Psychol. 2026 Aug 13;17:1902630. doi: 10.3389/fpsyg.2026.1902630 (PMC13518115; doi:10.3389/fpsyg.2026.1902630)
Supplement: Supplementary file 1 [file Data_Sheet_1.ZIP › Supplementary_Appendices_A-I/Appendix B Item-level EFA pattern loadings.docx]

**Appendix B. Complete exploratory factor analysis output**

The 36-item structure, item-to-domain assignments, and domain labels were fixed before the formal sample was analyzed. Parallel analysis determined the number of factors. The theoretical model was used to interpret the rotated solution, not to override factor retention. No item was considered for removal or removed after the EFA.

**B1. Analysis settings and fit**

| **Setting** | **Specification** |
| --- | --- |
| Data and split | Formal sample N = 800; fixed random split seed 20260531; EFA n = 400 |
| Software | R 4.6.1; psych 2.6.5; GPArotation 2026.6-1 |
| Input correlations | Pearson product-moment; pairwise.complete.obs; no EFA item responses were missing |
| Sampling adequacy | KMO = .915770; Bartlett χ²(630) = 4608.315694, p < .001 |
| Parallel analysis | Principal-axis reduced-correlation eigenvalues; 100 randomized observed datasets and 100 random normal datasets; 95th-percentile criterion; seed 20260531 |
| Extraction | Principal axis factoring (fm = "pa"); squared multiple correlations as initial communalities (SMC = TRUE); six factors; max.iter = 1000 |
| Rotation | Oblimin: gamma = 0; normalize = FALSE; randomStarts = 0; algorithm = "bb"; fwindow = 10 |
| Loading display | Pattern coefficients shown without suppression; intended primary coefficients are bold |
| Fit | RMSR = .017454; df-corrected RMSR = .021151; TLI = .994453; RMSEA = .008958, 90% CI [.000000, .020067] |
| Item retention | No item was considered for removal or removed after the formal-sample EFA |

**B2. Parallel-analysis results**

A factor was retained only when its observed reduced-correlation eigenvalue exceeded both 95th-percentile reference values. The criterion retained factors 1-6 and rejected factors 7-36.

| **Factor** | **Observed** | **Randomized data: 95th percentile** | **Normal data: 95th percentile** | **Retained** |
| --- | --- | --- | --- | --- |
| 1 | 8.322 | .742 | .739 | Yes |
| 2 | 1.344 | .621 | .611 | Yes |
| 3 | 1.244 | .544 | .538 | Yes |
| 4 | 1.064 | .498 | .490 | Yes |
| 5 | .913 | .447 | .440 | Yes |
| 6 | .867 | .397 | .398 | Yes |
| 7 | .127 | .361 | .356 | No |
| 8 | .098 | .332 | .326 | No |
| 9 | .066 | .288 | .289 | No |
| 10 | .058 | .254 | .256 | No |
| 11 | .043 | .226 | .220 | No |
| 12 | -.002 | .192 | .191 | No |
| 13 | -.034 | .159 | .158 | No |
| 14 | -.053 | .130 | .128 | No |
| 15 | -.095 | .111 | .104 | No |
| 16 | -.105 | .079 | .080 | No |
| 17 | -.117 | .049 | .050 | No |
| 18 | -.134 | .029 | .027 | No |
| 19 | -.141 | .002 | .000 | No |
| 20 | -.160 | -.025 | -.025 | No |
| 21 | -.170 | -.053 | -.051 | No |
| 22 | -.185 | -.073 | -.076 | No |
| 23 | -.217 | -.101 | -.101 | No |
| 24 | -.238 | -.121 | -.124 | No |
| 25 | -.259 | -.149 | -.147 | No |
| 26 | -.274 | -.174 | -.171 | No |
| 27 | -.289 | -.198 | -.191 | No |
| 28 | -.297 | -.220 | -.212 | No |
| 29 | -.319 | -.244 | -.234 | No |
| 30 | -.342 | -.259 | -.263 | No |
| 31 | -.352 | -.286 | -.285 | No |
| 32 | -.372 | -.317 | -.314 | No |
| 33 | -.379 | -.343 | -.333 | No |
| 34 | -.420 | -.365 | -.355 | No |
| 35 | -.427 | -.395 | -.391 | No |
| 36 | -.441 | -.425 | -.422 | No |

**B3. Complete oblimin pattern matrix**

HP = health protection; MS = motivational support; ETB = education-training balance; PC = potential cultivation; CG = character guidance; CS = collaborative support; h² = communality; u² = uniqueness. All 216 pattern coefficients are shown.

| **Item** | **HP** | **MS** | **ETB** | **PC** | **CG** | **CS** | **h²** | **u²** |
| --- | --- | --- | --- | --- | --- | --- | --- | --- |
| HWB1 | **.614** | .105 | -.022 | -.028 | -.016 | .089 | .447 | .553 |
| HWB2 | **.593** | -.006 | .011 | .018 | .146 | .008 | .467 | .533 |
| HWB3 | **.563** | -.055 | .052 | .014 | .093 | .056 | .404 | .596 |
| HWB4 | **.629** | .074 | -.028 | -.001 | .015 | -.002 | .433 | .567 |
| HWB5 | **.607** | .026 | .032 | .092 | -.064 | -.007 | .404 | .596 |
| HWB6 | **.579** | -.059 | .092 | .018 | .059 | -.069 | .373 | .627 |
| AMS1 | -.024 | **.606** | .037 | -.008 | -.021 | .056 | .382 | .618 |
| AMS2 | -.077 | **.546** | -.020 | .132 | .052 | -.027 | .346 | .654 |
| AMS3 | .001 | **.574** | .020 | .005 | -.014 | .012 | .337 | .663 |
| AMS4 | .028 | **.695** | .083 | -.008 | .022 | -.067 | .521 | .479 |
| AMS5 | .041 | **.527** | .070 | .075 | .003 | .038 | .388 | .612 |
| AMS6 | .107 | **.574** | -.160 | -.023 | .062 | .081 | .409 | .591 |
| PEB1 | .066 | .024 | **.550** | .085 | -.053 | .016 | .371 | .629 |
| PEB2 | -.065 | .096 | **.550** | -.089 | .092 | .117 | .388 | .612 |
| PEB3 | -.005 | .006 | **.647** | .046 | -.075 | .014 | .416 | .584 |
| PEB4 | .027 | -.008 | **.606** | -.009 | .042 | .075 | .429 | .571 |
| PEB5 | .049 | -.010 | **.605** | .086 | .002 | -.078 | .405 | .595 |
| PEB6 | .035 | .027 | **.549** | -.015 | .145 | -.001 | .405 | .595 |
| PID1 | .077 | -.063 | -.034 | **.487** | -.021 | .086 | .264 | .736 |
| PID2 | .035 | .070 | -.002 | **.565** | -.046 | .095 | .399 | .601 |
| PID3 | .039 | -.012 | .137 | **.496** | .070 | -.015 | .369 | .631 |
| PID4 | -.011 | .022 | .069 | **.610** | .093 | .018 | .491 | .509 |
| PID5 | -.059 | .059 | -.012 | **.607** | .104 | -.034 | .414 | .586 |
| PID6 | .058 | .014 | -.001 | **.618** | -.055 | .040 | .408 | .592 |
| PCS1 | .067 | .018 | .054 | -.052 | **.642** | .034 | .488 | .512 |
| PCS2 | -.046 | .038 | .044 | -.019 | **.679** | .058 | .511 | .489 |
| PCS3 | .052 | .017 | -.019 | .059 | **.648** | -.025 | .476 | .524 |
| PCS4 | .024 | .085 | .072 | .014 | **.532** | .026 | .409 | .591 |
| PCS5 | .106 | .061 | .012 | .082 | **.544** | .000 | .459 | .541 |
| PCS6 | .019 | -.052 | -.053 | .096 | **.634** | .032 | .438 | .562 |
| CIS1 | -.083 | -.016 | .002 | .042 | .007 | **.669** | .441 | .559 |
| CIS2 | .116 | .043 | .010 | -.024 | .048 | **.554** | .403 | .597 |
| CIS3 | .142 | .025 | .035 | .051 | -.094 | **.614** | .453 | .547 |
| CIS4 | .057 | -.004 | .058 | -.032 | .012 | **.559** | .349 | .651 |
| CIS5 | -.031 | .017 | -.006 | .022 | .117 | **.556** | .385 | .615 |
| CIS6 | -.099 | .004 | .022 | .076 | .096 | **.561** | .393 | .607 |

**B4. Factor correlations**

| **Factor** | **HP** | **MS** | **ETB** | **PC** | **CG** | **CS** |
| --- | --- | --- | --- | --- | --- | --- |
| Health protection | 1.000 | .381 | .325 | .347 | .447 | .290 |
| Motivational support | .381 | 1.000 | .309 | .370 | .444 | .345 |
| Education-training balance | .325 | .309 | 1.000 | .402 | .378 | .313 |
| Potential cultivation | .347 | .370 | .402 | 1.000 | .430 | .399 |
| Character guidance | .447 | .444 | .378 | .430 | 1.000 | .437 |
| Collaborative support | .290 | .345 | .313 | .399 | .437 | 1.000 |

**B5. Exact reproducible syntax**

set.seed(20260531)

idx <- sample(seq_len(nrow(dat)))

efa_dat <- dat[idx[1:400], items]

R <- cor(efa_dat, use = "pairwise.complete.obs", method = "pearson")

set.seed(20260531)

pa <- psych::fa.parallel(efa_dat, fa = "fa", fm = "pa", n.iter = 100,

cor = "cor", quant = .95, plot = FALSE,

error.bars = FALSE)

efa <- psych::fa(R, nfactors = 6, n.obs = 400, fm = "pa",

rotate = "oblimin", SMC = TRUE, residuals = TRUE,

max.iter = 1000)

\# GPArotation::oblimin defaults used by psych::fa:

\# gam = 0; normalize = FALSE; randomStarts = 0; algorithm = "bb"; fwindow = 10
