# Supplementary material for: Development and initial psychometric evaluation of a self-reported sustainable sport talent development literacy scale for physical education teachers
Source: Front Psychol. 2026 Aug 13;17:1902630. doi: 10.3389/fpsyg.2026.1902630 (PMC13518115; doi:10.3389/fpsyg.2026.1902630)
Supplement: Supplementary file 1 [file Data_Sheet_1.ZIP › Supplementary_Appendices_A-I/Appendix C Item-level CFA standardized loadings.docx]

Appendix C. Item-level CFA standardized loadings

| **Item** | **Factor** | **Loading** | **SE** | **95% CI lower** | **95% CI upper** |
| --- | --- | --- | --- | --- | --- |
| HWB1 | Health protection | .595 | .048 | .502 | .689 |
| HWB2 | Health protection | .701 | .043 | .617 | .786 |
| HWB3 | Health protection | .682 | .043 | .597 | .767 |
| HWB4 | Health protection | .540 | .052 | .439 | .642 |
| HWB5 | Health protection | .606 | .048 | .511 | .700 |
| HWB6 | Health protection | .632 | .050 | .534 | .729 |
| AMS1 | Motivational support | .669 | .041 | .588 | .750 |
| AMS2 | Motivational support | .698 | .037 | .625 | .770 |
| AMS3 | Motivational support | .644 | .041 | .563 | .725 |
| AMS4 | Motivational support | .653 | .042 | .570 | .735 |
| AMS5 | Motivational support | .697 | .039 | .621 | .773 |
| AMS6 | Motivational support | .683 | .037 | .610 | .756 |
| PEB1 | Education-training balance | .643 | .043 | .558 | .727 |
| PEB2 | Education-training balance | .749 | .039 | .672 | .827 |
| PEB3 | Education-training balance | .626 | .044 | .539 | .713 |
| PEB4 | Education-training balance | .713 | .040 | .635 | .790 |
| PEB5 | Education-training balance | .580 | .048 | .486 | .674 |
| PEB6 | Education-training balance | .636 | .045 | .549 | .723 |
| PID1 | Potential cultivation | .651 | .045 | .563 | .738 |
| PID2 | Potential cultivation | .653 | .045 | .564 | .742 |
| PID3 | Potential cultivation | .600 | .048 | .506 | .695 |
| PID4 | Potential cultivation | .708 | .041 | .628 | .788 |
| PID5 | Potential cultivation | .688 | .043 | .604 | .772 |
| PID6 | Potential cultivation | .681 | .043 | .596 | .765 |
| PCS1 | Character guidance | .636 | .040 | .558 | .715 |
| PCS2 | Character guidance | .705 | .041 | .625 | .784 |
| PCS3 | Character guidance | .688 | .041 | .608 | .768 |
| PCS4 | Character guidance | .718 | .036 | .647 | .788 |
| PCS5 | Character guidance | .679 | .043 | .595 | .764 |
| PCS6 | Character guidance | .635 | .042 | .553 | .717 |
| CIS1 | Collaborative support | .663 | .044 | .576 | .750 |
| CIS2 | Collaborative support | .699 | .040 | .622 | .777 |
| CIS3 | Collaborative support | .661 | .042 | .580 | .743 |
| CIS4 | Collaborative support | .699 | .039 | .623 | .776 |
| CIS5 | Collaborative support | .697 | .037 | .625 | .769 |
| CIS6 | Collaborative support | .608 | .047 | .515 | .700 |
