# Supplementary material for: Development and initial psychometric evaluation of a self-reported sustainable sport talent development literacy scale for physical education teachers
Source: Front Psychol. 2026 Aug 13;17:1902630. doi: 10.3389/fpsyg.2026.1902630 (PMC13518115; doi:10.3389/fpsyg.2026.1902630)
Supplement: Supplementary file 1 [file Data_Sheet_1.ZIP › Supplementary_Appendices_A-I/Appendix D HTMT ratios among six dimensions.docx]

Appendix D. HTMT ratios among the six dimensions

| **Factor** | **HWB** | **AMS** | **PEB** | **PID** | **PCS** | **CIS** |
| --- | --- | --- | --- | --- | --- | --- |
| HWB | 1.000 | .500 | .485 | .430 | .514 | .420 |
| AMS | .500 | 1.000 | .469 | .454 | .556 | .449 |
| PEB | .485 | .469 | 1.000 | .463 | .436 | .452 |
| PID | .430 | .454 | .463 | 1.000 | .493 | .513 |
| PCS | .514 | .556 | .436 | .493 | 1.000 | .492 |
| CIS | .420 | .449 | .452 | .513 | .492 | 1.000 |
