# Supplementary material for: Development and initial psychometric evaluation of a self-reported sustainable sport talent development literacy scale for physical education teachers
Source: Front Psychol. 2026 Aug 13;17:1902630. doi: 10.3389/fpsyg.2026.1902630 (PMC13518115; doi:10.3389/fpsyg.2026.1902630)
Supplement: Supplementary file 1 [file Data_Sheet_1.ZIP › Supplementary_Appendices_A-I/Appendix E Full latent correlation matrix.docx]

Appendix E. Full latent correlation matrix

| **Factor** | **HWB** | **AMS** | **PEB** | **PID** | **PCS** | **CIS** |
| --- | --- | --- | --- | --- | --- | --- |
| HWB | 1.000 | 0.535 | 0.528 | 0.386 | 0.432 | 0.431 |
| AMS | 0.535 | 1.000 | 0.519 | 0.434 | 0.572 | 0.450 |
| PEB | 0.528 | 0.519 | 1.000 | 0.414 | 0.369 | 0.463 |
| PID | 0.386 | 0.434 | 0.414 | 1.000 | 0.423 | 0.494 |
| PCS | 0.432 | 0.572 | 0.369 | 0.423 | 1.000 | 0.423 |
| CIS | 0.431 | 0.450 | 0.463 | 0.494 | 0.423 | 1.000 |
