# Supplementary material for: Development and initial psychometric evaluation of a self-reported sustainable sport talent development literacy scale for physical education teachers
Source: Front Psychol. 2026 Aug 13;17:1902630. doi: 10.3389/fpsyg.2026.1902630 (PMC13518115; doi:10.3389/fpsyg.2026.1902630)
Supplement: Supplementary file 1 [file Data_Sheet_1.ZIP › Supplementary_Appendices_A-I/Appendix F Item-level descriptive statistics.docx]

Appendix F. Item-level descriptive statistics

| **Item** | **Factor** | **M** | **SD** | **Skewness** | **Corrected item-total r** | **Highest inter-item r** |
| --- | --- | --- | --- | --- | --- | --- |
| HWB1 | HWB | 3.368 | 1.088 | -0.119 | 0.545 | 0.428 |
| HWB2 | HWB | 3.360 | 1.134 | -0.239 | 0.585 | 0.428 |
| HWB3 | HWB | 3.377 | 1.087 | -0.141 | 0.534 | 0.420 |
| HWB4 | HWB | 3.354 | 1.111 | -0.221 | 0.517 | 0.389 |
| HWB5 | HWB | 3.404 | 1.115 | -0.176 | 0.541 | 0.409 |
| HWB6 | HWB | 3.401 | 1.100 | -0.187 | 0.519 | 0.418 |
| AMS1 | AMS | 3.447 | 1.097 | -0.265 | 0.539 | 0.413 |
| AMS2 | AMS | 3.449 | 1.112 | -0.202 | 0.546 | 0.430 |
| AMS3 | AMS | 3.498 | 1.113 | -0.193 | 0.528 | 0.404 |
| AMS4 | AMS | 3.445 | 1.124 | -0.244 | 0.594 | 0.450 |
| AMS5 | AMS | 3.464 | 1.123 | -0.202 | 0.552 | 0.420 |
| AMS6 | AMS | 3.453 | 1.112 | -0.228 | 0.559 | 0.450 |
| PEB1 | PEB | 3.397 | 1.119 | -0.199 | 0.542 | 0.428 |
| PEB2 | PEB | 3.344 | 1.108 | -0.128 | 0.553 | 0.409 |
| PEB3 | PEB | 3.331 | 1.103 | -0.183 | 0.546 | 0.426 |
| PEB4 | PEB | 3.389 | 1.117 | -0.176 | 0.567 | 0.426 |
| PEB5 | PEB | 3.386 | 1.133 | -0.167 | 0.524 | 0.396 |
| PEB6 | PEB | 3.348 | 1.137 | -0.183 | 0.549 | 0.428 |
| PID1 | PID | 3.428 | 1.115 | -0.195 | 0.502 | 0.383 |
| PID2 | PID | 3.400 | 1.081 | -0.208 | 0.565 | 0.455 |
| PID3 | PID | 3.405 | 1.116 | -0.208 | 0.507 | 0.413 |
| PID4 | PID | 3.355 | 1.139 | -0.197 | 0.595 | 0.445 |
| PID5 | PID | 3.408 | 1.124 | -0.267 | 0.555 | 0.455 |
| PID6 | PID | 3.422 | 1.135 | -0.238 | 0.543 | 0.445 |
| PCS1 | PCS | 3.349 | 1.127 | -0.233 | 0.597 | 0.458 |
| PCS2 | PCS | 3.479 | 1.121 | -0.300 | 0.615 | 0.472 |
| PCS3 | PCS | 3.417 | 1.120 | -0.211 | 0.593 | 0.465 |
| PCS4 | PCS | 3.440 | 1.118 | -0.245 | 0.581 | 0.472 |
| PCS5 | PCS | 3.433 | 1.112 | -0.251 | 0.575 | 0.453 |
| PCS6 | PCS | 3.439 | 1.107 | -0.258 | 0.566 | 0.439 |
| CIS1 | CIS | 3.438 | 1.136 | -0.295 | 0.542 | 0.410 |
| CIS2 | CIS | 3.330 | 1.107 | -0.213 | 0.563 | 0.431 |
| CIS3 | CIS | 3.467 | 1.095 | -0.236 | 0.561 | 0.434 |
| CIS4 | CIS | 3.421 | 1.115 | -0.228 | 0.546 | 0.415 |
| CIS5 | CIS | 3.396 | 1.107 | -0.197 | 0.584 | 0.434 |
| CIS6 | CIS | 3.466 | 1.115 | -0.303 | 0.534 | 0.413 |
