# Supplementary material for: Development and initial psychometric evaluation of a self-reported sustainable sport talent development literacy scale for physical education teachers
Source: Front Psychol. 2026 Aug 13;17:1902630. doi: 10.3389/fpsyg.2026.1902630 (PMC13518115; doi:10.3389/fpsyg.2026.1902630)
Supplement: Supplementary file 1 [file Data_Sheet_1.ZIP › Supplementary_Appendices_A-I/Appendix G Bifactor standardized loadings.docx]

Appendix G. Bifactor standardized loadings

| **Item** | **General** | **Specific factor loading** | **Specific factor** |
| --- | --- | --- | --- |
| HWB1 | 0.401 | 0.467 | HWB |
| HWB2 | 0.482 | 0.499 | HWB |
| HWB3 | 0.484 | 0.428 | HWB |
| HWB4 | 0.363 | 0.424 | HWB |
| HWB5 | 0.407 | 0.479 | HWB |
| HWB6 | 0.437 | 0.449 | HWB |
| AMS1 | 0.527 | 0.371 | AMS |
| AMS2 | 0.530 | 0.457 | AMS |
| AMS3 | 0.484 | 0.440 | AMS |
| AMS4 | 0.482 | 0.484 | AMS |
| AMS5 | 0.544 | 0.395 | AMS |
| AMS6 | 0.511 | 0.478 | AMS |
| PEB1 | 0.420 | 0.521 | PEB |
| PEB2 | 0.530 | 0.474 | PEB |
| PEB3 | 0.419 | 0.484 | PEB |
| PEB4 | 0.499 | 0.463 | PEB |
| PEB5 | 0.375 | 0.484 | PEB |
| PEB6 | 0.415 | 0.509 | PEB |
| PID1 | 0.393 | 0.547 | PID |
| PID2 | 0.372 | 0.614 | PID |
| PID3 | 0.382 | 0.452 | PID |
| PID4 | 0.453 | 0.526 | PID |
| PID5 | 0.439 | 0.506 | PID |
| PID6 | 0.445 | 0.468 | PID |
| PCS1 | 0.389 | 0.558 | PCS |
| PCS2 | 0.464 | 0.532 | PCS |
| PCS3 | 0.461 | 0.493 | PCS |
| PCS4 | 0.473 | 0.534 | PCS |
| PCS5 | 0.466 | 0.451 | PCS |
| PCS6 | 0.411 | 0.496 | PCS |
| CIS1 | 0.462 | 0.424 | CIS |
| CIS2 | 0.466 | 0.511 | CIS |
| CIS3 | 0.437 | 0.489 | CIS |
| CIS4 | 0.479 | 0.475 | CIS |
| CIS5 | 0.427 | 0.626 | CIS |
| CIS6 | 0.386 | 0.499 | CIS |
