# Supplementary material for: Development and initial psychometric evaluation of a self-reported sustainable sport talent development literacy scale for physical education teachers
Source: Front Psychol. 2026 Aug 13;17:1902630. doi: 10.3389/fpsyg.2026.1902630 (PMC13518115; doi:10.3389/fpsyg.2026.1902630)
Supplement: Supplementary file 1 [file Data_Sheet_1.ZIP › Supplementary_Appendices_A-I/Appendix H Full item correlation matrix.docx]

Appendix H. Full item correlation matrix

The 36 x 36 correlation matrix is presented in three column blocks. Row labels remain constant across blocks.

# Block 1: HWB1 to AMS6

| **Item** | **HWB1** | **HWB2** | **HWB3** | **HWB4** | **HWB5** | **HWB6** | **AMS1** | **AMS2** | **AMS3** | **AMS4** | **AMS5** | **AMS6** |
| --- | --- | --- | --- | --- | --- | --- | --- | --- | --- | --- | --- | --- |
| HWB1 | 1.000 | .428 | .368 | .358 | .393 | .390 | .189 | .189 | .231 | .232 | .175 | .268 |
| HWB2 | .428 | 1.000 | .420 | .389 | .409 | .418 | .190 | .193 | .211 | .207 | .229 | .245 |
| HWB3 | .368 | .420 | 1.000 | .386 | .390 | .341 | .177 | .171 | .178 | .221 | .254 | .222 |
| HWB4 | .358 | .389 | .386 | 1.000 | .375 | .347 | .171 | .199 | .144 | .227 | .241 | .164 |
| HWB5 | .393 | .409 | .390 | .375 | 1.000 | .363 | .187 | .192 | .140 | .200 | .190 | .195 |
| HWB6 | .390 | .418 | .341 | .347 | .363 | 1.000 | .138 | .134 | .187 | .174 | .194 | .190 |
| AMS1 | .189 | .190 | .177 | .171 | .187 | .138 | 1.000 | .365 | .384 | .413 | .403 | .381 |
| AMS2 | .189 | .193 | .171 | .199 | .192 | .134 | .365 | 1.000 | .364 | .430 | .403 | .408 |
| AMS3 | .231 | .211 | .178 | .144 | .140 | .187 | .384 | .364 | 1.000 | .404 | .377 | .383 |
| AMS4 | .232 | .207 | .221 | .227 | .200 | .174 | .413 | .430 | .404 | 1.000 | .420 | .450 |
| AMS5 | .175 | .229 | .254 | .241 | .190 | .194 | .403 | .403 | .377 | .420 | 1.000 | .385 |
| AMS6 | .268 | .245 | .222 | .164 | .195 | .190 | .381 | .408 | .383 | .450 | .385 | 1.000 |
| PEB1 | .165 | .200 | .233 | .177 | .184 | .202 | .197 | .220 | .158 | .184 | .226 | .086 |
| PEB2 | .175 | .219 | .198 | .157 | .161 | .200 | .245 | .211 | .234 | .247 | .219 | .182 |
| PEB3 | .165 | .202 | .136 | .108 | .170 | .165 | .173 | .154 | .166 | .222 | .172 | .092 |
| PEB4 | .167 | .246 | .207 | .185 | .224 | .212 | .213 | .233 | .208 | .166 | .203 | .165 |
| PEB5 | .105 | .187 | .189 | .165 | .142 | .166 | .174 | .176 | .164 | .194 | .169 | .140 |
| PEB6 | .203 | .283 | .272 | .203 | .189 | .216 | .190 | .183 | .132 | .205 | .184 | .186 |
| PID1 | .088 | .141 | .158 | .129 | .159 | .143 | .131 | .149 | .072 | .161 | .168 | .150 |
| PID2 | .122 | .168 | .162 | .154 | .195 | .115 | .229 | .211 | .144 | .191 | .209 | .179 |
| PID3 | .155 | .220 | .187 | .221 | .149 | .169 | .196 | .145 | .154 | .173 | .214 | .191 |
| PID4 | .178 | .196 | .209 | .186 | .228 | .186 | .229 | .202 | .172 | .207 | .235 | .170 |
| PID5 | .160 | .177 | .157 | .136 | .148 | .130 | .177 | .192 | .197 | .167 | .193 | .176 |
| PID6 | .147 | .143 | .179 | .228 | .181 | .180 | .166 | .197 | .140 | .165 | .211 | .175 |
| PCS1 | .203 | .224 | .225 | .185 | .160 | .173 | .189 | .202 | .173 | .234 | .290 | .196 |
| PCS2 | .208 | .253 | .239 | .145 | .182 | .219 | .220 | .240 | .244 | .255 | .265 | .258 |
| PCS3 | .209 | .266 | .230 | .196 | .168 | .220 | .210 | .203 | .199 | .245 | .265 | .233 |
| PCS4 | .196 | .237 | .226 | .204 | .188 | .207 | .269 | .216 | .219 | .233 | .268 | .253 |
| PCS5 | .283 | .268 | .249 | .222 | .196 | .256 | .209 | .263 | .215 | .253 | .325 | .266 |
| PCS6 | .191 | .255 | .231 | .125 | .170 | .162 | .209 | .170 | .194 | .177 | .221 | .240 |
| CIS1 | .154 | .181 | .142 | .098 | .162 | .109 | .183 | .147 | .135 | .131 | .183 | .192 |
| CIS2 | .206 | .211 | .244 | .133 | .206 | .165 | .204 | .171 | .210 | .211 | .212 | .184 |
| CIS3 | .200 | .199 | .185 | .175 | .182 | .166 | .182 | .189 | .168 | .181 | .213 | .220 |
| CIS4 | .194 | .193 | .195 | .163 | .204 | .129 | .176 | .211 | .150 | .184 | .207 | .154 |
| CIS5 | .190 | .195 | .178 | .080 | .172 | .146 | .174 | .166 | .189 | .190 | .175 | .186 |
| CIS6 | .158 | .136 | .157 | .105 | .099 | .127 | .122 | .169 | .120 | .178 | .192 | .187 |

# Block 2: PEB1 to PID6

| **Item** | **PEB1** | **PEB2** | **PEB3** | **PEB4** | **PEB5** | **PEB6** | **PID1** | **PID2** | **PID3** | **PID4** | **PID5** | **PID6** |
| --- | --- | --- | --- | --- | --- | --- | --- | --- | --- | --- | --- | --- |
| HWB1 | .165 | .175 | .165 | .167 | .105 | .203 | .088 | .122 | .155 | .178 | .160 | .147 |
| HWB2 | .200 | .219 | .202 | .246 | .187 | .283 | .141 | .168 | .220 | .196 | .177 | .143 |
| HWB3 | .233 | .198 | .136 | .207 | .189 | .272 | .158 | .162 | .187 | .209 | .157 | .179 |
| HWB4 | .177 | .157 | .108 | .185 | .165 | .203 | .129 | .154 | .221 | .186 | .136 | .228 |
| HWB5 | .184 | .161 | .170 | .224 | .142 | .189 | .159 | .195 | .149 | .228 | .148 | .181 |
| HWB6 | .202 | .200 | .165 | .212 | .166 | .216 | .143 | .115 | .169 | .186 | .130 | .180 |
| AMS1 | .197 | .245 | .173 | .213 | .174 | .190 | .131 | .229 | .196 | .229 | .177 | .166 |
| AMS2 | .220 | .211 | .154 | .233 | .176 | .183 | .149 | .211 | .145 | .202 | .192 | .197 |
| AMS3 | .158 | .234 | .166 | .208 | .164 | .132 | .072 | .144 | .154 | .172 | .197 | .140 |
| AMS4 | .184 | .247 | .222 | .166 | .194 | .205 | .161 | .191 | .173 | .207 | .167 | .165 |
| AMS5 | .226 | .219 | .172 | .203 | .169 | .184 | .168 | .209 | .214 | .235 | .193 | .211 |
| AMS6 | .086 | .182 | .092 | .165 | .140 | .186 | .150 | .179 | .191 | .170 | .176 | .175 |
| PEB1 | 1.000 | .387 | .371 | .381 | .377 | .428 | .104 | .196 | .155 | .189 | .168 | .220 |
| PEB2 | .387 | 1.000 | .390 | .409 | .396 | .396 | .137 | .181 | .186 | .238 | .218 | .199 |
| PEB3 | .371 | .390 | 1.000 | .426 | .386 | .384 | .128 | .167 | .144 | .183 | .169 | .210 |
| PEB4 | .381 | .409 | .426 | 1.000 | .389 | .416 | .162 | .173 | .242 | .232 | .169 | .211 |
| PEB5 | .377 | .396 | .386 | .389 | 1.000 | .342 | .160 | .166 | .215 | .213 | .161 | .217 |
| PEB6 | .428 | .396 | .384 | .416 | .342 | 1.000 | .085 | .146 | .199 | .185 | .170 | .209 |
| PID1 | .104 | .137 | .128 | .162 | .160 | .085 | 1.000 | .360 | .328 | .383 | .371 | .369 |
| PID2 | .196 | .181 | .167 | .173 | .166 | .146 | .360 | 1.000 | .371 | .433 | .455 | .390 |
| PID3 | .155 | .186 | .144 | .242 | .215 | .199 | .328 | .371 | 1.000 | .413 | .355 | .360 |
| PID4 | .189 | .238 | .183 | .232 | .213 | .185 | .383 | .433 | .413 | 1.000 | .424 | .445 |
| PID5 | .168 | .218 | .169 | .169 | .161 | .170 | .371 | .455 | .355 | .424 | 1.000 | .375 |
| PID6 | .220 | .199 | .210 | .211 | .217 | .209 | .369 | .390 | .360 | .445 | .375 | 1.000 |
| PCS1 | .165 | .187 | .141 | .160 | .155 | .176 | .164 | .174 | .203 | .218 | .214 | .162 |
| PCS2 | .181 | .257 | .158 | .223 | .194 | .249 | .196 | .155 | .177 | .221 | .199 | .152 |
| PCS3 | .131 | .217 | .139 | .189 | .126 | .202 | .240 | .178 | .246 | .269 | .258 | .177 |
| PCS4 | .194 | .227 | .184 | .198 | .139 | .230 | .182 | .162 | .194 | .252 | .255 | .186 |
| PCS5 | .202 | .205 | .171 | .205 | .153 | .175 | .182 | .190 | .198 | .252 | .242 | .242 |
| PCS6 | .161 | .194 | .131 | .140 | .110 | .208 | .153 | .174 | .216 | .239 | .193 | .191 |
| CIS1 | .183 | .208 | .134 | .210 | .099 | .155 | .167 | .236 | .194 | .233 | .221 | .222 |
| CIS2 | .164 | .251 | .220 | .213 | .157 | .161 | .173 | .214 | .151 | .225 | .202 | .215 |
| CIS3 | .199 | .211 | .141 | .229 | .142 | .202 | .219 | .249 | .222 | .243 | .243 | .199 |
| CIS4 | .196 | .201 | .199 | .218 | .166 | .187 | .191 | .211 | .158 | .194 | .152 | .170 |
| CIS5 | .144 | .224 | .168 | .192 | .147 | .165 | .147 | .188 | .156 | .228 | .223 | .212 |
| CIS6 | .177 | .168 | .144 | .173 | .168 | .130 | .213 | .199 | .201 | .207 | .214 | .191 |

# Block 3: PCS1 to CIS6

| **Item** | **PCS1** | **PCS2** | **PCS3** | **PCS4** | **PCS5** | **PCS6** | **CIS1** | **CIS2** | **CIS3** | **CIS4** | **CIS5** | **CIS6** |
| --- | --- | --- | --- | --- | --- | --- | --- | --- | --- | --- | --- | --- |
| HWB1 | .203 | .208 | .209 | .196 | .283 | .191 | .154 | .206 | .200 | .194 | .190 | .158 |
| HWB2 | .224 | .253 | .266 | .237 | .268 | .255 | .181 | .211 | .199 | .193 | .195 | .136 |
| HWB3 | .225 | .239 | .230 | .226 | .249 | .231 | .142 | .244 | .185 | .195 | .178 | .157 |
| HWB4 | .185 | .145 | .196 | .204 | .222 | .125 | .098 | .133 | .175 | .163 | .080 | .105 |
| HWB5 | .160 | .182 | .168 | .188 | .196 | .170 | .162 | .206 | .182 | .204 | .172 | .099 |
| HWB6 | .173 | .219 | .220 | .207 | .256 | .162 | .109 | .165 | .166 | .129 | .146 | .127 |
| AMS1 | .189 | .220 | .210 | .269 | .209 | .209 | .183 | .204 | .182 | .176 | .174 | .122 |
| AMS2 | .202 | .240 | .203 | .216 | .263 | .170 | .147 | .171 | .189 | .211 | .166 | .169 |
| AMS3 | .173 | .244 | .199 | .219 | .215 | .194 | .135 | .210 | .168 | .150 | .189 | .120 |
| AMS4 | .234 | .255 | .245 | .233 | .253 | .177 | .131 | .211 | .181 | .184 | .190 | .178 |
| AMS5 | .290 | .265 | .265 | .268 | .325 | .221 | .183 | .212 | .213 | .207 | .175 | .192 |
| AMS6 | .196 | .258 | .233 | .253 | .266 | .240 | .192 | .184 | .220 | .154 | .186 | .187 |
| PEB1 | .165 | .181 | .131 | .194 | .202 | .161 | .183 | .164 | .199 | .196 | .144 | .177 |
| PEB2 | .187 | .257 | .217 | .227 | .205 | .194 | .208 | .251 | .211 | .201 | .224 | .168 |
| PEB3 | .141 | .158 | .139 | .184 | .171 | .131 | .134 | .220 | .141 | .199 | .168 | .144 |
| PEB4 | .160 | .223 | .189 | .198 | .205 | .140 | .210 | .213 | .229 | .218 | .192 | .173 |
| PEB5 | .155 | .194 | .126 | .139 | .153 | .110 | .099 | .157 | .142 | .166 | .147 | .168 |
| PEB6 | .176 | .249 | .202 | .230 | .175 | .208 | .155 | .161 | .202 | .187 | .165 | .130 |
| PID1 | .164 | .196 | .240 | .182 | .182 | .153 | .167 | .173 | .219 | .191 | .147 | .213 |
| PID2 | .174 | .155 | .178 | .162 | .190 | .174 | .236 | .214 | .249 | .211 | .188 | .199 |
| PID3 | .203 | .177 | .246 | .194 | .198 | .216 | .194 | .151 | .222 | .158 | .156 | .201 |
| PID4 | .218 | .221 | .269 | .252 | .252 | .239 | .233 | .225 | .243 | .194 | .228 | .207 |
| PID5 | .214 | .199 | .258 | .255 | .242 | .193 | .221 | .202 | .243 | .152 | .223 | .214 |
| PID6 | .162 | .152 | .177 | .186 | .242 | .191 | .222 | .215 | .199 | .170 | .212 | .191 |
| PCS1 | 1.000 | .441 | .448 | .458 | .418 | .439 | .191 | .211 | .227 | .207 | .209 | .207 |
| PCS2 | .441 | 1.000 | .465 | .472 | .451 | .434 | .188 | .206 | .194 | .223 | .218 | .221 |
| PCS3 | .448 | .465 | 1.000 | .410 | .453 | .418 | .184 | .214 | .201 | .219 | .178 | .199 |
| PCS4 | .458 | .472 | .410 | 1.000 | .406 | .409 | .211 | .224 | .197 | .202 | .234 | .231 |
| PCS5 | .418 | .451 | .453 | .406 | 1.000 | .406 | .191 | .226 | .182 | .192 | .203 | .193 |
| PCS6 | .439 | .434 | .418 | .409 | .406 | 1.000 | .191 | .285 | .162 | .179 | .241 | .149 |
| CIS1 | .191 | .188 | .184 | .211 | .191 | .191 | 1.000 | .392 | .393 | .387 | .410 | .378 |
| CIS2 | .211 | .206 | .214 | .224 | .226 | .285 | .392 | 1.000 | .421 | .415 | .431 | .369 |
| CIS3 | .227 | .194 | .201 | .197 | .182 | .162 | .393 | .421 | 1.000 | .383 | .434 | .389 |
| CIS4 | .207 | .223 | .219 | .202 | .192 | .179 | .387 | .415 | .383 | 1.000 | .403 | .387 |
| CIS5 | .209 | .218 | .178 | .234 | .203 | .241 | .410 | .431 | .434 | .403 | 1.000 | .413 |
| CIS6 | .207 | .221 | .199 | .231 | .193 | .149 | .378 | .369 | .389 | .387 | .413 | 1.000 |
