# Supplementary material for: Development and initial psychometric evaluation of a self-reported sustainable sport talent development literacy scale for physical education teachers
Source: Front Psychol. 2026 Aug 13;17:1902630. doi: 10.3389/fpsyg.2026.1902630 (PMC13518115; doi:10.3389/fpsyg.2026.1902630)
Supplement: Supplementary file 1 [file Data_Sheet_1.ZIP › Supplementary_Appendices_A-I/Appendix I Full lavaan output and reproducibility files.docx]

**Appendix I. CFA Verification and Reproducibility Materials**

**Purpose.** This appendix provides the materials used to verify the primary six-factor CFA, evaluate local fit, assess sensitivity to estimation choices and sample allocation, and reproduce the measurement-invariance analyses reported in the manuscript.

**I-1. Analysis environment and audit trail**

The primary CFA was rerun from a clean R session using the archived 800-case dataset. The original fixed-seed randomization procedure was retained, and the 400 cases assigned to the CFA subsample were reconstructed from the archived row order. The model was re-estimated without altering its specification, and the fit indices reported in the manuscript were reproduced.

*Table I-1. Software versions and primary CFA settings.*

| **Setting** | **Recorded value** |
| --- | --- |
| R | 4.6.1 (2026-06-24) |
| psych | 2.6.5 |
| lavaan | 0.6-21 |
| semTools | 0.5-9 |
| readxl | 1.5.0 |
| Primary estimator | WLSMV; DWLS estimation with mean- and variance-adjusted test statistics |
| Parameterization | delta |
| Ordered indicators | All 36 CFA items |
| Latent-variable scaling | std.lv = TRUE |
| Missing-data setting | listwise; no CFA item responses were missing |
| Fixed split | set.seed(20260531); N(EFA) = 400 and N(CFA) = 400 |
| Model restrictions | No correlated residuals, cross-loadings, equality constraints, or post hoc modifications |

**Complete R session information**

R version 4.6.1 (2026-06-24)

Platform: aarch64-apple-darwin25.4.0

Running under: macOS Tahoe 26.5.2

Matrix products: default

BLAS: /opt/homebrew/Cellar/openblas/0.3.33/lib/libopenblasp-r0.3.33.dylib

LAPACK: /opt/homebrew/Cellar/r/4.6.1/lib/R/lib/libRlapack.dylib; LAPACK version 3.12.1

locale:

[1] C.UTF-8/C.UTF-8/C.UTF-8/C/C.UTF-8/C.UTF-8

time zone: Asia/Shanghai

tzcode source: internal

attached base packages:

[1] stats	graphics grDevices utils	datasets methods	base

other attached packages:

[1] semTools_0.5-9 lavaan_0.6-21 psych_2.6.5	readxl_1.5.0

loaded via a namespace (and not attached):

[1] MASS_7.3-65	compiler_4.6.1	tools_4.6.1	parallel_4.6.1

[5] cellranger_1.1.0 nlme_3.1-169	mnormt_2.1.2	grid_4.6.1

[9] pbivnorm_0.6.0	stats4_4.6.1	lattice_0.22-9	quadprog_1.5-8

**Data release scope**

The public reproducibility archive contains the 36 core item responses for all 800 retained respondents because the full item set is required to reconstruct the fixed EFA/CFA split and the repeated-split sensitivity analysis. A separate file adds only the three grouping variables needed for measurement-invariance analyses. The original workbook, remaining demographic fields, the five-item sustainable PE practice intention measure, and platform or data-screening fields are not included in the public archive and remain available from the corresponding author on reasonable request. This scope matches the revised Data Availability Statement.

**I-2. Exact model syntax and estimation call**

The code below is a self-contained minimal reproduction using the archived 400-case CFA file. The six latent factors were freely correlated. Each item loaded on one prespecified factor, and no residual covariance or cross-loading was estimated.

if (is.na(parallel::detectCores())) {

unlockBinding("detectCores", asNamespace("parallel"))

assign("detectCores", function(logical = TRUE) 1L,

envir = asNamespace("parallel"))

lockBinding("detectCores", asNamespace("parallel"))

}

library(lavaan)

options(lavaan.ncpus = 1)

cfa_dat <- read.csv("cfa_subsample_item_data.csv", check.names = FALSE)

items <- c(paste0("HWB", 1:6), paste0("AMS", 1:6),

paste0("PEB", 1:6), paste0("PID", 1:6),

paste0("PCS", 1:6), paste0("CIS", 1:6))

model <- '

HWB =~ HWB1 + HWB2 + HWB3 + HWB4 + HWB5 + HWB6

AMS =~ AMS1 + AMS2 + AMS3 + AMS4 + AMS5 + AMS6

PEB =~ PEB1 + PEB2 + PEB3 + PEB4 + PEB5 + PEB6

PID =~ PID1 + PID2 + PID3 + PID4 + PID5 + PID6

PCS =~ PCS1 + PCS2 + PCS3 + PCS4 + PCS5 + PCS6

CIS =~ CIS1 + CIS2 + CIS3 + CIS4 + CIS5 + CIS6

'

fit <- cfa(model, data = cfa_dat, ordered = items,

estimator = "WLSMV", parameterization = "delta",

std.lv = TRUE, missing = "listwise",

parallel = "no")

**I-3. Reproduction of the primary six-factor CFA**

*Table I-2. Reproduced fit indices for the archived 400-case CFA subsample.*

| **Index** | **Result** |
| --- | --- |
| Scaled chi-square | 598.042 |
| Degrees of freedom | 579 |
| Scaled p value | .283 |
| Scaled CFI | .997 |
| Scaled TLI | .996 |
| Scaled RMSEA [90% CI] | .009 [.000, .019] |
| SRMR | .040 |
| Robust CFI | .985 |
| Robust TLI | .984 |
| Robust RMSEA [90% CI] | .018 [.000, .027] |
| Convergence | Normal convergence after 21 iterations |
| Estimation warnings | None |

**Complete standardized loading and residual-variance summary**

*Table I-3. Complete item loadings and standardized residual variances.*

| **Factor** | **Item** | **Loading** | **SE** | **z** | **95% CI** | **Residual variance** |
| --- | --- | --- | --- | --- | --- | --- |
| HWB | HWB1 | .595 | .048 | 12.47 | [.502, .689] | .646 |
| HWB | HWB2 | .701 | .043 | 16.28 | [.617, .786] | .508 |
| HWB | HWB3 | .682 | .043 | 15.73 | [.597, .767] | .535 |
| HWB | HWB4 | .540 | .052 | 10.45 | [.439, .642] | .708 |
| HWB | HWB5 | .606 | .048 | 12.62 | [.511, .700] | .633 |
| HWB | HWB6 | .632 | .050 | 12.72 | [.534, .729] | .601 |
| AMS | AMS1 | .669 | .041 | 16.14 | [.588, .750] | .553 |
| AMS | AMS2 | .698 | .037 | 18.76 | [.625, .770] | .513 |
| AMS | AMS3 | .644 | .041 | 15.57 | [.563, .725] | .585 |
| AMS | AMS4 | .653 | .042 | 15.56 | [.570, .735] | .574 |
| AMS | AMS5 | .697 | .039 | 17.95 | [.621, .773] | .514 |
| AMS | AMS6 | .683 | .037 | 18.42 | [.610, .756] | .533 |
| PEB | PEB1 | .643 | .043 | 14.94 | [.558, .727] | .587 |
| PEB | PEB2 | .749 | .039 | 19.02 | [.672, .827] | .438 |
| PEB | PEB3 | .626 | .044 | 14.11 | [.539, .713] | .608 |
| PEB | PEB4 | .713 | .040 | 17.97 | [.635, .790] | .492 |
| PEB | PEB5 | .580 | .048 | 12.11 | [.486, .674] | .663 |
| PEB | PEB6 | .636 | .045 | 14.26 | [.549, .723] | .595 |
| PID | PID1 | .651 | .045 | 14.55 | [.563, .738] | .577 |
| PID | PID2 | .653 | .045 | 14.38 | [.564, .742] | .574 |
| PID | PID3 | .600 | .048 | 12.42 | [.506, .695] | .640 |
| PID | PID4 | .708 | .041 | 17.42 | [.628, .788] | .499 |
| PID | PID5 | .688 | .043 | 16.07 | [.604, .772] | .526 |
| PID | PID6 | .681 | .043 | 15.77 | [.596, .765] | .537 |
| PCS | PCS1 | .636 | .040 | 15.86 | [.558, .715] | .595 |
| PCS | PCS2 | .705 | .041 | 17.32 | [.625, .784] | .503 |
| PCS | PCS3 | .688 | .041 | 16.82 | [.608, .768] | .527 |
| PCS | PCS4 | .718 | .036 | 19.86 | [.647, .788] | .485 |
| PCS | PCS5 | .679 | .043 | 15.77 | [.595, .764] | .539 |
| PCS | PCS6 | .635 | .042 | 15.18 | [.553, .717] | .597 |
| CIS | CIS1 | .663 | .044 | 14.93 | [.576, .750] | .560 |
| CIS | CIS2 | .699 | .040 | 17.63 | [.622, .777] | .511 |
| CIS | CIS3 | .661 | .042 | 15.82 | [.580, .743] | .562 |
| CIS | CIS4 | .699 | .039 | 17.92 | [.623, .776] | .511 |
| CIS | CIS5 | .697 | .037 | 18.93 | [.625, .769] | .515 |
| CIS | CIS6 | .608 | .047 | 12.84 | [.515, .700] | .631 |

**Latent factor correlations**

*Table I-4. Complete latent factor correlation matrix.*

| **Factor** | **HWB** | **AMS** | **PEB** | **PID** | **PCS** | **CIS** |
| --- | --- | --- | --- | --- | --- | --- |
| HWB | 1.000 | 0.535 | 0.528 | 0.386 | 0.432 | 0.431 |
| AMS | 0.535 | 1.000 | 0.519 | 0.434 | 0.572 | 0.450 |
| PEB | 0.528 | 0.519 | 1.000 | 0.414 | 0.369 | 0.463 |
| PID | 0.386 | 0.434 | 0.414 | 1.000 | 0.423 | 0.494 |
| PCS | 0.432 | 0.572 | 0.369 | 0.423 | 1.000 | 0.423 |
| CIS | 0.431 | 0.450 | 0.463 | 0.494 | 0.423 | 1.000 |

**Item thresholds**

*Table I-5. Complete thresholds for the 36 ordered indicators.*

| **Item** | **t1** | **t2** | **t3** | **t4** |
| --- | --- | --- | --- | --- |
| AMS1 | -1.812 | −.915 | −.006 | .833 |
| AMS2 | -1.845 | −.815 | .013 | .714 |
| AMS3 | -1.845 | −.964 | .019 | .628 |
| AMS4 | -1.845 | −.906 | −.056 | .747 |
| AMS5 | -1.695 | −.878 | −.044 | .722 |
| AMS6 | -1.751 | −.887 | .013 | .731 |
| CIS1 | -1.598 | −.860 | .082 | .764 |
| CIS2 | -1.670 | −.833 | .075 | .925 |
| CIS3 | -1.960 | −.824 | −.050 | .860 |
| CIS4 | -1.751 | −.833 | .025 | .789 |
| CIS5 | -1.695 | −.789 | .031 | .860 |
| CIS6 | -1.695 | −.789 | −.006 | .851 |
| HWB1 | -1.881 | −.806 | .082 | .878 |
| HWB2 | -1.598 | −.815 | .056 | .925 |
| HWB3 | -1.812 | −.833 | .157 | .954 |
| HWB4 | -1.645 | −.789 | .063 | .896 |
| HWB5 | -1.845 | −.860 | .025 | .842 |
| HWB6 | -1.722 | −.842 | .044 | .896 |
| PCS1 | -1.555 | −.690 | .126 | .954 |
| PCS2 | -1.751 | −.944 | −.063 | .798 |
| PCS3 | -1.695 | −.772 | .088 | .833 |
| PCS4 | -1.670 | −.806 | .063 | .798 |
| PCS5 | -1.670 | −.798 | .006 | .869 |
| PCS6 | -1.780 | −.896 | .107 | .906 |
| PEB1 | -1.722 | −.806 | .082 | .842 |
| PEB2 | -1.695 | −.722 | .119 | .925 |
| PEB3 | -1.751 | −.789 | .107 | .925 |
| PEB4 | -1.670 | −.824 | .126 | .869 |
| PEB5 | -1.670 | −.842 | .075 | .798 |
| PEB6 | -1.514 | −.706 | .082 | .896 |
| PID1 | -1.598 | −.747 | .107 | .906 |
| PID2 | -1.695 | −.789 | .044 | .896 |
| PID3 | -1.722 | −.764 | .056 | .869 |
| PID4 | -1.555 | −.714 | .170 | .964 |
| PID5 | -1.514 | −.739 | .025 | .860 |
| PID6 | -1.645 | −.764 | .044 | .824 |

**I-4. Modification indices and local dependence**

Modification indices were inspected as diagnostics only. No parameter was freed after inspection. The complete output contains 810 fixed parameters and is supplied in all_modification_indices.csv. Table I-6 reports every modification index at or above 3.84, not a post hoc model-selection subset.

*Table I-6. All modification indices of 3.84 or larger.*

| **lhs** | **op** | **rhs** | **MI** | **EPC** | **Std. EPC** |
| --- | --- | --- | --- | --- | --- |
| PEB | =~ | PID6 | 13.80 | .161 | .161 |
| PCS | =~ | AMS5 | 10.71 | .186 | .186 |
| PCS | =~ | PID2 | 10.64 | −.141 | −.141 |
| HWB4 | ~~ | PID6 | 8.15 | .143 | .232 |
| HWB | =~ | PID2 | 7.75 | −.121 | −.121 |
| HWB | =~ | PCS1 | 7.34 | −.125 | −.125 |
| HWB4 | ~~ | CIS5 | 7.05 | −.145 | −.240 |
| HWB4 | ~~ | PID3 | 6.85 | .138 | .205 |
| HWB | =~ | PEB5 | 6.30 | −.139 | −.139 |
| HWB1 | ~~ | PEB5 | 6.02 | −.139 | −.212 |
| PEB6 | ~~ | PID1 | 5.82 | −.137 | −.234 |
| PID | =~ | CIS5 | 5.75 | −.126 | −.126 |
| HWB2 | ~~ | PEB6 | 5.62 | .122 | .222 |
| PEB | =~ | PCS1 | 5.60 | −.097 | −.097 |
| PID | =~ | AMS1 | 5.55 | .107 | .107 |
| HWB4 | ~~ | CIS2 | 5.24 | −.129 | −.214 |
| HWB1 | ~~ | PID2 | 5.09 | −.126 | −.207 |
| PID | =~ | PEB2 | 4.98 | .105 | .105 |
| PEB1 | ~~ | PID1 | 4.97 | −.122 | −.210 |
| AMS | =~ | HWB3 | 4.81 | .121 | .121 |
| PCS6 | ~~ | CIS2 | 4.60 | .109 | .198 |
| PCS | =~ | AMS2 | 4.60 | −.128 | −.128 |
| PID | =~ | PEB6 | 4.55 | −.096 | −.096 |
| PID | =~ | CIS1 | 4.40 | .110 | .110 |
| PID5 | ~~ | PID6 | 4.30 | −.112 | −.211 |
| PEB3 | ~~ | PID6 | 4.24 | .107 | .188 |
| PCS | =~ | PEB2 | 4.24 | .090 | .090 |
| PEB | =~ | PID1 | 4.22 | −.090 | −.090 |
| PID2 | ~~ | PCS2 | 4.15 | −.115 | −.214 |
| CIS | =~ | HWB4 | 4.14 | −.093 | −.093 |
| PID | =~ | PCS3 | 4.08 | .090 | .090 |
| CIS | =~ | PEB6 | 3.99 | −.098 | −.098 |
| HWB | =~ | PEB6 | 3.97 | .111 | .111 |
| AMS1 | ~~ | PID4 | 3.95 | .100 | .191 |
| AMS | =~ | PEB2 | 3.95 | .106 | .106 |
| AMS | =~ | PCS1 | 3.91 | −.107 | −.107 |

*Table I-7. Twenty largest local residual dependencies by absolute residual correlation.*

| **Item pair** | **Residual correlation** | **Normalized residual z** |
| --- | --- | --- |
| CIS5–HWB4 | −.138 | -3.32 |
| PID6–HWB4 | .136 | 3.44 |
| PID3–HWB4 | .133 | 3.06 |
| PEB5–HWB1 | −.133 | -3.17 |
| PID1–PEB6 | −.132 | -3.01 |
| CIS2–HWB4 | −.124 | -2.88 |
| PID2–HWB1 | −.121 | -2.84 |
| PID1–PEB1 | −.117 | -2.89 |
| PEB6–HWB2 | .112 | 2.89 |
| PCS2–PID2 | −.109 | -2.70 |
| CIS2–PCS6 | .103 | 2.72 |
| PID6–PEB3 | .102 | 2.48 |
| PCS5–HWB1 | .096 | 2.17 |
| PEB1–AMS6 | −.095 | -2.47 |
| PID4–AMS1 | .094 | 2.49 |
| PCS2–HWB4 | −.093 | -2.34 |
| PCS3–PID1 | .092 | 2.36 |
| PCS1–PEB4 | −.091 | -2.09 |
| CIS6–AMS1 | −.088 | -1.97 |
| PID6–PID5 | −.087 | -2.59 |

The largest absolute residual correlation was .138 between CIS5 and HWB4. The largest normalized residuals were 3.44 for PID6–HWB4 and −3.32 for CIS5–HWB4. The largest residual-covariance modification index was 8.15 for HWB4–PID6 (standardized EPC = .232). The largest within-factor absolute residual correlation was .087 for PID5–PID6.

*Table I-8. Content-similar item pairs examined for local dependence.*

| **Item pair** | **Residual correlation** | **Normalized residual z** |
| --- | --- | --- |
| HWB1–HWB6 | .047 | 1.23 |
| HWB2–HWB4 | −.006 | −0.14 |
| PEB1–PEB2 | −.015 | −0.43 |
| PEB4–PEB6 | .013 | 0.38 |
| PID1–PID2 | .060 | 1.64 |
| AMS4–AMS5 | .011 | 0.34 |
| CIS3–CIS4 | −.055 | −1.62 |

**I-5. Estimator, parameterization, competing-model, and split sensitivity**

*Table I-9. Alternative estimators, parameterizations, and competing models.*

| **Specification** | **Scaled χ²** | **df** | **Robust CFI** | **Robust TLI** | **Robust RMSEA** | **SRMR** |
| --- | --- | --- | --- | --- | --- | --- |
| Six-factor WLSMV, delta (CFA split) | 598.042 | 579 | .985 | .984 | .018 | .040 |
| Six-factor WLSMV, theta (CFA split) | 598.042 | 579 | .985 | .984 | .018 | .040 |
| Six-factor ULSMV, theta (CFA split) | 596.192 | 579 | .985 | .983 | .018 | .040 |
| One-factor WLSMV, delta (CFA split) | 2220.388 | 594 | .604 | .580 | .090 | .093 |
| Six-factor WLSMV, delta (full sample; check only) | 608.171 | 579 | .995 | .994 | .010 | .028 |

The theta-parameterized WLSMV model reproduced the primary rounded indices. ULSMV with theta parameterization gave nearly identical results. The revised one-factor comparison uses the same archived 400-case CFA subsample as the primary model and fit substantially worse. The full-sample row is shown only to demonstrate that the manuscript's primary CFA values were not taken from the full sample.

*Table I-10. Complete results for 20 additional random 400-case splits.*

| **Seed** | **n** | **Robust CFI** | **Robust TLI** | **Robust RMSEA** | **SRMR** | **Scaled CFI** | **Scaled TLI** | **Scaled RMSEA** |
| --- | --- | --- | --- | --- | --- | --- | --- | --- |
| 101 | 400 | .984 | .983 | .018 | .040 | .997 | .997 | .008 |
| 202 | 400 | .975 | .973 | .023 | .040 | .991 | .990 | .015 |
| 303 | 400 | .988 | .986 | .017 | .039 | .996 | .996 | .010 |
| 404 | 400 | 1.000 | 1.000 | .002 | .038 | 1.000 | 1.002 | .000 |
| 505 | 400 | .972 | .969 | .025 | .040 | .993 | .992 | .015 |
| 606 | 400 | .998 | .998 | .006 | .038 | 1.000 | 1.002 | .000 |
| 707 | 400 | .989 | .988 | .015 | .040 | .997 | .996 | .009 |
| 808 | 400 | .992 | .992 | .013 | .037 | 1.000 | 1.001 | .000 |
| 909 | 400 | .998 | .997 | .007 | .039 | 1.000 | 1.000 | .001 |
| 1010 | 400 | .989 | .988 | .016 | .038 | .997 | .996 | .010 |
| 1111 | 400 | .992 | .991 | .013 | .039 | .999 | .998 | .006 |
| 1212 | 400 | .973 | .970 | .024 | .042 | .990 | .989 | .016 |
| 1313 | 400 | .978 | .976 | .022 | .040 | .994 | .994 | .012 |
| 1414 | 400 | .984 | .983 | .018 | .041 | .990 | .989 | .016 |
| 1515 | 400 | .972 | .970 | .025 | .041 | .993 | .992 | .014 |
| 1616 | 400 | .976 | .973 | .023 | .040 | .996 | .995 | .010 |
| 1717 | 400 | .977 | .975 | .021 | .042 | .991 | .990 | .014 |
| 1818 | 400 | .991 | .990 | .014 | .039 | .999 | .998 | .006 |
| 1919 | 400 | .988 | .987 | .016 | .038 | 1.000 | 1.002 | .000 |
| 2020 | 400 | .973 | .971 | .024 | .038 | .998 | .998 | .007 |

*Table I-11. Summary of the 20 repeated splits.*

| **Index** | **Median** | **Minimum** | **Maximum** |
| --- | --- | --- | --- |
| Robust CFI | .986 | .972 | 1.000 |
| Robust TLI | .985 | .969 | 1.000 |
| Robust RMSEA | .017 | .002 | .025 |
| SRMR | .039 | .037 | .042 |

**I-6. Why favorable global fit can coexist with AVE below .50**

Global fit and AVE evaluate different properties of a measurement model. CFI, TLI, RMSEA, and SRMR assess how closely the specified factor structure reproduces the observed polychoric correlations and thresholds. AVE is the mean squared standardized loading and therefore quantifies the proportion of item variance attributable, on average, to the intended factor. In this analysis, standardized loadings ranged from .540 to .749, yielding AVE values from .395 to .459 and leaving substantial item-specific variance. That variance does not necessarily worsen global fit when it is largely uncorrelated and the factor pattern reproduces the observed associations. The small local residual correlations and stable sensitivity results are consistent with this explanation. The favorable global fit does not compensate for the limited convergent-validity evidence indicated by AVE below .50.

**I-7. Measurement-invariance syntax and group definitions**

These measurement-invariance analyses reused the full retained sample after the fixed EFA/CFA split. They should therefore be treated as secondary full-sample analyses rather than independent confirmatory tests. The ordered-categorical sequence matched that reported in the manuscript: configural, threshold, and threshold-plus-loading models. Equality constraints were introduced sequentially, with both thresholds and loadings constrained in the final model. The school-stage comparison excluded teachers in nine-year/complete secondary schools and other settings.

Identification followed lavaan 0.6-21's multiple-group defaults for ordered indicators with delta parameterization and std.lv = TRUE. In the configural model, item thresholds and loadings were estimated separately by group; item-response intercepts were fixed to zero, item-response scaling factors were fixed to one, and latent means and variances were fixed to zero and one, respectively, in each group. In the threshold-invariance model, all four thresholds per item were constrained equal across groups. lavaan retained zero item intercepts and unit scaling factors in the reference group while freeing the corresponding intercepts and scaling factors in the comparison group. In the threshold-plus-loading model, factor loadings were also constrained equal; latent variances were then fixed to one in the reference group and estimated in the comparison group. Latent means remained fixed to zero because no latent-mean comparisons were conducted. No intercept, residual-variance, latent-mean, or latent-covariance equality constraints were added.

The reference groups were male teachers for gender, teachers without a school team coaching role for coaching role, and primary-school teachers for school stage. The accompanying identification-settings file records these choices and the exact constraints applied at each step.

*Table I-12. Measurement-invariance group definitions and sample sizes.*

| **Comparison** | **Group** | **n** |
| --- | --- | --- |
| Gender | Male | 477 |
| Gender | Female | 310 |
| School team coaching role | No | 479 |
| School team coaching role | Yes | 321 |
| School stage | Primary | 259 |
| School stage | Secondary | 438 |

*Table I-13. Ordered-categorical measurement-invariance models.*

| **Comparison** | **Model** | **Scaled χ²** | **df** | **CFI** | **TLI** | **RMSEA** | **SRMR** | **ΔCFI** | **ΔRMSEA** | **ΔSRMR** |
| --- | --- | --- | --- | --- | --- | --- | --- | --- | --- | --- |
| Gender | Configural | 1191.842 | 1158 | .997 | .997 | .009 | .039 | — | — | — |
| Gender | Threshold | 1266.384 | 1230 | .997 | .997 | .009 | .039 | .000 | .000 | .000 |
| Gender | Threshold + loading | 1294.069 | 1260 | .997 | .997 | .008 | .040 | .000 | .000 | .001 |
| School team coaching role | Configural | 1239.826 | 1158 | .993 | .993 | .013 | .041 | — | — | — |
| School team coaching role | Threshold | 1310.131 | 1230 | .994 | .993 | .013 | .041 | .000 | −.001 | .000 |
| School team coaching role | Threshold + loading | 1319.871 | 1260 | .995 | .995 | .011 | .041 | .002 | −.002 | .000 |
| School stage | Configural | 1210.927 | 1158 | .995 | .994 | .011 | .042 | — | — | — |
| School stage | Threshold | 1280.700 | 1230 | .995 | .995 | .011 | .042 | .000 | −.001 | .000 |
| School stage | Threshold + loading | 1293.543 | 1260 | .997 | .997 | .009 | .042 | .002 | −.002 | .000 |

Note. Changes in fit indices were calculated relative to the preceding model. The results were consistent with configural, threshold, and threshold-plus-loading invariance across the three grouping variables. This conclusion is limited to the parameters tested and does not establish full measurement equivalence, conventional scalar invariance, latent-mean comparability, or unbiased observed-score comparisons. Latent means were fixed at zero and were not compared.

All nine invariance models converged normally. No estimation warnings were recorded.

**I-8. Reproducibility file map**

*Table I-14. Key files in the accompanying machine-readable archive.*

| **File** | **Contents** | **SHA-256 (first 16 characters)** |
| --- | --- | --- |
| cfa_editor_verification.R | Executable primary rerun and sensitivity analyses | 5ce8c85035e62a25 |
| six_factor_model_syntax.txt | Complete six-factor measurement syntax | 7e6133d1e001972a |
| full_core36_item_data.csv | De-identified 36-item data for all 800 retained respondents | 694379b445fef2df |
| split_manifest_seed_20260531.csv | Original row, respondent ID, and EFA/CFA assignment | d7e9c8ee14d58c53 |
| cfa_subsample_item_data.csv | De-identified 400-case CFA input data | 5172d3186aa67a1c |
| polychoric_correlation_matrix_cfa_subsample.csv | Complete CFA polychoric correlation matrix | d03c24bf29721859 |
| complete_lavaan_output.txt | Complete primary CFA printed output | a41451e40faa014f |
| complete_standardized_solution.csv | Complete standardized CFA solution | 8dabbc20c4e3d2ab |
| residual_and_variance_estimates.csv | All residual and variance parameters | fb192959cf913806 |
| latent_factor_correlations.csv | Complete latent correlation matrix | 1b88ab8c66cbabf6 |
| item_thresholds.csv | All 144 item thresholds | e8ded98c9071c6b0 |
| all_modification_indices.csv | All 810 modification indices | 433cbcebb751c554 |
| residual_correlation_matrix.csv | Complete correlation-scale residual matrix | 4c7494f10174776f |
| standardized_residual_correlation_z_matrix.csv | Complete normalized residual matrix | e09766d884397983 |
| ranked_residual_correlations.csv | Residual correlations ranked by absolute magnitude | f70b9f1d6ad79552 |
| ranked_standardized_residual_correlations.csv | Normalized residuals ranked by absolute magnitude | fafea46dd48b1262 |
| estimator_parameterization_and_model_sensitivity.csv | Theta, ULSMV, one-factor, and full-sample checks | de2b203d63a51e6a |
| repeated_split_fit_indices_20_splits.csv | Fit indices for all 20 repeated splits | 8bbae415d0fd89ea |
| measurement_invariance_verification.R | Invariance code aligned with the manuscript | 0bd643e7a8b0bb4b |
| measurement_invariance_data.csv | De-identified invariance item and grouping data | 38174b89ea48deaf |
| measurement_invariance_corrected_results.csv | Complete measurement-invariance results | bbe5504d9f7f2f3b |
| measurement_invariance_identification_settings.txt | Exact invariance identification constraints and reference groups | 1d47908442b35400 |
| sessionInfo.txt | Primary R and package session record | 633ee28d79d2fbe2 |

**Annex I-A. Complete primary lavaan output**

The following output is the complete printed result from the primary model rerun in a clean R session. It contains fit statistics, the standardized solution, item thresholds, residual variances, and latent factor correlations.

lavaan 0.6-21 ended normally after 21 iterations

Estimator	DWLS

Optimization method	NLMINB

Number of model parameters	195

Number of observations	400

Model Test User Model:

Standard	Scaled

Test Statistic	397.983	598.042

Degrees of freedom	579	579

P-value (Unknown)	NA	0.283

Scaling correction factor	1.147

Shift parameter	251.039

simple second-order correction

Model Test Baseline Model:

Test statistic	17059.941	6510.185

Degrees of freedom	630	630

P-value	NA	0.000

Scaling correction factor	2.794

User Model versus Baseline Model:

Comparative Fit Index (CFI)	1.000	0.997

Tucker-Lewis Index (TLI)	1.012	0.996

Robust Comparative Fit Index (CFI)	0.985

Robust Tucker-Lewis Index (TLI)	0.984

Root Mean Square Error of Approximation:

RMSEA	0.000	0.009

90 Percent confidence interval - lower	0.000	0.000

90 Percent confidence interval - upper	0.000	0.019

P-value H_0: RMSEA <= 0.050	1.000	1.000

P-value H_0: RMSEA >= 0.080	0.000	0.000

Robust RMSEA	0.018

90 Percent confidence interval - lower	0.000

90 Percent confidence interval - upper	0.027

P-value H_0: Robust RMSEA <= 0.050	1.000

P-value H_0: Robust RMSEA >= 0.080	0.000

Standardized Root Mean Square Residual:

SRMR	0.040	0.040

Parameter Estimates:

Parameterization	Delta

Standard errors	Robust.sem

Information	Expected

Information saturated (h1) model	Unstructured

Latent Variables:

Estimate Std.Err z-value P(>|z|)	Std.lv Std.all

HWB =~

HWB1	0.595	0.048	12.472	0.000	0.595	0.595

HWB2	0.701	0.043	16.278	0.000	0.701	0.701

HWB3	0.682	0.043	15.725	0.000	0.682	0.682

HWB4	0.540	0.052	10.448	0.000	0.540	0.540

HWB5	0.606	0.048	12.616	0.000	0.606	0.606

HWB6	0.632	0.050	12.720	0.000	0.632	0.632

AMS =~

AMS1	0.669	0.041	16.141	0.000	0.669	0.669

AMS2	0.698	0.037	18.759	0.000	0.698	0.698

AMS3	0.644	0.041	15.570	0.000	0.644	0.644

AMS4	0.653	0.042	15.560	0.000	0.653	0.653

AMS5	0.697	0.039	17.949	0.000	0.697	0.697

AMS6	0.683	0.037	18.420	0.000	0.683	0.683

PEB =~

PEB1	0.643	0.043	14.939	0.000	0.643	0.643

PEB2	0.749	0.039	19.022	0.000	0.749	0.749

PEB3	0.626	0.044	14.109	0.000	0.626	0.626

PEB4	0.713	0.040	17.970	0.000	0.713	0.713

PEB5	0.580	0.048	12.109	0.000	0.580	0.580

PEB6	0.636	0.045	14.257	0.000	0.636	0.636

PID =~

PID1	0.651	0.045	14.551	0.000	0.651	0.651

PID2	0.653	0.045	14.376	0.000	0.653	0.653

PID3	0.600	0.048	12.419	0.000	0.600	0.600

PID4	0.708	0.041	17.418	0.000	0.708	0.708

PID5	0.688	0.043	16.069	0.000	0.688	0.688

PID6	0.681	0.043	15.772	0.000	0.681	0.681

PCS =~

PCS1	0.636	0.040	15.861	0.000	0.636	0.636

PCS2	0.705	0.041	17.324	0.000	0.705	0.705

PCS3	0.688	0.041	16.825	0.000	0.688	0.688

PCS4	0.718	0.036	19.855	0.000	0.718	0.718

PCS5	0.679	0.043	15.772	0.000	0.679	0.679

PCS6	0.635	0.042	15.180	0.000	0.635	0.635

CIS =~

CIS1	0.663	0.044	14.933	0.000	0.663	0.663

CIS2	0.699	0.040	17.630	0.000	0.699	0.699

CIS3	0.661	0.042	15.821	0.000	0.661	0.661

CIS4	0.699	0.039	17.922	0.000	0.699	0.699

CIS5	0.697	0.037	18.931	0.000	0.697	0.697

CIS6	0.608	0.047	12.837	0.000	0.608	0.608

Covariances:

Estimate Std.Err z-value P(>|z|)	Std.lv Std.all

HWB ~~

AMS	0.535	0.047	11.396	0.000	0.535	0.535

PEB	0.528	0.049	10.676	0.000	0.528	0.528

PID	0.386	0.054	7.140	0.000	0.386	0.386

PCS	0.432	0.053	8.214	0.000	0.432	0.432

CIS	0.431	0.052	8.280	0.000	0.431	0.431

AMS ~~

PEB	0.519	0.050	10.437	0.000	0.519	0.519

PID	0.434	0.051	8.466	0.000	0.434	0.434

PCS	0.572	0.045	12.832	0.000	0.572	0.572

CIS	0.450	0.052	8.726	0.000	0.450	0.450

PEB ~~

PID	0.414	0.054	7.729	0.000	0.414	0.414

PCS	0.369	0.053	6.907	0.000	0.369	0.369

CIS	0.463	0.051	9.004	0.000	0.463	0.463

PID ~~

PCS	0.423	0.052	8.095	0.000	0.423	0.423

CIS	0.494	0.050	9.917	0.000	0.494	0.494

PCS ~~

CIS	0.423	0.051	8.269	0.000	0.423	0.423

Thresholds:

Estimate Std.Err z-value P(>|z|)	Std.lv Std.all

HWB1|t1	-1.881	0.126 -14.985	0.000	-1.881	-1.881

HWB1|t2	-0.806	0.071 -11.398	0.000	-0.806	-0.806

HWB1|t3	0.082	0.063	1.298	0.194	0.082	0.082

HWB1|t4	0.878	0.072	12.130	0.000	0.878	0.878

HWB2|t1	-1.598	0.103 -15.577	0.000	-1.598	-1.598

HWB2|t2	-0.815	0.071 -11.490	0.000	-0.815	-0.815

HWB2|t3	0.056	0.063	0.899	0.369	0.056	0.056

HWB2|t4	0.925	0.074	12.577	0.000	0.925	0.925

HWB3|t1	-1.812	0.119 -15.217	0.000	-1.812	-1.812

HWB3|t2	-0.833	0.071 -11.675	0.000	-0.833	-0.833

HWB3|t3	0.157	0.063	2.496	0.013	0.157	0.157

HWB3|t4	0.954	0.074	12.840	0.000	0.954	0.954

HWB4|t1	-1.645	0.106 -15.548	0.000	-1.645	-1.645

HWB4|t2	-0.789	0.070 -11.212	0.000	-0.789	-0.789

HWB4|t3	0.063	0.063	0.999	0.318	0.063	0.063

HWB4|t4	0.896	0.073	12.310	0.000	0.896	0.896

HWB5|t1	-1.845	0.122 -15.111	0.000	-1.845	-1.845

HWB5|t2	-0.860	0.072 -11.949	0.000	-0.860	-0.860

HWB5|t3	0.025	0.063	0.399	0.690	0.025	0.025

HWB5|t4	0.842	0.072	11.766	0.000	0.842	0.842

HWB6|t1	-1.722	0.112 -15.437	0.000	-1.722	-1.722

HWB6|t2	-0.842	0.072 -11.766	0.000	-0.842	-0.842

HWB6|t3	0.044	0.063	0.699	0.484	0.044	0.044

HWB6|t4	0.896	0.073	12.310	0.000	0.896	0.896

AMS1|t1	-1.812	0.119 -15.217	0.000	-1.812	-1.812

AMS1|t2	-0.915	0.073 -12.488	0.000	-0.915	-0.915

AMS1|t3	-0.006	0.063	-0.100	0.920	-0.006	-0.006

AMS1|t4	0.833	0.071	11.675	0.000	0.833	0.833

AMS2|t1	-1.845	0.122 -15.111	0.000	-1.845	-1.845

AMS2|t2	-0.815	0.071 -11.490	0.000	-0.815	-0.815

AMS2|t3	0.013	0.063	0.200	0.842	0.013	0.013

AMS2|t4	0.714	0.069	10.365	0.000	0.714	0.714

AMS3|t1	-1.845	0.122 -15.111	0.000	-1.845	-1.845

AMS3|t2	-0.964	0.075 -12.927	0.000	-0.964	-0.964

AMS3|t3	0.019	0.063	0.300	0.764	0.019	0.019

AMS3|t4	0.628	0.067	9.310	0.000	0.628	0.628

AMS4|t1	-1.845	0.122 -15.111	0.000	-1.845	-1.845

AMS4|t2	-0.906	0.073 -12.399	0.000	-0.906	-0.906

AMS4|t3	-0.056	0.063	-0.899	0.369	-0.056	-0.056

AMS4|t4	0.747	0.070	10.743	0.000	0.747	0.747

AMS5|t1	-1.695	0.109 -15.485	0.000	-1.695	-1.695

AMS5|t2	-0.878	0.072 -12.130	0.000	-0.878	-0.878

AMS5|t3	-0.044	0.063	-0.699	0.484	-0.044	-0.044

AMS5|t4	0.722	0.069	10.460	0.000	0.722	0.722

AMS6|t1	-1.751	0.114 -15.378	0.000	-1.751	-1.751

AMS6|t2	-0.887	0.073 -12.220	0.000	-0.887	-0.887

AMS6|t3	0.013	0.063	0.200	0.842	0.013	0.013

AMS6|t4	0.731	0.069	10.554	0.000	0.731	0.731

PEB1|t1	-1.722	0.112 -15.437	0.000	-1.722	-1.722

PEB1|t2	-0.806	0.071 -11.398	0.000	-0.806	-0.806

PEB1|t3	0.082	0.063	1.298	0.194	0.082	0.082

PEB1|t4	0.842	0.072	11.766	0.000	0.842	0.842

PEB2|t1	-1.695	0.109 -15.485	0.000	-1.695	-1.695

PEB2|t2	-0.722	0.069 -10.460	0.000	-0.722	-0.722

PEB2|t3	0.119	0.063	1.897	0.058	0.119	0.119

PEB2|t4	0.925	0.074	12.577	0.000	0.925	0.925

PEB3|t1	-1.751	0.114 -15.378	0.000	-1.751	-1.751

PEB3|t2	-0.789	0.070 -11.212	0.000	-0.789	-0.789

PEB3|t3	0.107	0.063	1.698	0.090	0.107	0.107

PEB3|t4	0.925	0.074	12.577	0.000	0.925	0.925

PEB4|t1	-1.670	0.108 -15.521	0.000	-1.670	-1.670

PEB4|t2	-0.824	0.071 -11.583	0.000	-0.824	-0.824

PEB4|t3	0.126	0.063	1.997	0.046	0.126	0.126

PEB4|t4	0.869	0.072	12.040	0.000	0.869	0.869

PEB5|t1	-1.670	0.108 -15.521	0.000	-1.670	-1.670

PEB5|t2	-0.842	0.072 -11.766	0.000	-0.842	-0.842

PEB5|t3	0.075	0.063	1.198	0.231	0.075	0.075

PEB5|t4	0.798	0.071	11.305	0.000	0.798	0.798

PEB6|t1	-1.514	0.097 -15.555	0.000	-1.514	-1.514

PEB6|t2	-0.706	0.069 -10.269	0.000	-0.706	-0.706

PEB6|t3	0.082	0.063	1.298	0.194	0.082	0.082

PEB6|t4	0.896	0.073	12.310	0.000	0.896	0.896

PID1|t1	-1.598	0.103 -15.577	0.000	-1.598	-1.598

PID1|t2	-0.747	0.070 -10.743	0.000	-0.747	-0.747

PID1|t3	0.107	0.063	1.698	0.090	0.107	0.107

PID1|t4	0.906	0.073	12.399	0.000	0.906	0.906

PID2|t1	-1.695	0.109 -15.485	0.000	-1.695	-1.695

PID2|t2	-0.789	0.070 -11.212	0.000	-0.789	-0.789

PID2|t3	0.044	0.063	0.699	0.484	0.044	0.044

PID2|t4	0.896	0.073	12.310	0.000	0.896	0.896

PID3|t1	-1.722	0.112 -15.437	0.000	-1.722	-1.722

PID3|t2	-0.764	0.070 -10.931	0.000	-0.764	-0.764

PID3|t3	0.056	0.063	0.899	0.369	0.056	0.056

PID3|t4	0.869	0.072	12.040	0.000	0.869	0.869

PID4|t1	-1.555	0.100 -15.578	0.000	-1.555	-1.555

PID4|t2	-0.714	0.069 -10.365	0.000	-0.714	-0.714

PID4|t3	0.170	0.063	2.695	0.007	0.170	0.170

PID4|t4	0.964	0.075	12.927	0.000	0.964	0.964

PID5|t1	-1.514	0.097 -15.555	0.000	-1.514	-1.514

PID5|t2	-0.739	0.069 -10.649	0.000	-0.739	-0.739

PID5|t3	0.025	0.063	0.399	0.690	0.025	0.025

PID5|t4	0.860	0.072	11.949	0.000	0.860	0.860

PID6|t1	-1.645	0.106 -15.548	0.000	-1.645	-1.645

PID6|t2	-0.764	0.070 -10.931	0.000	-0.764	-0.764

PID6|t3	0.044	0.063	0.699	0.484	0.044	0.044

PID6|t4	0.824	0.071	11.583	0.000	0.824	0.824

PCS1|t1	-1.555	0.100 -15.578	0.000	-1.555	-1.555

PCS1|t2	-0.690	0.068 -10.079	0.000	-0.690	-0.690

PCS1|t3	0.126	0.063	1.997	0.046	0.126	0.126

PCS1|t4	0.954	0.074	12.840	0.000	0.954	0.954

PCS2|t1	-1.751	0.114 -15.378	0.000	-1.751	-1.751

PCS2|t2	-0.944	0.074 -12.753	0.000	-0.944	-0.944

PCS2|t3	-0.063	0.063	-0.999	0.318	-0.063	-0.063

PCS2|t4	0.798	0.071	11.305	0.000	0.798	0.798

PCS3|t1	-1.695	0.109 -15.485	0.000	-1.695	-1.695

PCS3|t2	-0.772	0.070 -11.025	0.000	-0.772	-0.772

PCS3|t3	0.088	0.063	1.398	0.162	0.088	0.088

PCS3|t4	0.833	0.071	11.675	0.000	0.833	0.833

PCS4|t1	-1.670	0.108 -15.521	0.000	-1.670	-1.670

PCS4|t2	-0.806	0.071 -11.398	0.000	-0.806	-0.806

PCS4|t3	0.063	0.063	0.999	0.318	0.063	0.063

PCS4|t4	0.798	0.071	11.305	0.000	0.798	0.798

PCS5|t1	-1.670	0.108 -15.521	0.000	-1.670	-1.670

PCS5|t2	-0.798	0.071 -11.305	0.000	-0.798	-0.798

PCS5|t3	0.006	0.063	0.100	0.920	0.006	0.006

PCS5|t4	0.869	0.072	12.040	0.000	0.869	0.869

PCS6|t1	-1.780	0.116 -15.305	0.000	-1.780	-1.780

PCS6|t2	-0.896	0.073 -12.310	0.000	-0.896	-0.896

PCS6|t3	0.107	0.063	1.698	0.090	0.107	0.107

PCS6|t4	0.906	0.073	12.399	0.000	0.906	0.906

CIS1|t1	-1.598	0.103 -15.577	0.000	-1.598	-1.598

CIS1|t2	-0.860	0.072 -11.949	0.000	-0.860	-0.860

CIS1|t3	0.082	0.063	1.298	0.194	0.082	0.082

CIS1|t4	0.764	0.070	10.931	0.000	0.764	0.764

CIS2|t1	-1.670	0.108 -15.521	0.000	-1.670	-1.670

CIS2|t2	-0.833	0.071 -11.675	0.000	-0.833	-0.833

CIS2|t3	0.075	0.063	1.198	0.231	0.075	0.075

CIS2|t4	0.925	0.074	12.577	0.000	0.925	0.925

CIS3|t1	-1.960	0.134 -14.656	0.000	-1.960	-1.960

CIS3|t2	-0.824	0.071 -11.583	0.000	-0.824	-0.824

CIS3|t3	-0.050	0.063	-0.799	0.424	-0.050	-0.050

CIS3|t4	0.860	0.072	11.949	0.000	0.860	0.860

CIS4|t1	-1.751	0.114 -15.378	0.000	-1.751	-1.751

CIS4|t2	-0.833	0.071 -11.675	0.000	-0.833	-0.833

CIS4|t3	0.025	0.063	0.399	0.690	0.025	0.025

CIS4|t4	0.789	0.070	11.212	0.000	0.789	0.789

CIS5|t1	-1.695	0.109 -15.485	0.000	-1.695	-1.695

CIS5|t2	-0.789	0.070 -11.212	0.000	-0.789	-0.789

CIS5|t3	0.031	0.063	0.499	0.618	0.031	0.031

CIS5|t4	0.860	0.072	11.949	0.000	0.860	0.860

CIS6|t1	-1.695	0.109 -15.485	0.000	-1.695	-1.695

CIS6|t2	-0.789	0.070 -11.212	0.000	-0.789	-0.789

CIS6|t3	-0.006	0.063	-0.100	0.920	-0.006	-0.006

CIS6|t4	0.851	0.072	11.858	0.000	0.851	0.851

Variances:

Estimate Std.Err z-value P(>|z|)	Std.lv Std.all

.HWB1	0.646	0.646	0.646

.HWB2	0.508	0.508	0.508

.HWB3	0.535	0.535	0.535

.HWB4	0.708	0.708	0.708

.HWB5	0.633	0.633	0.633

.HWB6	0.601	0.601	0.601

.AMS1	0.553	0.553	0.553

.AMS2	0.513	0.513	0.513

.AMS3	0.585	0.585	0.585

.AMS4	0.574	0.574	0.574

.AMS5	0.514	0.514	0.514

.AMS6	0.533	0.533	0.533

.PEB1	0.587	0.587	0.587

.PEB2	0.438	0.438	0.438

.PEB3	0.608	0.608	0.608

.PEB4	0.492	0.492	0.492

.PEB5	0.663	0.663	0.663

.PEB6	0.595	0.595	0.595

.PID1	0.577	0.577	0.577

.PID2	0.574	0.574	0.574

.PID3	0.640	0.640	0.640

.PID4	0.499	0.499	0.499

.PID5	0.526	0.526	0.526

.PID6	0.537	0.537	0.537

.PCS1	0.595	0.595	0.595

.PCS2	0.503	0.503	0.503

.PCS3	0.527	0.527	0.527

.PCS4	0.485	0.485	0.485

.PCS5	0.539	0.539	0.539

.PCS6	0.597	0.597	0.597

.CIS1	0.560	0.560	0.560

.CIS2	0.511	0.511	0.511

.CIS3	0.562	0.562	0.562

.CIS4	0.511	0.511	0.511

.CIS5	0.515	0.515	0.515

.CIS6	0.631	0.631	0.631

HWB	1.000	1.000	1.000

AMS	1.000	1.000	1.000

PEB	1.000	1.000	1.000

PID	1.000	1.000	1.000

PCS	1.000	1.000	1.000

CIS	1.000	1.000	1.000

**Annex I-B. Complete residual-correlation and normalized-residual matrices**

The first matrix reports correlation-scale residuals. The second reports normalized residuals (z values). The ranked local-dependence summary in Table I-7 was derived directly from these matrices.

*Table I-B1. Complete residual-correlation matrix, block 1 of 4 (HWB1–AMS3).*

| **Item** | **HWB1** | **HWB2** | **HWB3** | **HWB4** | **HWB5** | **HWB6** | **AMS1** | **AMS2** | **AMS3** |
| --- | --- | --- | --- | --- | --- | --- | --- | --- | --- |
| HWB1 | 0.000 | 0.026 | -0.053 | -0.020 | 0.048 | 0.047 | -0.044 | 0.060 | 0.032 |
| HWB2 | 0.026 | 0.000 | -0.014 | -0.006 | -0.001 | -0.019 | -0.039 | -0.007 | 0.010 |
| HWB3 | -0.053 | -0.014 | 0.000 | 0.021 | 0.001 | -0.045 | 0.007 | 0.041 | 0.024 |
| HWB4 | -0.020 | -0.006 | 0.021 | 0.000 | 0.028 | 0.025 | -0.010 | 0.049 | -0.065 |
| HWB5 | 0.048 | -0.001 | 0.001 | 0.028 | 0.000 | -0.016 | -0.059 | 0.000 | -0.016 |
| HWB6 | 0.047 | -0.019 | -0.045 | 0.025 | -0.016 | 0.000 | 0.018 | -0.044 | 0.013 |
| AMS1 | -0.044 | -0.039 | 0.007 | -0.010 | -0.059 | 0.018 | 0.000 | -0.017 | -0.007 |
| AMS2 | 0.060 | -0.007 | 0.041 | 0.049 | 0.000 | -0.044 | -0.017 | 0.000 | 0.021 |
| AMS3 | 0.032 | 0.010 | 0.024 | -0.065 | -0.016 | 0.013 | -0.007 | 0.021 | 0.000 |
| AMS4 | 0.009 | -0.053 | 0.042 | 0.019 | -0.017 | -0.021 | -0.003 | 0.016 | -0.014 |
| AMS5 | -0.044 | -0.013 | 0.063 | 0.035 | -0.016 | -0.026 | -0.024 | -0.017 | -0.002 |
| AMS6 | 0.019 | 0.008 | 0.012 | -0.059 | -0.015 | 0.015 | -0.023 | 0.009 | 0.031 |
| PEB1 | -0.052 | 0.024 | -0.007 | 0.039 | -0.033 | 0.023 | 0.003 | 0.056 | -0.041 |
| PEB2 | -0.033 | 0.015 | -0.028 | 0.016 | -0.066 | 0.020 | 0.059 | 0.034 | 0.042 |
| PEB3 | 0.001 | 0.041 | -0.062 | -0.050 | -0.038 | -0.035 | 0.014 | -0.009 | 0.034 |
| PEB4 | -0.070 | 0.019 | 0.010 | -0.004 | 0.038 | 0.048 | 0.073 | 0.058 | 0.056 |
| PEB5 | -0.133 | -0.011 | -0.013 | -0.005 | -0.053 | -0.075 | 0.024 | 0.029 | 0.013 |
| PEB6 | 0.057 | 0.112 | 0.055 | 0.077 | 0.047 | 0.006 | -0.003 | 0.015 | -0.046 |
| PID1 | -0.079 | -0.056 | -0.023 | -0.008 | -0.023 | 0.002 | 0.021 | -0.042 | -0.079 |
| PID2 | -0.121 | -0.031 | -0.002 | -0.002 | -0.013 | -0.078 | 0.072 | 0.016 | -0.064 |
| PID3 | 0.014 | -0.020 | 0.027 | 0.133 | -0.002 | 0.023 | 0.047 | -0.033 | -0.026 |
| PID4 | 0.010 | -0.026 | 0.056 | 0.079 | 0.076 | 0.007 | 0.094 | -0.021 | -0.012 |
| PID5 | -0.006 | 0.011 | -0.034 | -0.003 | -0.005 | 0.017 | -0.004 | -0.012 | 0.008 |
| PID6 | -0.032 | -0.038 | -0.030 | 0.136 | 0.017 | 0.033 | 0.013 | -0.010 | -0.043 |
| PCS1 | -0.035 | -0.071 | 0.000 | -0.057 | -0.066 | -0.026 | -0.062 | -0.051 | -0.029 |
| PCS2 | 0.066 | -0.000 | 0.066 | -0.093 | -0.047 | 0.054 | -0.007 | 0.007 | 0.020 |
| PCS3 | 0.000 | 0.034 | -0.003 | -0.042 | -0.040 | 0.053 | -0.013 | -0.061 | -0.005 |
| PCS4 | -0.030 | 0.000 | 0.003 | 0.004 | -0.022 | 0.026 | 0.023 | -0.060 | -0.018 |
| PCS5 | 0.096 | 0.054 | -0.007 | 0.016 | 0.011 | 0.028 | -0.000 | -0.008 | 0.019 |
| PCS6 | 0.047 | 0.012 | -0.018 | -0.054 | -0.007 | -0.044 | -0.015 | -0.070 | 0.036 |
| CIS1 | 0.026 | 0.032 | -0.021 | 0.001 | 0.053 | 0.022 | 0.003 | 0.008 | -0.025 |
| CIS2 | 0.060 | -0.001 | -0.001 | -0.124 | 0.041 | -0.021 | 0.005 | -0.043 | 0.065 |
| CIS3 | -0.048 | -0.011 | -0.038 | -0.041 | 0.016 | -0.010 | -0.008 | 0.049 | -0.008 |
| CIS4 | 0.043 | 0.020 | 0.022 | -0.012 | 0.077 | -0.047 | 0.011 | 0.043 | 0.013 |
| CIS5 | 0.030 | -0.004 | 0.000 | -0.138 | 0.049 | 0.014 | -0.007 | -0.032 | 0.002 |
| CIS6 | -0.024 | -0.065 | 0.016 | -0.025 | 0.021 | 0.025 | -0.088 | -0.004 | -0.064 |

*Table I-B1. Complete residual-correlation matrix, block 2 of 4 (AMS4–PEB6).*

| **Item** | **AMS4** | **AMS5** | **AMS6** | **PEB1** | **PEB2** | **PEB3** | **PEB4** | **PEB5** | **PEB6** |
| --- | --- | --- | --- | --- | --- | --- | --- | --- | --- |
| HWB1 | 0.009 | -0.044 | 0.019 | -0.052 | -0.033 | 0.001 | -0.070 | -0.133 | 0.057 |
| HWB2 | -0.053 | -0.013 | 0.008 | 0.024 | 0.015 | 0.041 | 0.019 | -0.011 | 0.112 |
| HWB3 | 0.042 | 0.063 | 0.012 | -0.007 | -0.028 | -0.062 | 0.010 | -0.013 | 0.055 |
| HWB4 | 0.019 | 0.035 | -0.059 | 0.039 | 0.016 | -0.050 | -0.004 | -0.005 | 0.077 |
| HWB5 | -0.017 | -0.016 | -0.015 | -0.033 | -0.066 | -0.038 | 0.038 | -0.053 | 0.047 |
| HWB6 | -0.021 | -0.026 | 0.015 | 0.023 | 0.020 | -0.035 | 0.048 | -0.075 | 0.006 |
| AMS1 | -0.003 | -0.024 | -0.023 | 0.003 | 0.059 | 0.014 | 0.073 | 0.024 | -0.003 |
| AMS2 | 0.016 | -0.017 | 0.009 | 0.056 | 0.034 | -0.009 | 0.058 | 0.029 | 0.015 |
| AMS3 | -0.014 | -0.002 | 0.031 | -0.041 | 0.042 | 0.034 | 0.056 | 0.013 | -0.046 |
| AMS4 | 0.000 | 0.011 | 0.058 | 0.019 | 0.001 | 0.004 | -0.059 | 0.011 | -0.049 |
| AMS5 | 0.011 | 0.000 | -0.040 | -0.025 | -0.020 | -0.018 | -0.032 | -0.051 | -0.087 |
| AMS6 | 0.058 | -0.040 | 0.000 | -0.095 | -0.013 | -0.058 | -0.016 | 0.026 | -0.024 |
| PEB1 | 0.019 | -0.025 | -0.095 | 0.000 | -0.015 | -0.013 | -0.011 | 0.028 | 0.078 |
| PEB2 | 0.001 | -0.020 | -0.013 | -0.015 | 0.000 | -0.036 | -0.063 | 0.044 | -0.041 |
| PEB3 | 0.004 | -0.018 | -0.058 | -0.013 | -0.036 | 0.000 | -0.022 | 0.064 | 0.048 |
| PEB4 | -0.059 | -0.032 | -0.016 | -0.011 | -0.063 | -0.022 | 0.000 | -0.003 | 0.013 |
| PEB5 | 0.011 | -0.051 | 0.026 | 0.028 | 0.044 | 0.064 | -0.003 | 0.000 | -0.053 |
| PEB6 | -0.049 | -0.087 | -0.024 | 0.078 | -0.041 | 0.048 | 0.013 | -0.053 | 0.000 |
| PID1 | 0.004 | -0.004 | 0.062 | -0.117 | 0.011 | -0.042 | 0.011 | 0.064 | -0.132 |
| PID2 | -0.004 | 0.001 | 0.028 | 0.001 | 0.019 | -0.027 | -0.016 | 0.005 | -0.064 |
| PID3 | 0.015 | 0.023 | 0.063 | -0.076 | -0.005 | -0.073 | 0.035 | -0.013 | -0.006 |
| PID4 | -0.034 | 0.027 | -0.042 | -0.032 | 0.009 | -0.072 | 0.036 | 0.001 | -0.021 |
| PID5 | -0.048 | -0.026 | 0.010 | -0.019 | 0.057 | 0.013 | -0.043 | -0.001 | -0.053 |
| PID6 | -0.043 | 0.015 | -0.013 | 0.040 | 0.079 | 0.102 | 0.076 | 0.062 | 0.060 |
| PCS1 | -0.052 | 0.081 | -0.003 | -0.014 | -0.019 | 0.004 | -0.091 | -0.038 | -0.057 |
| PCS2 | -0.026 | 0.067 | 0.017 | 0.059 | 0.057 | -0.022 | 0.001 | 0.061 | 0.070 |
| PCS3 | -0.020 | 0.038 | -0.039 | -0.046 | 0.038 | 0.015 | -0.032 | -0.067 | 0.005 |
| PCS4 | -0.013 | 0.017 | 0.015 | -0.013 | 0.035 | 0.077 | -0.020 | -0.062 | -0.026 |
| PCS5 | 0.016 | 0.068 | 0.022 | 0.055 | -0.036 | 0.015 | -0.005 | -0.009 | -0.048 |
| PCS6 | -0.030 | 0.048 | 0.005 | -0.018 | 0.051 | -0.012 | -0.015 | -0.064 | 0.057 |
| CIS1 | 0.002 | 0.001 | -0.004 | 0.073 | 0.028 | -0.028 | -0.013 | -0.032 | -0.059 |
| CIS2 | -0.001 | -0.031 | -0.033 | -0.027 | 0.073 | 0.073 | 0.033 | -0.005 | -0.055 |
| CIS3 | -0.009 | 0.021 | 0.029 | -0.024 | -0.049 | -0.053 | 0.038 | -0.003 | 0.017 |
| CIS4 | 0.031 | 0.010 | -0.056 | 0.047 | -0.038 | 0.074 | 0.045 | -0.002 | -0.017 |
| CIS5 | 0.030 | -0.045 | -0.010 | -0.037 | -0.011 | 0.004 | 0.005 | -0.018 | -0.018 |
| CIS6 | 0.058 | -0.005 | 0.044 | -0.009 | -0.076 | -0.001 | -0.039 | 0.045 | -0.061 |

*Table I-B1. Complete residual-correlation matrix, block 3 of 4 (PID1–PCS3).*

| **Item** | **PID1** | **PID2** | **PID3** | **PID4** | **PID5** | **PID6** | **PCS1** | **PCS2** | **PCS3** |
| --- | --- | --- | --- | --- | --- | --- | --- | --- | --- |
| HWB1 | -0.079 | -0.121 | 0.014 | 0.010 | -0.006 | -0.032 | -0.035 | 0.066 | 0.000 |
| HWB2 | -0.056 | -0.031 | -0.020 | -0.026 | 0.011 | -0.038 | -0.071 | -0.000 | 0.034 |
| HWB3 | -0.023 | -0.002 | 0.027 | 0.056 | -0.034 | -0.030 | 0.000 | 0.066 | -0.003 |
| HWB4 | -0.008 | -0.002 | 0.133 | 0.079 | -0.003 | 0.136 | -0.057 | -0.093 | -0.042 |
| HWB5 | -0.023 | -0.013 | -0.002 | 0.076 | -0.005 | 0.017 | -0.066 | -0.047 | -0.040 |
| HWB6 | 0.002 | -0.078 | 0.023 | 0.007 | 0.017 | 0.033 | -0.026 | 0.054 | 0.053 |
| AMS1 | 0.021 | 0.072 | 0.047 | 0.094 | -0.004 | 0.013 | -0.062 | -0.007 | -0.013 |
| AMS2 | -0.042 | 0.016 | -0.033 | -0.021 | -0.012 | -0.010 | -0.051 | 0.007 | -0.061 |
| AMS3 | -0.079 | -0.064 | -0.026 | -0.012 | 0.008 | -0.043 | -0.029 | 0.020 | -0.005 |
| AMS4 | 0.004 | -0.004 | 0.015 | -0.034 | -0.048 | -0.043 | -0.052 | -0.026 | -0.020 |
| AMS5 | -0.004 | 0.001 | 0.023 | 0.027 | -0.026 | 0.015 | 0.081 | 0.067 | 0.038 |
| AMS6 | 0.062 | 0.028 | 0.063 | -0.042 | 0.010 | -0.013 | -0.003 | 0.017 | -0.039 |
| PEB1 | -0.117 | 0.001 | -0.076 | -0.032 | -0.019 | 0.040 | -0.014 | 0.059 | -0.046 |
| PEB2 | 0.011 | 0.019 | -0.005 | 0.009 | 0.057 | 0.079 | -0.019 | 0.057 | 0.038 |
| PEB3 | -0.042 | -0.027 | -0.073 | -0.072 | 0.013 | 0.102 | 0.004 | -0.022 | 0.015 |
| PEB4 | 0.011 | -0.016 | 0.035 | 0.036 | -0.043 | 0.076 | -0.091 | 0.001 | -0.032 |
| PEB5 | 0.064 | 0.005 | -0.013 | 0.001 | -0.001 | 0.062 | -0.038 | 0.061 | -0.067 |
| PEB6 | -0.132 | -0.064 | -0.006 | -0.021 | -0.053 | 0.060 | -0.057 | 0.070 | 0.005 |
| PID1 | 0.000 | 0.060 | 0.029 | -0.016 | 0.004 | -0.022 | 0.038 | 0.036 | 0.092 |
| PID2 | 0.060 | 0.000 | -0.009 | 0.016 | 0.053 | 0.015 | -0.018 | -0.109 | -0.024 |
| PID3 | 0.029 | -0.009 | 0.000 | -0.008 | -0.023 | -0.007 | 0.001 | -0.031 | 0.039 |
| PID4 | -0.016 | 0.016 | -0.008 | 0.000 | -0.021 | -0.005 | -0.024 | -0.034 | 0.049 |
| PID5 | 0.004 | 0.053 | -0.023 | -0.021 | 0.000 | -0.087 | 0.034 | -0.043 | 0.078 |
| PID6 | -0.022 | 0.015 | -0.007 | -0.005 | -0.087 | 0.000 | 0.008 | -0.062 | 0.009 |
| PCS1 | 0.038 | -0.018 | 0.001 | -0.024 | 0.034 | 0.008 | 0.000 | 0.007 | 0.011 |
| PCS2 | 0.036 | -0.109 | -0.031 | -0.034 | -0.043 | -0.062 | 0.007 | 0.000 | -0.021 |
| PCS3 | 0.092 | -0.024 | 0.039 | 0.049 | 0.078 | 0.009 | 0.011 | -0.021 | 0.000 |
| PCS4 | 0.017 | -0.060 | 0.015 | 0.033 | 0.041 | -0.042 | 0.048 | 0.029 | -0.029 |
| PCS5 | 0.031 | -0.047 | -0.037 | 0.009 | 0.045 | 0.014 | -0.019 | -0.031 | 0.024 |
| PCS6 | 0.020 | -0.061 | 0.039 | -0.037 | -0.045 | -0.015 | 0.048 | 0.010 | -0.020 |
| CIS1 | -0.007 | 0.058 | -0.015 | 0.027 | 0.085 | 0.033 | -0.002 | -0.034 | 0.009 |
| CIS2 | -0.048 | -0.011 | -0.054 | -0.006 | 0.046 | -0.001 | -0.005 | -0.045 | 0.012 |
| CIS3 | 0.028 | 0.034 | 0.068 | 0.017 | 0.050 | -0.015 | 0.072 | -0.049 | -0.009 |
| CIS4 | 0.004 | 0.015 | -0.023 | -0.037 | -0.083 | -0.005 | 0.038 | 0.013 | 0.032 |
| CIS5 | -0.066 | -0.031 | -0.051 | -0.033 | 0.028 | -0.030 | -0.034 | -0.035 | -0.019 |
| CIS6 | 0.033 | -0.035 | 0.001 | -0.002 | 0.045 | -0.014 | 0.011 | -0.028 | -0.001 |

*Table I-B1. Complete residual-correlation matrix, block 4 of 4 (PCS4–CIS6).*

| **Item** | **PCS4** | **PCS5** | **PCS6** | **CIS1** | **CIS2** | **CIS3** | **CIS4** | **CIS5** | **CIS6** |
| --- | --- | --- | --- | --- | --- | --- | --- | --- | --- |
| HWB1 | -0.030 | 0.096 | 0.047 | 0.026 | 0.060 | -0.048 | 0.043 | 0.030 | -0.024 |
| HWB2 | 0.000 | 0.054 | 0.012 | 0.032 | -0.001 | -0.011 | 0.020 | -0.004 | -0.065 |
| HWB3 | 0.003 | -0.007 | -0.018 | -0.021 | -0.001 | -0.038 | 0.022 | 0.000 | 0.016 |
| HWB4 | 0.004 | 0.016 | -0.054 | 0.001 | -0.124 | -0.041 | -0.012 | -0.138 | -0.025 |
| HWB5 | -0.022 | 0.011 | -0.007 | 0.053 | 0.041 | 0.016 | 0.077 | 0.049 | 0.021 |
| HWB6 | 0.026 | 0.028 | -0.044 | 0.022 | -0.021 | -0.010 | -0.047 | 0.014 | 0.025 |
| AMS1 | 0.023 | -0.000 | -0.015 | 0.003 | 0.005 | -0.008 | 0.011 | -0.007 | -0.088 |
| AMS2 | -0.060 | -0.008 | -0.070 | 0.008 | -0.043 | 0.049 | 0.043 | -0.032 | -0.004 |
| AMS3 | -0.018 | 0.019 | 0.036 | -0.025 | 0.065 | -0.008 | 0.013 | 0.002 | -0.064 |
| AMS4 | -0.013 | 0.016 | -0.030 | 0.002 | -0.001 | -0.009 | 0.031 | 0.030 | 0.058 |
| AMS5 | 0.017 | 0.068 | 0.048 | 0.001 | -0.031 | 0.021 | 0.010 | -0.045 | -0.005 |
| AMS6 | 0.015 | 0.022 | 0.005 | -0.004 | -0.033 | 0.029 | -0.056 | -0.010 | 0.044 |
| PEB1 | -0.013 | 0.055 | -0.018 | 0.073 | -0.027 | -0.024 | 0.047 | -0.037 | -0.009 |
| PEB2 | 0.035 | -0.036 | 0.051 | 0.028 | 0.073 | -0.049 | -0.038 | -0.011 | -0.076 |
| PEB3 | 0.077 | 0.015 | -0.012 | -0.028 | 0.073 | -0.053 | 0.074 | 0.004 | -0.001 |
| PEB4 | -0.020 | -0.005 | -0.015 | -0.013 | 0.033 | 0.038 | 0.045 | 0.005 | -0.039 |
| PEB5 | -0.062 | -0.009 | -0.064 | -0.032 | -0.005 | -0.003 | -0.002 | -0.018 | 0.045 |
| PEB6 | -0.026 | -0.048 | 0.057 | -0.059 | -0.055 | 0.017 | -0.017 | -0.018 | -0.061 |
| PID1 | 0.017 | 0.031 | 0.020 | -0.007 | -0.048 | 0.028 | 0.004 | -0.066 | 0.033 |
| PID2 | -0.060 | -0.047 | -0.061 | 0.058 | -0.011 | 0.034 | 0.015 | -0.031 | -0.035 |
| PID3 | 0.015 | -0.037 | 0.039 | -0.015 | -0.054 | 0.068 | -0.023 | -0.051 | 0.001 |
| PID4 | 0.033 | 0.009 | -0.037 | 0.027 | -0.006 | 0.017 | -0.037 | -0.033 | -0.002 |
| PID5 | 0.041 | 0.045 | -0.045 | 0.085 | 0.046 | 0.050 | -0.083 | 0.028 | 0.045 |
| PID6 | -0.042 | 0.014 | -0.015 | 0.033 | -0.001 | -0.015 | -0.005 | -0.030 | -0.014 |
| PCS1 | 0.048 | -0.019 | 0.048 | -0.002 | -0.005 | 0.072 | 0.038 | -0.034 | 0.011 |
| PCS2 | 0.029 | -0.031 | 0.010 | -0.034 | -0.045 | -0.049 | 0.013 | -0.035 | -0.028 |
| PCS3 | -0.029 | 0.024 | -0.020 | 0.009 | 0.012 | -0.009 | 0.032 | -0.019 | -0.001 |
| PCS4 | 0.000 | -0.077 | -0.014 | 0.078 | -0.011 | -0.008 | -0.005 | 0.017 | 0.028 |
| PCS5 | -0.077 | 0.000 | 0.003 | -0.001 | -0.013 | -0.043 | 0.008 | -0.063 | -0.012 |
| PCS6 | -0.014 | 0.003 | 0.000 | 0.006 | 0.103 | -0.020 | -0.006 | 0.028 | -0.049 |
| CIS1 | 0.078 | -0.001 | 0.006 | 0.000 | -0.050 | -0.029 | -0.017 | 0.014 | -0.037 |
| CIS2 | -0.011 | -0.013 | 0.103 | -0.050 | 0.000 | -0.010 | -0.018 | 0.038 | 0.012 |
| CIS3 | -0.008 | -0.043 | -0.020 | -0.029 | -0.010 | 0.000 | -0.055 | 0.042 | 0.028 |
| CIS4 | -0.005 | 0.008 | -0.006 | -0.017 | -0.018 | -0.055 | 0.000 | 0.003 | 0.018 |
| CIS5 | 0.017 | -0.063 | 0.028 | 0.014 | 0.038 | 0.042 | 0.003 | 0.000 | 0.037 |
| CIS6 | 0.028 | -0.012 | -0.049 | -0.037 | 0.012 | 0.028 | 0.018 | 0.037 | 0.000 |

*Table I-B2. Complete normalized-residual matrix, block 1 of 4 (HWB1–AMS3).*

| **Item** | **HWB1** | **HWB2** | **HWB3** | **HWB4** | **HWB5** | **HWB6** | **AMS1** | **AMS2** | **AMS3** |
| --- | --- | --- | --- | --- | --- | --- | --- | --- | --- |
| HWB1 | 0.00 | 0.70 | -1.59 | -0.49 | 1.21 | 1.23 | -1.03 | 1.43 | 0.71 |
| HWB2 | 0.70 | 0.00 | -0.38 | -0.14 | -0.04 | -0.60 | -0.94 | -0.17 | 0.25 |
| HWB3 | -1.59 | -0.38 | 0.00 | 0.58 | 0.03 | -1.26 | 0.19 | 1.14 | 0.59 |
| HWB4 | -0.49 | -0.14 | 0.58 | 0.00 | 0.64 | 0.68 | -0.25 | 1.19 | -1.70 |
| HWB5 | 1.21 | -0.04 | 0.03 | 0.64 | 0.00 | -0.40 | -1.35 | 0.00 | -0.38 |
| HWB6 | 1.23 | -0.60 | -1.26 | 0.68 | -0.40 | 0.00 | 0.44 | -1.19 | 0.35 |
| AMS1 | -1.03 | -0.94 | 0.19 | -0.25 | -1.35 | 0.44 | 0.00 | -0.53 | -0.20 |
| AMS2 | 1.43 | -0.17 | 1.14 | 1.19 | 0.00 | -1.19 | -0.53 | 0.00 | 0.60 |
| AMS3 | 0.71 | 0.25 | 0.59 | -1.70 | -0.38 | 0.35 | -0.20 | 0.60 | 0.00 |
| AMS4 | 0.25 | -1.45 | 1.15 | 0.47 | -0.43 | -0.59 | -0.07 | 0.48 | -0.34 |
| AMS5 | -1.06 | -0.36 | 1.73 | 0.93 | -0.42 | -0.67 | -0.74 | -0.57 | -0.06 |
| AMS6 | 0.49 | 0.21 | 0.31 | -1.42 | -0.40 | 0.38 | -0.65 | 0.26 | 0.92 |
| PEB1 | -1.20 | 0.58 | -0.17 | 0.98 | -0.81 | 0.54 | 0.08 | 1.35 | -0.96 |
| PEB2 | -0.88 | 0.38 | -0.69 | 0.44 | -1.79 | 0.54 | 1.63 | 0.84 | 1.03 |
| PEB3 | 0.02 | 1.03 | -1.62 | -1.17 | -0.85 | -0.82 | 0.36 | -0.23 | 0.81 |
| PEB4 | -1.69 | 0.55 | 0.28 | -0.10 | 0.94 | 1.25 | 1.87 | 1.64 | 1.52 |
| PEB5 | -3.17 | -0.26 | -0.34 | -0.12 | -1.35 | -1.76 | 0.65 | 0.74 | 0.31 |
| PEB6 | 1.35 | 2.89 | 1.35 | 2.03 | 1.15 | 0.14 | -0.08 | 0.41 | -1.23 |
| PID1 | -1.88 | -1.43 | -0.56 | -0.19 | -0.53 | 0.05 | 0.56 | -0.98 | -1.97 |
| PID2 | -2.84 | -0.76 | -0.04 | -0.05 | -0.32 | -1.83 | 1.75 | 0.41 | -1.65 |
| PID3 | 0.29 | -0.48 | 0.60 | 3.06 | -0.05 | 0.52 | 1.30 | -0.86 | -0.63 |
| PID4 | 0.21 | -0.68 | 1.40 | 1.97 | 1.64 | 0.15 | 2.49 | -0.55 | -0.30 |
| PID5 | -0.15 | 0.27 | -0.80 | -0.07 | -0.11 | 0.40 | -0.11 | -0.30 | 0.20 |
| PID6 | -0.77 | -0.92 | -0.73 | 3.44 | 0.46 | 0.82 | 0.34 | -0.24 | -1.16 |
| PCS1 | -0.80 | -1.73 | 0.01 | -1.27 | -1.49 | -0.58 | -1.52 | -1.22 | -0.80 |
| PCS2 | 1.58 | -0.01 | 1.54 | -2.34 | -1.22 | 1.32 | -0.18 | 0.17 | 0.56 |
| PCS3 | 0.00 | 0.89 | -0.07 | -0.97 | -1.02 | 1.23 | -0.33 | -1.69 | -0.12 |
| PCS4 | -0.67 | 0.00 | 0.08 | 0.08 | -0.58 | 0.61 | 0.57 | -1.68 | -0.51 |
| PCS5 | 2.17 | 1.55 | -0.18 | 0.34 | 0.28 | 0.64 | -0.01 | -0.19 | 0.49 |
| PCS6 | 1.01 | 0.29 | -0.42 | -1.26 | -0.16 | -0.97 | -0.37 | -1.72 | 0.85 |
| CIS1 | 0.58 | 0.86 | -0.50 | 0.01 | 1.39 | 0.58 | 0.07 | 0.19 | -0.61 |
| CIS2 | 1.56 | -0.02 | -0.03 | -2.88 | 0.91 | -0.52 | 0.13 | -1.08 | 1.57 |
| CIS3 | -1.16 | -0.25 | -0.84 | -0.78 | 0.36 | -0.25 | -0.17 | 1.17 | -0.17 |
| CIS4 | 1.02 | 0.48 | 0.56 | -0.26 | 1.73 | -1.24 | 0.27 | 1.11 | 0.33 |
| CIS5 | 0.68 | -0.09 | 0.00 | -3.32 | 1.09 | 0.36 | -0.17 | -0.76 | 0.06 |
| CIS6 | -0.60 | -1.42 | 0.44 | -0.59 | 0.51 | 0.57 | -1.97 | -0.09 | -1.40 |

*Table I-B2. Complete normalized-residual matrix, block 2 of 4 (AMS4–PEB6).*

| **Item** | **AMS4** | **AMS5** | **AMS6** | **PEB1** | **PEB2** | **PEB3** | **PEB4** | **PEB5** | **PEB6** |
| --- | --- | --- | --- | --- | --- | --- | --- | --- | --- |
| HWB1 | 0.25 | -1.06 | 0.49 | -1.20 | -0.88 | 0.02 | -1.69 | -3.17 | 1.35 |
| HWB2 | -1.45 | -0.36 | 0.21 | 0.58 | 0.38 | 1.03 | 0.55 | -0.26 | 2.89 |
| HWB3 | 1.15 | 1.73 | 0.31 | -0.17 | -0.69 | -1.62 | 0.28 | -0.34 | 1.35 |
| HWB4 | 0.47 | 0.93 | -1.42 | 0.98 | 0.44 | -1.17 | -0.10 | -0.12 | 2.03 |
| HWB5 | -0.43 | -0.42 | -0.40 | -0.81 | -1.79 | -0.85 | 0.94 | -1.35 | 1.15 |
| HWB6 | -0.59 | -0.67 | 0.38 | 0.54 | 0.54 | -0.82 | 1.25 | -1.76 | 0.14 |
| AMS1 | -0.07 | -0.74 | -0.65 | 0.08 | 1.63 | 0.36 | 1.87 | 0.65 | -0.08 |
| AMS2 | 0.48 | -0.57 | 0.26 | 1.35 | 0.84 | -0.23 | 1.64 | 0.74 | 0.41 |
| AMS3 | -0.34 | -0.06 | 0.92 | -0.96 | 1.03 | 0.81 | 1.52 | 0.31 | -1.23 |
| AMS4 | 0.00 | 0.34 | 1.56 | 0.50 | 0.03 | 0.12 | -1.43 | 0.26 | -1.24 |
| AMS5 | 0.34 | 0.00 | -1.20 | -0.62 | -0.54 | -0.45 | -0.83 | -1.32 | -2.51 |
| AMS6 | 1.56 | -1.20 | 0.00 | -2.47 | -0.33 | -1.54 | -0.38 | 0.65 | -0.63 |
| PEB1 | 0.50 | -0.62 | -2.47 | 0.00 | -0.43 | -0.34 | -0.31 | 0.81 | 1.96 |
| PEB2 | 0.03 | -0.54 | -0.33 | -0.43 | 0.00 | -1.07 | -1.97 | 1.26 | -1.20 |
| PEB3 | 0.12 | -0.45 | -1.54 | -0.34 | -1.07 | 0.00 | -0.59 | 1.56 | 1.26 |
| PEB4 | -1.43 | -0.83 | -0.38 | -0.31 | -1.97 | -0.59 | 0.00 | -0.07 | 0.38 |
| PEB5 | 0.26 | -1.32 | 0.65 | 0.81 | 1.26 | 1.56 | -0.07 | 0.00 | -1.47 |
| PEB6 | -1.24 | -2.51 | -0.63 | 1.96 | -1.20 | 1.26 | 0.38 | -1.47 | 0.00 |
| PID1 | 0.11 | -0.10 | 1.50 | -2.89 | 0.27 | -1.01 | 0.29 | 1.50 | -3.01 |
| PID2 | -0.11 | 0.02 | 0.71 | 0.02 | 0.53 | -0.69 | -0.43 | 0.13 | -1.54 |
| PID3 | 0.34 | 0.55 | 1.51 | -1.81 | -0.12 | -1.77 | 0.94 | -0.30 | -0.15 |
| PID4 | -0.82 | 0.75 | -1.05 | -0.75 | 0.22 | -1.71 | 1.01 | 0.02 | -0.53 |
| PID5 | -1.17 | -0.64 | 0.27 | -0.46 | 1.34 | 0.30 | -1.14 | -0.02 | -1.35 |
| PID6 | -1.07 | 0.37 | -0.35 | 1.02 | 2.13 | 2.48 | 1.93 | 1.62 | 1.43 |
| PCS1 | -1.28 | 2.21 | -0.07 | -0.33 | -0.47 | 0.09 | -2.09 | -0.86 | -1.29 |
| PCS2 | -0.73 | 1.77 | 0.45 | 1.54 | 1.54 | -0.51 | 0.02 | 1.49 | 1.76 |
| PCS3 | -0.52 | 0.95 | -1.03 | -1.10 | 0.93 | 0.36 | -0.81 | -1.57 | 0.13 |
| PCS4 | -0.33 | 0.49 | 0.43 | -0.34 | 0.89 | 1.87 | -0.50 | -1.56 | -0.58 |
| PCS5 | 0.45 | 1.88 | 0.57 | 1.40 | -0.85 | 0.33 | -0.10 | -0.21 | -1.14 |
| PCS6 | -0.69 | 1.19 | 0.12 | -0.44 | 1.29 | -0.26 | -0.36 | -1.45 | 1.29 |
| CIS1 | 0.04 | 0.02 | -0.10 | 1.85 | 0.71 | -0.72 | -0.31 | -0.79 | -1.45 |
| CIS2 | -0.02 | -0.80 | -0.90 | -0.68 | 2.01 | 1.94 | 0.86 | -0.12 | -1.28 |
| CIS3 | -0.22 | 0.47 | 0.77 | -0.56 | -1.23 | -1.26 | 0.90 | -0.06 | 0.36 |
| CIS4 | 0.70 | 0.29 | -1.50 | 1.26 | -0.98 | 2.01 | 1.19 | -0.06 | -0.42 |
| CIS5 | 0.72 | -1.09 | -0.29 | -0.97 | -0.26 | 0.11 | 0.13 | -0.43 | -0.42 |
| CIS6 | 1.51 | -0.12 | 1.22 | -0.22 | -1.84 | -0.03 | -0.92 | 1.01 | -1.36 |

*Table I-B2. Complete normalized-residual matrix, block 3 of 4 (PID1–PCS3).*

| **Item** | **PID1** | **PID2** | **PID3** | **PID4** | **PID5** | **PID6** | **PCS1** | **PCS2** | **PCS3** |
| --- | --- | --- | --- | --- | --- | --- | --- | --- | --- |
| HWB1 | -1.88 | -2.84 | 0.29 | 0.21 | -0.15 | -0.77 | -0.80 | 1.58 | 0.00 |
| HWB2 | -1.43 | -0.76 | -0.48 | -0.68 | 0.27 | -0.92 | -1.73 | -0.01 | 0.89 |
| HWB3 | -0.56 | -0.04 | 0.60 | 1.40 | -0.80 | -0.73 | 0.01 | 1.54 | -0.07 |
| HWB4 | -0.19 | -0.05 | 3.06 | 1.97 | -0.07 | 3.44 | -1.27 | -2.34 | -0.97 |
| HWB5 | -0.53 | -0.32 | -0.05 | 1.64 | -0.11 | 0.46 | -1.49 | -1.22 | -1.02 |
| HWB6 | 0.05 | -1.83 | 0.52 | 0.15 | 0.40 | 0.82 | -0.58 | 1.32 | 1.23 |
| AMS1 | 0.56 | 1.75 | 1.30 | 2.49 | -0.11 | 0.34 | -1.52 | -0.18 | -0.33 |
| AMS2 | -0.98 | 0.41 | -0.86 | -0.55 | -0.30 | -0.24 | -1.22 | 0.17 | -1.69 |
| AMS3 | -1.97 | -1.65 | -0.63 | -0.30 | 0.20 | -1.16 | -0.80 | 0.56 | -0.12 |
| AMS4 | 0.11 | -0.11 | 0.34 | -0.82 | -1.17 | -1.07 | -1.28 | -0.73 | -0.52 |
| AMS5 | -0.10 | 0.02 | 0.55 | 0.75 | -0.64 | 0.37 | 2.21 | 1.77 | 0.95 |
| AMS6 | 1.50 | 0.71 | 1.51 | -1.05 | 0.27 | -0.35 | -0.07 | 0.45 | -1.03 |
| PEB1 | -2.89 | 0.02 | -1.81 | -0.75 | -0.46 | 1.02 | -0.33 | 1.54 | -1.10 |
| PEB2 | 0.27 | 0.53 | -0.12 | 0.22 | 1.34 | 2.13 | -0.47 | 1.54 | 0.93 |
| PEB3 | -1.01 | -0.69 | -1.77 | -1.71 | 0.30 | 2.48 | 0.09 | -0.51 | 0.36 |
| PEB4 | 0.29 | -0.43 | 0.94 | 1.01 | -1.14 | 1.93 | -2.09 | 0.02 | -0.81 |
| PEB5 | 1.50 | 0.13 | -0.30 | 0.02 | -0.02 | 1.62 | -0.86 | 1.49 | -1.57 |
| PEB6 | -3.01 | -1.54 | -0.15 | -0.53 | -1.35 | 1.43 | -1.29 | 1.76 | 0.13 |
| PID1 | 0.00 | 1.64 | 0.75 | -0.39 | 0.12 | -0.63 | 0.95 | 0.87 | 2.36 |
| PID2 | 1.64 | 0.00 | -0.27 | 0.43 | 1.58 | 0.46 | -0.46 | -2.70 | -0.58 |
| PID3 | 0.75 | -0.27 | 0.00 | -0.22 | -0.65 | -0.19 | 0.03 | -0.75 | 0.95 |
| PID4 | -0.39 | 0.43 | -0.22 | 0.00 | -0.60 | -0.14 | -0.64 | -0.93 | 1.31 |
| PID5 | 0.12 | 1.58 | -0.65 | -0.60 | 0.00 | -2.59 | 0.86 | -1.08 | 1.88 |
| PID6 | -0.63 | 0.46 | -0.19 | -0.14 | -2.59 | 0.00 | 0.18 | -1.48 | 0.22 |
| PCS1 | 0.95 | -0.46 | 0.03 | -0.64 | 0.86 | 0.18 | 0.00 | 0.23 | 0.35 |
| PCS2 | 0.87 | -2.70 | -0.75 | -0.93 | -1.08 | -1.48 | 0.23 | 0.00 | -0.57 |
| PCS3 | 2.36 | -0.58 | 0.95 | 1.31 | 1.88 | 0.22 | 0.35 | -0.57 | 0.00 |
| PCS4 | 0.45 | -1.75 | 0.38 | 0.92 | 1.12 | -1.08 | 1.54 | 0.87 | -0.85 |
| PCS5 | 0.78 | -1.10 | -0.83 | 0.21 | 1.05 | 0.35 | -0.57 | -0.90 | 0.68 |
| PCS6 | 0.51 | -1.53 | 0.81 | -0.90 | -1.10 | -0.38 | 1.21 | 0.27 | -0.55 |
| CIS1 | -0.19 | 1.33 | -0.32 | 0.63 | 1.97 | 0.76 | -0.06 | -0.81 | 0.21 |
| CIS2 | -1.35 | -0.28 | -1.27 | -0.16 | 1.14 | -0.03 | -0.14 | -1.16 | 0.30 |
| CIS3 | 0.73 | 0.81 | 1.63 | 0.41 | 1.17 | -0.34 | 1.66 | -1.13 | -0.23 |
| CIS4 | 0.12 | 0.38 | -0.63 | -0.99 | -2.28 | -0.13 | 0.91 | 0.31 | 0.79 |
| CIS5 | -1.87 | -0.73 | -1.26 | -0.88 | 0.63 | -0.72 | -0.84 | -0.91 | -0.43 |
| CIS6 | 0.91 | -0.89 | 0.02 | -0.04 | 1.20 | -0.32 | 0.29 | -0.72 | -0.03 |

*Table I-B2. Complete normalized-residual matrix, block 4 of 4 (PCS4–CIS6).*

| **Item** | **PCS4** | **PCS5** | **PCS6** | **CIS1** | **CIS2** | **CIS3** | **CIS4** | **CIS5** | **CIS6** |
| --- | --- | --- | --- | --- | --- | --- | --- | --- | --- |
| HWB1 | -0.67 | 2.17 | 1.01 | 0.58 | 1.56 | -1.16 | 1.02 | 0.68 | -0.60 |
| HWB2 | 0.00 | 1.55 | 0.29 | 0.86 | -0.02 | -0.25 | 0.48 | -0.09 | -1.42 |
| HWB3 | 0.08 | -0.18 | -0.42 | -0.50 | -0.03 | -0.84 | 0.56 | 0.00 | 0.44 |
| HWB4 | 0.08 | 0.34 | -1.26 | 0.01 | -2.88 | -0.78 | -0.26 | -3.32 | -0.59 |
| HWB5 | -0.58 | 0.28 | -0.16 | 1.39 | 0.91 | 0.36 | 1.73 | 1.09 | 0.51 |
| HWB6 | 0.61 | 0.64 | -0.97 | 0.58 | -0.52 | -0.25 | -1.24 | 0.36 | 0.57 |
| AMS1 | 0.57 | -0.01 | -0.37 | 0.07 | 0.13 | -0.17 | 0.27 | -0.17 | -1.97 |
| AMS2 | -1.68 | -0.19 | -1.72 | 0.19 | -1.08 | 1.17 | 1.11 | -0.76 | -0.09 |
| AMS3 | -0.51 | 0.49 | 0.85 | -0.61 | 1.57 | -0.17 | 0.33 | 0.06 | -1.40 |
| AMS4 | -0.33 | 0.45 | -0.69 | 0.04 | -0.02 | -0.22 | 0.70 | 0.72 | 1.51 |
| AMS5 | 0.49 | 1.88 | 1.19 | 0.02 | -0.80 | 0.47 | 0.29 | -1.09 | -0.12 |
| AMS6 | 0.43 | 0.57 | 0.12 | -0.10 | -0.90 | 0.77 | -1.50 | -0.29 | 1.22 |
| PEB1 | -0.34 | 1.40 | -0.44 | 1.85 | -0.68 | -0.56 | 1.26 | -0.97 | -0.22 |
| PEB2 | 0.89 | -0.85 | 1.29 | 0.71 | 2.01 | -1.23 | -0.98 | -0.26 | -1.84 |
| PEB3 | 1.87 | 0.33 | -0.26 | -0.72 | 1.94 | -1.26 | 2.01 | 0.11 | -0.03 |
| PEB4 | -0.50 | -0.10 | -0.36 | -0.31 | 0.86 | 0.90 | 1.19 | 0.13 | -0.92 |
| PEB5 | -1.56 | -0.21 | -1.45 | -0.79 | -0.12 | -0.06 | -0.06 | -0.43 | 1.01 |
| PEB6 | -0.58 | -1.14 | 1.29 | -1.45 | -1.28 | 0.36 | -0.42 | -0.42 | -1.36 |
| PID1 | 0.45 | 0.78 | 0.51 | -0.19 | -1.35 | 0.73 | 0.12 | -1.87 | 0.91 |
| PID2 | -1.75 | -1.10 | -1.53 | 1.33 | -0.28 | 0.81 | 0.38 | -0.73 | -0.89 |
| PID3 | 0.38 | -0.83 | 0.81 | -0.32 | -1.27 | 1.63 | -0.63 | -1.26 | 0.02 |
| PID4 | 0.92 | 0.21 | -0.90 | 0.63 | -0.16 | 0.41 | -0.99 | -0.88 | -0.04 |
| PID5 | 1.12 | 1.05 | -1.10 | 1.97 | 1.14 | 1.17 | -2.28 | 0.63 | 1.20 |
| PID6 | -1.08 | 0.35 | -0.38 | 0.76 | -0.03 | -0.34 | -0.13 | -0.72 | -0.32 |
| PCS1 | 1.54 | -0.57 | 1.21 | -0.06 | -0.14 | 1.66 | 0.91 | -0.84 | 0.29 |
| PCS2 | 0.87 | -0.90 | 0.27 | -0.81 | -1.16 | -1.13 | 0.31 | -0.91 | -0.72 |
| PCS3 | -0.85 | 0.68 | -0.55 | 0.21 | 0.30 | -0.23 | 0.79 | -0.43 | -0.03 |
| PCS4 | 0.00 | -2.13 | -0.45 | 1.90 | -0.28 | -0.20 | -0.14 | 0.38 | 0.65 |
| PCS5 | -2.13 | 0.00 | 0.07 | -0.03 | -0.32 | -0.99 | 0.19 | -1.37 | -0.30 |
| PCS6 | -0.45 | 0.07 | 0.00 | 0.16 | 2.72 | -0.43 | -0.14 | 0.64 | -1.17 |
| CIS1 | 1.90 | -0.03 | 0.16 | 0.00 | -1.49 | -0.74 | -0.49 | 0.43 | -1.14 |
| CIS2 | -0.28 | -0.32 | 2.72 | -1.49 | 0.00 | -0.29 | -0.55 | 1.17 | 0.33 |
| CIS3 | -0.20 | -0.99 | -0.43 | -0.74 | -0.29 | 0.00 | -1.62 | 1.17 | 0.77 |
| CIS4 | -0.14 | 0.19 | -0.14 | -0.49 | -0.55 | -1.62 | 0.00 | 0.10 | 0.52 |
| CIS5 | 0.38 | -1.37 | 0.64 | 0.43 | 1.17 | 1.17 | 0.10 | 0.00 | 1.08 |
| CIS6 | 0.65 | -0.30 | -1.17 | -1.14 | 0.33 | 0.77 | 0.52 | 1.08 | 0.00 |

**Annex I-C. Complete polychoric correlation matrix**

This is the 36 × 36 sample polychoric correlation matrix for the archived 400-case CFA subsample. It is also supplied as polychoric_correlation_matrix_cfa_subsample.csv.

*Table I-C1. CFA-subsample polychoric correlation matrix, block 1 of 4 (HWB1–AMS3).*

| **Item** | **HWB1** | **HWB2** | **HWB3** | **HWB4** | **HWB5** | **HWB6** | **AMS1** | **AMS2** | **AMS3** |
| --- | --- | --- | --- | --- | --- | --- | --- | --- | --- |
| HWB1 | 1.000 | 0.443 | 0.352 | 0.302 | 0.408 | 0.422 | 0.169 | 0.282 | 0.237 |
| HWB2 | 0.443 | 1.000 | 0.464 | 0.373 | 0.423 | 0.424 | 0.212 | 0.255 | 0.252 |
| HWB3 | 0.352 | 0.464 | 1.000 | 0.389 | 0.414 | 0.386 | 0.251 | 0.295 | 0.258 |
| HWB4 | 0.302 | 0.373 | 0.389 | 1.000 | 0.355 | 0.366 | 0.183 | 0.250 | 0.121 |
| HWB5 | 0.408 | 0.423 | 0.414 | 0.355 | 1.000 | 0.366 | 0.158 | 0.226 | 0.192 |
| HWB6 | 0.422 | 0.424 | 0.386 | 0.366 | 0.366 | 1.000 | 0.244 | 0.192 | 0.230 |
| AMS1 | 0.169 | 0.212 | 0.251 | 0.183 | 0.158 | 0.244 | 1.000 | 0.450 | 0.424 |
| AMS2 | 0.282 | 0.255 | 0.295 | 0.250 | 0.226 | 0.192 | 0.450 | 1.000 | 0.470 |
| AMS3 | 0.237 | 0.252 | 0.258 | 0.121 | 0.192 | 0.230 | 0.424 | 0.470 | 1.000 |
| AMS4 | 0.217 | 0.192 | 0.280 | 0.207 | 0.194 | 0.199 | 0.434 | 0.471 | 0.407 |
| AMS5 | 0.177 | 0.249 | 0.317 | 0.236 | 0.209 | 0.209 | 0.442 | 0.469 | 0.447 |
| AMS6 | 0.237 | 0.264 | 0.261 | 0.138 | 0.206 | 0.246 | 0.434 | 0.485 | 0.471 |
| PEB1 | 0.150 | 0.262 | 0.224 | 0.222 | 0.172 | 0.237 | 0.226 | 0.288 | 0.174 |
| PEB2 | 0.203 | 0.292 | 0.242 | 0.230 | 0.173 | 0.270 | 0.319 | 0.306 | 0.293 |
| PEB3 | 0.198 | 0.274 | 0.164 | 0.129 | 0.163 | 0.174 | 0.231 | 0.218 | 0.243 |
| PEB4 | 0.154 | 0.283 | 0.266 | 0.199 | 0.266 | 0.285 | 0.321 | 0.316 | 0.294 |
| PEB5 | 0.050 | 0.204 | 0.196 | 0.161 | 0.133 | 0.118 | 0.226 | 0.239 | 0.207 |
| PEB6 | 0.257 | 0.348 | 0.284 | 0.259 | 0.251 | 0.218 | 0.218 | 0.246 | 0.167 |
| PID1 | 0.070 | 0.120 | 0.148 | 0.127 | 0.129 | 0.161 | 0.210 | 0.156 | 0.103 |
| PID2 | 0.029 | 0.146 | 0.170 | 0.134 | 0.140 | 0.081 | 0.261 | 0.214 | 0.119 |
| PID3 | 0.151 | 0.143 | 0.185 | 0.259 | 0.138 | 0.169 | 0.221 | 0.149 | 0.142 |
| PID4 | 0.172 | 0.166 | 0.242 | 0.227 | 0.241 | 0.179 | 0.299 | 0.193 | 0.187 |
| PID5 | 0.152 | 0.197 | 0.147 | 0.141 | 0.156 | 0.185 | 0.196 | 0.197 | 0.200 |
| PID6 | 0.124 | 0.147 | 0.149 | 0.278 | 0.176 | 0.199 | 0.210 | 0.196 | 0.148 |
| PCS1 | 0.128 | 0.122 | 0.188 | 0.092 | 0.101 | 0.147 | 0.182 | 0.203 | 0.206 |
| PCS2 | 0.247 | 0.213 | 0.274 | 0.071 | 0.137 | 0.247 | 0.263 | 0.288 | 0.280 |
| PCS3 | 0.177 | 0.243 | 0.200 | 0.119 | 0.140 | 0.240 | 0.251 | 0.213 | 0.249 |
| PCS4 | 0.155 | 0.217 | 0.214 | 0.171 | 0.165 | 0.222 | 0.297 | 0.227 | 0.246 |
| PCS5 | 0.271 | 0.260 | 0.193 | 0.174 | 0.189 | 0.213 | 0.260 | 0.263 | 0.270 |
| PCS6 | 0.210 | 0.204 | 0.169 | 0.094 | 0.159 | 0.129 | 0.228 | 0.184 | 0.270 |
| CIS1 | 0.196 | 0.233 | 0.173 | 0.155 | 0.226 | 0.203 | 0.203 | 0.217 | 0.167 |
| CIS2 | 0.239 | 0.211 | 0.204 | 0.039 | 0.223 | 0.169 | 0.216 | 0.177 | 0.268 |
| CIS3 | 0.121 | 0.189 | 0.156 | 0.113 | 0.189 | 0.169 | 0.191 | 0.257 | 0.184 |
| CIS4 | 0.222 | 0.232 | 0.228 | 0.151 | 0.259 | 0.143 | 0.221 | 0.262 | 0.216 |
| CIS5 | 0.208 | 0.207 | 0.205 | 0.024 | 0.231 | 0.204 | 0.203 | 0.187 | 0.205 |
| CIS6 | 0.132 | 0.119 | 0.195 | 0.116 | 0.179 | 0.190 | 0.095 | 0.187 | 0.113 |

*Table I-C1. CFA-subsample polychoric correlation matrix, block 2 of 4 (AMS4–PEB6).*

| **Item** | **AMS4** | **AMS5** | **AMS6** | **PEB1** | **PEB2** | **PEB3** | **PEB4** | **PEB5** | **PEB6** |
| --- | --- | --- | --- | --- | --- | --- | --- | --- | --- |
| HWB1 | 0.217 | 0.177 | 0.237 | 0.150 | 0.203 | 0.198 | 0.154 | 0.050 | 0.257 |
| HWB2 | 0.192 | 0.249 | 0.264 | 0.262 | 0.292 | 0.274 | 0.283 | 0.204 | 0.348 |
| HWB3 | 0.280 | 0.317 | 0.261 | 0.224 | 0.242 | 0.164 | 0.266 | 0.196 | 0.284 |
| HWB4 | 0.207 | 0.236 | 0.138 | 0.222 | 0.230 | 0.129 | 0.199 | 0.161 | 0.259 |
| HWB5 | 0.194 | 0.209 | 0.206 | 0.172 | 0.173 | 0.163 | 0.266 | 0.133 | 0.251 |
| HWB6 | 0.199 | 0.209 | 0.246 | 0.237 | 0.270 | 0.174 | 0.285 | 0.118 | 0.218 |
| AMS1 | 0.434 | 0.442 | 0.434 | 0.226 | 0.319 | 0.231 | 0.321 | 0.226 | 0.218 |
| AMS2 | 0.471 | 0.469 | 0.485 | 0.288 | 0.306 | 0.218 | 0.316 | 0.239 | 0.246 |
| AMS3 | 0.407 | 0.447 | 0.471 | 0.174 | 0.293 | 0.243 | 0.294 | 0.207 | 0.167 |
| AMS4 | 1.000 | 0.466 | 0.504 | 0.236 | 0.255 | 0.217 | 0.183 | 0.207 | 0.167 |
| AMS5 | 0.466 | 1.000 | 0.436 | 0.207 | 0.251 | 0.209 | 0.226 | 0.159 | 0.143 |
| AMS6 | 0.504 | 0.436 | 1.000 | 0.133 | 0.253 | 0.164 | 0.237 | 0.231 | 0.201 |
| PEB1 | 0.236 | 0.207 | 0.133 | 1.000 | 0.466 | 0.390 | 0.447 | 0.401 | 0.487 |
| PEB2 | 0.255 | 0.251 | 0.253 | 0.466 | 1.000 | 0.433 | 0.471 | 0.479 | 0.436 |
| PEB3 | 0.217 | 0.209 | 0.164 | 0.390 | 0.433 | 1.000 | 0.424 | 0.428 | 0.447 |
| PEB4 | 0.183 | 0.226 | 0.237 | 0.447 | 0.471 | 0.424 | 1.000 | 0.411 | 0.466 |
| PEB5 | 0.207 | 0.159 | 0.231 | 0.401 | 0.479 | 0.428 | 0.411 | 1.000 | 0.316 |
| PEB6 | 0.167 | 0.143 | 0.201 | 0.487 | 0.436 | 0.447 | 0.466 | 0.316 | 1.000 |
| PID1 | 0.189 | 0.193 | 0.255 | 0.056 | 0.213 | 0.127 | 0.203 | 0.220 | 0.040 |
| PID2 | 0.181 | 0.199 | 0.222 | 0.174 | 0.221 | 0.142 | 0.176 | 0.162 | 0.107 |
| PID3 | 0.185 | 0.205 | 0.241 | 0.083 | 0.181 | 0.083 | 0.212 | 0.131 | 0.152 |
| PID4 | 0.167 | 0.241 | 0.168 | 0.156 | 0.228 | 0.111 | 0.245 | 0.171 | 0.166 |
| PID5 | 0.147 | 0.183 | 0.215 | 0.164 | 0.270 | 0.191 | 0.160 | 0.164 | 0.128 |
| PID6 | 0.150 | 0.221 | 0.189 | 0.221 | 0.290 | 0.278 | 0.276 | 0.225 | 0.239 |
| PCS1 | 0.186 | 0.335 | 0.246 | 0.137 | 0.157 | 0.151 | 0.076 | 0.098 | 0.092 |
| PCS2 | 0.237 | 0.348 | 0.292 | 0.226 | 0.252 | 0.141 | 0.186 | 0.212 | 0.235 |
| PCS3 | 0.237 | 0.312 | 0.230 | 0.117 | 0.228 | 0.174 | 0.148 | 0.081 | 0.167 |
| PCS4 | 0.255 | 0.303 | 0.295 | 0.157 | 0.233 | 0.242 | 0.168 | 0.092 | 0.143 |
| PCS5 | 0.270 | 0.339 | 0.288 | 0.216 | 0.151 | 0.172 | 0.174 | 0.137 | 0.111 |
| PCS6 | 0.207 | 0.301 | 0.253 | 0.133 | 0.227 | 0.134 | 0.152 | 0.072 | 0.206 |
| CIS1 | 0.197 | 0.209 | 0.200 | 0.271 | 0.258 | 0.164 | 0.206 | 0.146 | 0.137 |
| CIS2 | 0.205 | 0.188 | 0.182 | 0.181 | 0.316 | 0.276 | 0.264 | 0.183 | 0.151 |
| CIS3 | 0.186 | 0.228 | 0.233 | 0.173 | 0.181 | 0.139 | 0.256 | 0.175 | 0.212 |
| CIS4 | 0.237 | 0.230 | 0.159 | 0.255 | 0.205 | 0.277 | 0.276 | 0.186 | 0.190 |
| CIS5 | 0.235 | 0.173 | 0.204 | 0.170 | 0.231 | 0.206 | 0.235 | 0.169 | 0.187 |
| CIS6 | 0.237 | 0.186 | 0.231 | 0.172 | 0.135 | 0.175 | 0.162 | 0.209 | 0.118 |

*Table I-C1. CFA-subsample polychoric correlation matrix, block 3 of 4 (PID1–PCS3).*

| **Item** | **PID1** | **PID2** | **PID3** | **PID4** | **PID5** | **PID6** | **PCS1** | **PCS2** | **PCS3** |
| --- | --- | --- | --- | --- | --- | --- | --- | --- | --- |
| HWB1 | 0.070 | 0.029 | 0.151 | 0.172 | 0.152 | 0.124 | 0.128 | 0.247 | 0.177 |
| HWB2 | 0.120 | 0.146 | 0.143 | 0.166 | 0.197 | 0.147 | 0.122 | 0.213 | 0.243 |
| HWB3 | 0.148 | 0.170 | 0.185 | 0.242 | 0.147 | 0.149 | 0.188 | 0.274 | 0.200 |
| HWB4 | 0.127 | 0.134 | 0.259 | 0.227 | 0.141 | 0.278 | 0.092 | 0.071 | 0.119 |
| HWB5 | 0.129 | 0.140 | 0.138 | 0.241 | 0.156 | 0.176 | 0.101 | 0.137 | 0.140 |
| HWB6 | 0.161 | 0.081 | 0.169 | 0.179 | 0.185 | 0.199 | 0.147 | 0.247 | 0.240 |
| AMS1 | 0.210 | 0.261 | 0.221 | 0.299 | 0.196 | 0.210 | 0.182 | 0.263 | 0.251 |
| AMS2 | 0.156 | 0.214 | 0.149 | 0.193 | 0.197 | 0.196 | 0.203 | 0.288 | 0.213 |
| AMS3 | 0.103 | 0.119 | 0.142 | 0.187 | 0.200 | 0.148 | 0.206 | 0.280 | 0.249 |
| AMS4 | 0.189 | 0.181 | 0.185 | 0.167 | 0.147 | 0.150 | 0.186 | 0.237 | 0.237 |
| AMS5 | 0.193 | 0.199 | 0.205 | 0.241 | 0.183 | 0.221 | 0.335 | 0.348 | 0.312 |
| AMS6 | 0.255 | 0.222 | 0.241 | 0.168 | 0.215 | 0.189 | 0.246 | 0.292 | 0.230 |
| PEB1 | 0.056 | 0.174 | 0.083 | 0.156 | 0.164 | 0.221 | 0.137 | 0.226 | 0.117 |
| PEB2 | 0.213 | 0.221 | 0.181 | 0.228 | 0.270 | 0.290 | 0.157 | 0.252 | 0.228 |
| PEB3 | 0.127 | 0.142 | 0.083 | 0.111 | 0.191 | 0.278 | 0.151 | 0.141 | 0.174 |
| PEB4 | 0.203 | 0.176 | 0.212 | 0.245 | 0.160 | 0.276 | 0.076 | 0.186 | 0.148 |
| PEB5 | 0.220 | 0.162 | 0.131 | 0.171 | 0.164 | 0.225 | 0.098 | 0.212 | 0.081 |
| PEB6 | 0.040 | 0.107 | 0.152 | 0.166 | 0.128 | 0.239 | 0.092 | 0.235 | 0.167 |
| PID1 | 1.000 | 0.485 | 0.419 | 0.445 | 0.452 | 0.421 | 0.213 | 0.229 | 0.281 |
| PID2 | 0.485 | 1.000 | 0.383 | 0.479 | 0.502 | 0.459 | 0.157 | 0.085 | 0.166 |
| PID3 | 0.419 | 0.383 | 1.000 | 0.417 | 0.391 | 0.401 | 0.163 | 0.148 | 0.213 |
| PID4 | 0.445 | 0.479 | 0.417 | 1.000 | 0.467 | 0.477 | 0.166 | 0.176 | 0.255 |
| PID5 | 0.452 | 0.502 | 0.391 | 0.467 | 1.000 | 0.382 | 0.219 | 0.162 | 0.278 |
| PID6 | 0.421 | 0.459 | 0.401 | 0.477 | 0.382 | 1.000 | 0.191 | 0.140 | 0.207 |
| PCS1 | 0.213 | 0.157 | 0.163 | 0.166 | 0.219 | 0.191 | 1.000 | 0.456 | 0.449 |
| PCS2 | 0.229 | 0.085 | 0.148 | 0.176 | 0.162 | 0.140 | 0.456 | 1.000 | 0.464 |
| PCS3 | 0.281 | 0.166 | 0.213 | 0.255 | 0.278 | 0.207 | 0.449 | 0.464 | 1.000 |
| PCS4 | 0.214 | 0.138 | 0.197 | 0.248 | 0.250 | 0.164 | 0.505 | 0.535 | 0.465 |
| PCS5 | 0.218 | 0.141 | 0.136 | 0.212 | 0.243 | 0.209 | 0.413 | 0.448 | 0.491 |
| PCS6 | 0.195 | 0.114 | 0.200 | 0.153 | 0.140 | 0.167 | 0.452 | 0.457 | 0.417 |
| CIS1 | 0.206 | 0.272 | 0.181 | 0.259 | 0.311 | 0.256 | 0.176 | 0.164 | 0.202 |
| CIS2 | 0.177 | 0.214 | 0.154 | 0.239 | 0.284 | 0.234 | 0.183 | 0.163 | 0.215 |
| CIS3 | 0.241 | 0.248 | 0.264 | 0.248 | 0.275 | 0.207 | 0.250 | 0.148 | 0.183 |
| CIS4 | 0.229 | 0.240 | 0.184 | 0.208 | 0.155 | 0.230 | 0.227 | 0.221 | 0.235 |
| CIS5 | 0.158 | 0.194 | 0.156 | 0.211 | 0.265 | 0.205 | 0.153 | 0.172 | 0.184 |
| CIS6 | 0.229 | 0.161 | 0.181 | 0.211 | 0.251 | 0.190 | 0.175 | 0.153 | 0.175 |

*Table I-C1. CFA-subsample polychoric correlation matrix, block 4 of 4 (PCS4–CIS6).*

| **Item** | **PCS4** | **PCS5** | **PCS6** | **CIS1** | **CIS2** | **CIS3** | **CIS4** | **CIS5** | **CIS6** |
| --- | --- | --- | --- | --- | --- | --- | --- | --- | --- |
| HWB1 | 0.155 | 0.271 | 0.210 | 0.196 | 0.239 | 0.121 | 0.222 | 0.208 | 0.132 |
| HWB2 | 0.217 | 0.260 | 0.204 | 0.233 | 0.211 | 0.189 | 0.232 | 0.207 | 0.119 |
| HWB3 | 0.214 | 0.193 | 0.169 | 0.173 | 0.204 | 0.156 | 0.228 | 0.205 | 0.195 |
| HWB4 | 0.171 | 0.174 | 0.094 | 0.155 | 0.039 | 0.113 | 0.151 | 0.024 | 0.116 |
| HWB5 | 0.165 | 0.189 | 0.159 | 0.226 | 0.223 | 0.189 | 0.259 | 0.231 | 0.179 |
| HWB6 | 0.222 | 0.213 | 0.129 | 0.203 | 0.169 | 0.169 | 0.143 | 0.204 | 0.190 |
| AMS1 | 0.297 | 0.260 | 0.228 | 0.203 | 0.216 | 0.191 | 0.221 | 0.203 | 0.095 |
| AMS2 | 0.227 | 0.263 | 0.184 | 0.217 | 0.177 | 0.257 | 0.262 | 0.187 | 0.187 |
| AMS3 | 0.246 | 0.270 | 0.270 | 0.167 | 0.268 | 0.184 | 0.216 | 0.205 | 0.113 |
| AMS4 | 0.255 | 0.270 | 0.207 | 0.197 | 0.205 | 0.186 | 0.237 | 0.235 | 0.237 |
| AMS5 | 0.303 | 0.339 | 0.301 | 0.209 | 0.188 | 0.228 | 0.230 | 0.173 | 0.186 |
| AMS6 | 0.295 | 0.288 | 0.253 | 0.200 | 0.182 | 0.233 | 0.159 | 0.204 | 0.231 |
| PEB1 | 0.157 | 0.216 | 0.133 | 0.271 | 0.181 | 0.173 | 0.255 | 0.170 | 0.172 |
| PEB2 | 0.233 | 0.151 | 0.227 | 0.258 | 0.316 | 0.181 | 0.205 | 0.231 | 0.135 |
| PEB3 | 0.242 | 0.172 | 0.134 | 0.164 | 0.276 | 0.139 | 0.277 | 0.206 | 0.175 |
| PEB4 | 0.168 | 0.174 | 0.152 | 0.206 | 0.264 | 0.256 | 0.276 | 0.235 | 0.162 |
| PEB5 | 0.092 | 0.137 | 0.072 | 0.146 | 0.183 | 0.175 | 0.186 | 0.169 | 0.209 |
| PEB6 | 0.143 | 0.111 | 0.206 | 0.137 | 0.151 | 0.212 | 0.190 | 0.187 | 0.118 |
| PID1 | 0.214 | 0.218 | 0.195 | 0.206 | 0.177 | 0.241 | 0.229 | 0.158 | 0.229 |
| PID2 | 0.138 | 0.141 | 0.114 | 0.272 | 0.214 | 0.248 | 0.240 | 0.194 | 0.161 |
| PID3 | 0.197 | 0.136 | 0.200 | 0.181 | 0.154 | 0.264 | 0.184 | 0.156 | 0.181 |
| PID4 | 0.248 | 0.212 | 0.153 | 0.259 | 0.239 | 0.248 | 0.208 | 0.211 | 0.211 |
| PID5 | 0.250 | 0.243 | 0.140 | 0.311 | 0.284 | 0.275 | 0.155 | 0.265 | 0.251 |
| PID6 | 0.164 | 0.209 | 0.167 | 0.256 | 0.234 | 0.207 | 0.230 | 0.205 | 0.190 |
| PCS1 | 0.505 | 0.413 | 0.452 | 0.176 | 0.183 | 0.250 | 0.227 | 0.153 | 0.175 |
| PCS2 | 0.535 | 0.448 | 0.457 | 0.164 | 0.163 | 0.148 | 0.221 | 0.172 | 0.153 |
| PCS3 | 0.465 | 0.491 | 0.417 | 0.202 | 0.215 | 0.183 | 0.235 | 0.184 | 0.175 |
| PCS4 | 1.000 | 0.411 | 0.442 | 0.279 | 0.201 | 0.193 | 0.207 | 0.228 | 0.212 |
| PCS5 | 0.411 | 1.000 | 0.434 | 0.189 | 0.188 | 0.147 | 0.209 | 0.137 | 0.163 |
| PCS6 | 0.442 | 0.434 | 1.000 | 0.184 | 0.291 | 0.158 | 0.182 | 0.215 | 0.114 |
| CIS1 | 0.279 | 0.189 | 0.184 | 1.000 | 0.413 | 0.410 | 0.447 | 0.476 | 0.366 |
| CIS2 | 0.201 | 0.188 | 0.291 | 0.413 | 1.000 | 0.453 | 0.471 | 0.525 | 0.437 |
| CIS3 | 0.193 | 0.147 | 0.158 | 0.410 | 0.453 | 1.000 | 0.407 | 0.503 | 0.430 |
| CIS4 | 0.207 | 0.209 | 0.182 | 0.447 | 0.471 | 0.407 | 1.000 | 0.490 | 0.443 |
| CIS5 | 0.228 | 0.137 | 0.215 | 0.476 | 0.525 | 0.503 | 0.490 | 1.000 | 0.461 |
| CIS6 | 0.212 | 0.163 | 0.114 | 0.366 | 0.437 | 0.430 | 0.443 | 0.461 | 1.000 |

**Annex I-D. Complete fixed-split manifest**

The manifest below lists all 800 retained respondents in the exact order generated by set.seed(20260531). Records 1–400 were assigned to EFA and records 401–800 to CFA. The table is arranged four records across to limit page length; no records are omitted.

*Table I-D1. Complete split manifest for seed 20260531.*

| **Row** | **ID** | **Split** | **Row** | **ID** | **Split** | **Row** | **ID** | **Split** | **Row** | **ID** | **Split** |
| --- | --- | --- | --- | --- | --- | --- | --- | --- | --- | --- | --- |
| 506 | 506 | EFA | 364 | 364 | EFA | 739 | 739 | CFA | 355 | 355 | CFA |
| 147 | 147 | EFA | 621 | 621 | EFA | 241 | 241 | CFA | 16 | 16 | CFA |
| 459 | 459 | EFA | 493 | 493 | EFA | 670 | 670 | CFA | 348 | 348 | CFA |
| 66 | 66 | EFA | 541 | 541 | EFA | 563 | 563 | CFA | 576 | 576 | CFA |
| 575 | 575 | EFA | 772 | 772 | EFA | 512 | 512 | CFA | 703 | 703 | CFA |
| 437 | 437 | EFA | 319 | 319 | EFA | 317 | 317 | CFA | 398 | 398 | CFA |
| 628 | 628 | EFA | 602 | 602 | EFA | 547 | 547 | CFA | 750 | 750 | CFA |
| 409 | 409 | EFA | 795 | 795 | EFA | 478 | 478 | CFA | 707 | 707 | CFA |
| 169 | 169 | EFA | 231 | 231 | EFA | 581 | 581 | CFA | 381 | 381 | CFA |
| 337 | 337 | EFA | 327 | 327 | EFA | 378 | 378 | CFA | 727 | 727 | CFA |
| 126 | 126 | EFA | 680 | 680 | EFA | 60 | 60 | CFA | 41 | 41 | CFA |
| 138 | 138 | EFA | 101 | 101 | EFA | 200 | 200 | CFA | 350 | 350 | CFA |
| 58 | 58 | EFA | 78 | 78 | EFA | 749 | 749 | CFA | 33 | 33 | CFA |
| 72 | 72 | EFA | 347 | 347 | EFA | 360 | 360 | CFA | 193 | 193 | CFA |
| 722 | 722 | EFA | 420 | 420 | EFA | 636 | 636 | CFA | 189 | 189 | CFA |
| 217 | 217 | EFA | 260 | 260 | EFA | 773 | 773 | CFA | 272 | 272 | CFA |
| 537 | 537 | EFA | 98 | 98 | EFA | 311 | 311 | CFA | 45 | 45 | CFA |
| 295 | 295 | EFA | 48 | 48 | EFA | 580 | 580 | CFA | 222 | 222 | CFA |
| 416 | 416 | EFA | 482 | 482 | EFA | 654 | 654 | CFA | 552 | 552 | CFA |
| 399 | 399 | EFA | 160 | 160 | EFA | 1 | 1 | CFA | 723 | 723 | CFA |
| 698 | 698 | EFA | 326 | 326 | EFA | 233 | 233 | CFA | 603 | 603 | CFA |
| 436 | 436 | EFA | 513 | 513 | EFA | 733 | 733 | CFA | 517 | 517 | CFA |
| 46 | 46 | EFA | 203 | 203 | EFA | 711 | 711 | CFA | 518 | 518 | CFA |
| 204 | 204 | EFA | 508 | 508 | EFA | 672 | 672 | CFA | 232 | 232 | CFA |
| 282 | 282 | EFA | 366 | 366 | EFA | 579 | 579 | CFA | 529 | 529 | CFA |
| 600 | 600 | EFA | 43 | 43 | EFA | 597 | 597 | CFA | 285 | 285 | CFA |
| 469 | 469 | EFA | 310 | 310 | EFA | 551 | 551 | CFA | 403 | 403 | CFA |
| 534 | 534 | EFA | 243 | 243 | EFA | 146 | 146 | CFA | 582 | 582 | CFA |
| 595 | 595 | EFA | 754 | 754 | EFA | 8 | 8 | CFA | 15 | 15 | CFA |
| 485 | 485 | EFA | 566 | 566 | EFA | 83 | 83 | CFA | 77 | 77 | CFA |
| 700 | 700 | EFA | 514 | 514 | EFA | 176 | 176 | CFA | 384 | 384 | CFA |
| 453 | 453 | EFA | 264 | 264 | EFA | 128 | 128 | CFA | 338 | 338 | CFA |
| 104 | 104 | EFA | 11 | 11 | EFA | 67 | 67 | CFA | 356 | 356 | CFA |
| 502 | 502 | EFA | 367 | 367 | EFA | 297 | 297 | CFA | 140 | 140 | CFA |
| 743 | 743 | EFA | 298 | 298 | EFA | 573 | 573 | CFA | 421 | 421 | CFA |
| 339 | 339 | EFA | 354 | 354 | EFA | 457 | 457 | CFA | 17 | 17 | CFA |
| 294 | 294 | EFA | 650 | 650 | EFA | 587 | 587 | CFA | 674 | 674 | CFA |
| 682 | 682 | EFA | 640 | 640 | EFA | 328 | 328 | CFA | 179 | 179 | CFA |
| 687 | 687 | EFA | 158 | 158 | EFA | 290 | 290 | CFA | 751 | 751 | CFA |
| 85 | 85 | EFA | 194 | 194 | EFA | 207 | 207 | CFA | 331 | 331 | CFA |
| 131 | 131 | EFA | 521 | 521 | EFA | 633 | 633 | CFA | 143 | 143 | CFA |
| 156 | 156 | EFA | 626 | 626 | EFA | 692 | 692 | CFA | 212 | 212 | CFA |
| 18 | 18 | EFA | 274 | 274 | EFA | 405 | 405 | CFA | 10 | 10 | CFA |
| 452 | 452 | EFA | 454 | 454 | EFA | 94 | 94 | CFA | 764 | 764 | CFA |
| 306 | 306 | EFA | 76 | 76 | EFA | 192 | 192 | CFA | 667 | 667 | CFA |
| 135 | 135 | EFA | 681 | 681 | EFA | 709 | 709 | CFA | 247 | 247 | CFA |
| 308 | 308 | EFA | 277 | 277 | EFA | 275 | 275 | CFA | 734 | 734 | CFA |
| 286 | 286 | EFA | 413 | 413 | EFA | 523 | 523 | CFA | 278 | 278 | CFA |
| 108 | 108 | EFA | 679 | 679 | EFA | 477 | 477 | CFA | 625 | 625 | CFA |
| 197 | 197 | EFA | 394 | 394 | EFA | 753 | 753 | CFA | 263 | 263 | CFA |
| 253 | 253 | EFA | 390 | 390 | EFA | 656 | 656 | CFA | 686 | 686 | CFA |
| 760 | 760 | EFA | 165 | 165 | EFA | 741 | 741 | CFA | 495 | 495 | CFA |
| 798 | 798 | EFA | 458 | 458 | EFA | 569 | 569 | CFA | 505 | 505 | CFA |
| 466 | 466 | EFA | 719 | 719 | EFA | 735 | 735 | CFA | 657 | 657 | CFA |
| 382 | 382 | EFA | 481 | 481 | EFA | 149 | 149 | CFA | 9 | 9 | CFA |
| 377 | 377 | EFA | 729 | 729 | EFA | 614 | 614 | CFA | 380 | 380 | CFA |
| 182 | 182 | EFA | 386 | 386 | EFA | 720 | 720 | CFA | 630 | 630 | CFA |
| 460 | 460 | EFA | 769 | 769 | EFA | 226 | 226 | CFA | 706 | 706 | CFA |
| 659 | 659 | EFA | 111 | 111 | EFA | 752 | 752 | CFA | 393 | 393 | CFA |
| 609 | 609 | EFA | 96 | 96 | EFA | 591 | 591 | CFA | 464 | 464 | CFA |
| 47 | 47 | EFA | 153 | 153 | EFA | 441 | 441 | CFA | 95 | 95 | CFA |
| 117 | 117 | EFA | 80 | 80 | EFA | 185 | 185 | CFA | 119 | 119 | CFA |
| 799 | 799 | EFA | 362 | 362 | EFA | 548 | 548 | CFA | 655 | 655 | CFA |
| 357 | 357 | EFA | 238 | 238 | EFA | 542 | 542 | CFA | 391 | 391 | CFA |
| 216 | 216 | EFA | 562 | 562 | EFA | 170 | 170 | CFA | 730 | 730 | CFA |
| 736 | 736 | EFA | 82 | 82 | EFA | 500 | 500 | CFA | 56 | 56 | CFA |
| 725 | 725 | EFA | 676 | 676 | EFA | 49 | 49 | CFA | 610 | 610 | CFA |
| 23 | 23 | EFA | 761 | 761 | EFA | 315 | 315 | CFA | 550 | 550 | CFA |
| 262 | 262 | EFA | 410 | 410 | EFA | 293 | 293 | CFA | 791 | 791 | CFA |
| 213 | 213 | EFA | 715 | 715 | EFA | 642 | 642 | CFA | 184 | 184 | CFA |
| 365 | 365 | EFA | 309 | 309 | EFA | 509 | 509 | CFA | 161 | 161 | CFA |
| 320 | 320 | EFA | 522 | 522 | EFA | 227 | 227 | CFA | 395 | 395 | CFA |
| 303 | 303 | EFA | 623 | 623 | EFA | 671 | 671 | CFA | 2 | 2 | CFA |
| 574 | 574 | EFA | 530 | 530 | EFA | 584 | 584 | CFA | 528 | 528 | CFA |
| 175 | 175 | EFA | 590 | 590 | EFA | 712 | 712 | CFA | 738 | 738 | CFA |
| 100 | 100 | EFA | 118 | 118 | EFA | 323 | 323 | CFA | 443 | 443 | CFA |
| 789 | 789 | EFA | 777 | 777 | EFA | 163 | 163 | CFA | 691 | 691 | CFA |
| 744 | 744 | EFA | 479 | 479 | EFA | 324 | 324 | CFA | 450 | 450 | CFA |
| 29 | 29 | EFA | 218 | 218 | EFA | 186 | 186 | CFA | 125 | 125 | CFA |
| 299 | 299 | EFA | 340 | 340 | EFA | 585 | 585 | CFA | 472 | 472 | CFA |
| 532 | 532 | EFA | 250 | 250 | EFA | 448 | 448 | CFA | 346 | 346 | CFA |
| 490 | 490 | EFA | 742 | 742 | EFA | 539 | 539 | CFA | 702 | 702 | CFA |
| 658 | 658 | EFA | 248 | 248 | EFA | 774 | 774 | CFA | 59 | 59 | CFA |
| 62 | 62 | EFA | 538 | 538 | EFA | 663 | 663 | CFA | 651 | 651 | CFA |
| 473 | 473 | EFA | 402 | 402 | EFA | 148 | 148 | CFA | 419 | 419 | CFA |
| 26 | 26 | EFA | 35 | 35 | EFA | 201 | 201 | CFA | 84 | 84 | CFA |
| 307 | 307 | EFA | 57 | 57 | EFA | 214 | 214 | CFA | 255 | 255 | CFA |
| 499 | 499 | EFA | 102 | 102 | EFA | 168 | 168 | CFA | 583 | 583 | CFA |
| 462 | 462 | EFA | 634 | 634 | EFA | 177 | 177 | CFA | 767 | 767 | CFA |
| 683 | 683 | EFA | 666 | 666 | EFA | 787 | 787 | CFA | 422 | 422 | CFA |
| 335 | 335 | EFA | 792 | 792 | EFA | 463 | 463 | CFA | 690 | 690 | CFA |
| 333 | 333 | EFA | 223 | 223 | EFA | 252 | 252 | CFA | 498 | 498 | CFA |
| 244 | 244 | EFA | 370 | 370 | EFA | 520 | 520 | CFA | 256 | 256 | CFA |
| 195 | 195 | EFA | 782 | 782 | EFA | 288 | 288 | CFA | 133 | 133 | CFA |
| 38 | 38 | EFA | 371 | 371 | EFA | 527 | 527 | CFA | 358 | 358 | CFA |
| 677 | 677 | EFA | 343 | 343 | EFA | 644 | 644 | CFA | 785 | 785 | CFA |
| 205 | 205 | EFA | 480 | 480 | EFA | 336 | 336 | CFA | 228 | 228 | CFA |
| 332 | 332 | EFA | 771 | 771 | EFA | 376 | 376 | CFA | 219 | 219 | CFA |
| 605 | 605 | EFA | 174 | 174 | EFA | 745 | 745 | CFA | 404 | 404 | CFA |
| 69 | 69 | EFA | 800 | 800 | EFA | 535 | 535 | CFA | 124 | 124 | CFA |
| 449 | 449 | EFA | 24 | 24 | EFA | 270 | 270 | CFA | 296 | 296 | CFA |
| 716 | 716 | EFA | 259 | 259 | EFA | 613 | 613 | CFA | 39 | 39 | CFA |
| 150 | 150 | EFA | 271 | 271 | EFA | 385 | 385 | CFA | 40 | 40 | CFA |
| 107 | 107 | EFA | 694 | 694 | EFA | 379 | 379 | CFA | 375 | 375 | CFA |
| 714 | 714 | EFA | 465 | 465 | EFA | 65 | 65 | CFA | 434 | 434 | CFA |
| 721 | 721 | EFA | 417 | 417 | EFA | 53 | 53 | CFA | 497 | 497 | CFA |
| 240 | 240 | EFA | 7 | 7 | EFA | 796 | 796 | CFA | 245 | 245 | CFA |
| 221 | 221 | EFA | 251 | 251 | EFA | 468 | 468 | CFA | 740 | 740 | CFA |
| 28 | 28 | EFA | 578 | 578 | EFA | 543 | 543 | CFA | 758 | 758 | CFA |
| 284 | 284 | EFA | 25 | 25 | EFA | 544 | 544 | CFA | 525 | 525 | CFA |
| 588 | 588 | EFA | 424 | 424 | EFA | 756 | 756 | CFA | 717 | 717 | CFA |
| 19 | 19 | EFA | 236 | 236 | EFA | 411 | 411 | CFA | 235 | 235 | CFA |
| 312 | 312 | EFA | 592 | 592 | EFA | 73 | 73 | CFA | 790 | 790 | CFA |
| 549 | 549 | EFA | 704 | 704 | EFA | 116 | 116 | CFA | 75 | 75 | CFA |
| 400 | 400 | EFA | 515 | 515 | EFA | 476 | 476 | CFA | 589 | 589 | CFA |
| 302 | 302 | EFA | 188 | 188 | EFA | 423 | 423 | CFA | 775 | 775 | CFA |
| 675 | 675 | EFA | 396 | 396 | EFA | 224 | 224 | CFA | 467 | 467 | CFA |
| 536 | 536 | EFA | 471 | 471 | EFA | 446 | 446 | CFA | 599 | 599 | CFA |
| 34 | 34 | EFA | 408 | 408 | EFA | 190 | 190 | CFA | 112 | 112 | CFA |
| 234 | 234 | EFA | 693 | 693 | EFA | 615 | 615 | CFA | 130 | 130 | CFA |
| 157 | 157 | EFA | 115 | 115 | EFA | 765 | 765 | CFA | 456 | 456 | CFA |
| 142 | 142 | EFA | 70 | 70 | EFA | 438 | 438 | CFA | 594 | 594 | CFA |
| 54 | 54 | EFA | 560 | 560 | EFA | 647 | 647 | CFA | 620 | 620 | CFA |
| 351 | 351 | EFA | 162 | 162 | EFA | 353 | 353 | CFA | 12 | 12 | CFA |
| 369 | 369 | EFA | 763 | 763 | EFA | 571 | 571 | CFA | 414 | 414 | CFA |
| 90 | 90 | EFA | 577 | 577 | EFA | 42 | 42 | CFA | 279 | 279 | CFA |
| 428 | 428 | EFA | 427 | 427 | EFA | 141 | 141 | CFA | 737 | 737 | CFA |
| 44 | 44 | EFA | 555 | 555 | EFA | 747 | 747 | CFA | 776 | 776 | CFA |
| 239 | 239 | EFA | 455 | 455 | EFA | 74 | 74 | CFA | 397 | 397 | CFA |
| 374 | 374 | EFA | 254 | 254 | EFA | 173 | 173 | CFA | 407 | 407 | CFA |
| 418 | 418 | EFA | 123 | 123 | EFA | 778 | 778 | CFA | 361 | 361 | CFA |
| 645 | 645 | EFA | 89 | 89 | EFA | 202 | 202 | CFA | 32 | 32 | CFA |
| 4 | 4 | EFA | 352 | 352 | EFA | 230 | 230 | CFA | 770 | 770 | CFA |
| 151 | 151 | EFA | 486 | 486 | EFA | 215 | 215 | CFA | 564 | 564 | CFA |
| 699 | 699 | EFA | 206 | 206 | EFA | 489 | 489 | CFA | 37 | 37 | CFA |
| 258 | 258 | EFA | 511 | 511 | EFA | 113 | 113 | CFA | 695 | 695 | CFA |
| 329 | 329 | EFA | 291 | 291 | EFA | 368 | 368 | CFA | 718 | 718 | CFA |
| 598 | 598 | EFA | 724 | 724 | EFA | 281 | 281 | CFA | 557 | 557 | CFA |
| 301 | 301 | EFA | 267 | 267 | EFA | 755 | 755 | CFA | 652 | 652 | CFA |
| 697 | 697 | EFA | 662 | 662 | EFA | 321 | 321 | CFA | 273 | 273 | CFA |
| 669 | 669 | EFA | 601 | 601 | EFA | 106 | 106 | CFA | 668 | 668 | CFA |
| 21 | 21 | EFA | 129 | 129 | EFA | 611 | 611 | CFA | 526 | 526 | CFA |
| 199 | 199 | EFA | 637 | 637 | EFA | 593 | 593 | CFA | 678 | 678 | CFA |
| 27 | 27 | EFA | 607 | 607 | EFA | 120 | 120 | CFA | 389 | 389 | CFA |
| 265 | 265 | EFA | 225 | 225 | EFA | 632 | 632 | CFA | 289 | 289 | CFA |
| 793 | 793 | EFA | 568 | 568 | EFA | 664 | 664 | CFA | 63 | 63 | CFA |
| 145 | 145 | EFA | 5 | 5 | EFA | 3 | 3 | CFA | 606 | 606 | CFA |
| 689 | 689 | EFA | 392 | 392 | EFA | 387 | 387 | CFA | 491 | 491 | CFA |
| 266 | 266 | EFA | 570 | 570 | EFA | 134 | 134 | CFA | 122 | 122 | CFA |
| 768 | 768 | EFA | 429 | 429 | EFA | 432 | 432 | CFA | 608 | 608 | CFA |
| 322 | 322 | EFA | 20 | 20 | EFA | 445 | 445 | CFA | 406 | 406 | CFA |
| 559 | 559 | EFA | 372 | 372 | EFA | 732 | 732 | CFA | 166 | 166 | CFA |
| 127 | 127 | EFA | 688 | 688 | EFA | 237 | 237 | CFA | 401 | 401 | CFA |
| 191 | 191 | EFA | 86 | 86 | EFA | 349 | 349 | CFA | 334 | 334 | CFA |
| 249 | 249 | EFA | 211 | 211 | EFA | 71 | 71 | CFA | 167 | 167 | CFA |
| 617 | 617 | EFA | 781 | 781 | EFA | 242 | 242 | CFA | 196 | 196 | CFA |
| 561 | 561 | EFA | 373 | 373 | EFA | 797 | 797 | CFA | 567 | 567 | CFA |
| 757 | 757 | EFA | 431 | 431 | EFA | 726 | 726 | CFA | 660 | 660 | CFA |
| 501 | 501 | EFA | 415 | 415 | EFA | 519 | 519 | CFA | 220 | 220 | CFA |
| 136 | 136 | EFA | 383 | 383 | EFA | 93 | 93 | CFA | 646 | 646 | CFA |
| 412 | 412 | EFA | 627 | 627 | EFA | 701 | 701 | CFA | 388 | 388 | CFA |
| 624 | 624 | EFA | 325 | 325 | EFA | 510 | 510 | CFA | 341 | 341 | CFA |
| 314 | 314 | EFA | 6 | 6 | EFA | 159 | 159 | CFA | 30 | 30 | CFA |
| 524 | 524 | EFA | 794 | 794 | EFA | 631 | 631 | CFA | 51 | 51 | CFA |
| 276 | 276 | EFA | 229 | 229 | EFA | 665 | 665 | CFA | 447 | 447 | CFA |
| 88 | 88 | EFA | 708 | 708 | EFA | 639 | 639 | CFA | 31 | 31 | CFA |
| 144 | 144 | EFA | 178 | 178 | EFA | 121 | 121 | CFA | 648 | 648 | CFA |
| 52 | 52 | EFA | 461 | 461 | EFA | 36 | 36 | CFA | 152 | 152 | CFA |
| 470 | 470 | EFA | 342 | 342 | EFA | 635 | 635 | CFA | 540 | 540 | CFA |
| 164 | 164 | EFA | 713 | 713 | EFA | 488 | 488 | CFA | 79 | 79 | CFA |
| 492 | 492 | EFA | 483 | 483 | EFA | 181 | 181 | CFA | 684 | 684 | CFA |
| 474 | 474 | EFA | 546 | 546 | EFA | 180 | 180 | CFA | 629 | 629 | CFA |
| 779 | 779 | EFA | 172 | 172 | EFA | 110 | 110 | CFA | 300 | 300 | CFA |
| 545 | 545 | EFA | 257 | 257 | EFA | 363 | 363 | CFA | 612 | 612 | CFA |
| 558 | 558 | EFA | 210 | 210 | EFA | 440 | 440 | CFA | 451 | 451 | CFA |
| 304 | 304 | EFA | 728 | 728 | EFA | 287 | 287 | CFA | 305 | 305 | CFA |
| 183 | 183 | EFA | 696 | 696 | EFA | 616 | 616 | CFA | 638 | 638 | CFA |
| 516 | 516 | EFA | 504 | 504 | EFA | 345 | 345 | CFA | 137 | 137 | CFA |
| 685 | 685 | EFA | 780 | 780 | EFA | 444 | 444 | CFA | 430 | 430 | CFA |
| 13 | 13 | EFA | 746 | 746 | EFA | 61 | 61 | CFA | 710 | 710 | CFA |
| 198 | 198 | EFA | 487 | 487 | EFA | 109 | 109 | CFA | 268 | 268 | CFA |
| 55 | 55 | EFA | 292 | 292 | EFA | 359 | 359 | CFA | 50 | 50 | CFA |
| 280 | 280 | EFA | 435 | 435 | EFA | 673 | 673 | CFA | 604 | 604 | CFA |
| 649 | 649 | EFA | 22 | 22 | EFA | 759 | 759 | CFA | 643 | 643 | CFA |
| 586 | 586 | EFA | 475 | 475 | EFA | 619 | 619 | CFA | 269 | 269 | CFA |
| 705 | 705 | EFA | 155 | 155 | EFA | 103 | 103 | CFA | 442 | 442 | CFA |
| 784 | 784 | EFA | 618 | 618 | EFA | 484 | 484 | CFA | 783 | 783 | CFA |
| 661 | 661 | EFA | 81 | 81 | EFA | 653 | 653 | CFA | 14 | 14 | CFA |
| 503 | 503 | EFA | 762 | 762 | EFA | 208 | 208 | CFA | 139 | 139 | CFA |
| 330 | 330 | EFA | 68 | 68 | EFA | 261 | 261 | CFA | 87 | 87 | CFA |
| 496 | 496 | EFA | 439 | 439 | EFA | 425 | 425 | CFA | 748 | 748 | CFA |
| 556 | 556 | EFA | 97 | 97 | EFA | 313 | 313 | CFA | 554 | 554 | CFA |
| 209 | 209 | EFA | 316 | 316 | EFA | 246 | 246 | CFA | 92 | 92 | CFA |
| 171 | 171 | EFA | 426 | 426 | EFA | 507 | 507 | CFA | 531 | 531 | CFA |
| 154 | 154 | EFA | 114 | 114 | EFA | 99 | 99 | CFA | 622 | 622 | CFA |
| 572 | 572 | EFA | 187 | 187 | EFA | 132 | 132 | CFA | 91 | 91 | CFA |
| 64 | 64 | EFA | 105 | 105 | EFA | 731 | 731 | CFA | 641 | 641 | CFA |
| 766 | 766 | EFA | 786 | 786 | EFA | 533 | 533 | CFA | 788 | 788 | CFA |
| 318 | 318 | EFA | 494 | 494 | EFA | 283 | 283 | CFA | 553 | 553 | CFA |
| 344 | 344 | EFA | 596 | 596 | EFA | 565 | 565 | CFA | 433 | 433 | CFA |

**Annex I-E. Measurement-invariance code**

This code is the version used to reproduce Table I-13. It preserves the manuscript's grouping rules and applies threshold constraints before threshold-plus-loading constraints.

if (is.na(parallel::detectCores())) {

unlockBinding("detectCores", asNamespace("parallel"))

assign("detectCores", function(logical = TRUE) 1L, envir = asNamespace("parallel"))

lockBinding("detectCores", asNamespace("parallel"))

}

suppressPackageStartupMessages(library(lavaan))

options(lavaan.ncpus = 1)

file_arg <- grep("^--file=", commandArgs(trailingOnly = FALSE), value = TRUE)

script_path <- if (length(file_arg)) sub("^--file=", "", file_arg[1]) else "measurement_invariance_verification.R"

script_dir <- dirname(normalizePath(script_path, mustWork = FALSE))

data_path <- file.path(script_dir, "measurement_invariance_data.csv")

if (!file.exists(data_path)) {

stop("measurement_invariance_data.csv must be stored beside this script.")

}

out <- script_dir

dat <- read.csv(data_path, check.names = FALSE)

items <- c(

paste0("HWB", 1:6), paste0("AMS", 1:6), paste0("PEB", 1:6),

paste0("PID", 1:6), paste0("PCS", 1:6), paste0("CIS", 1:6)

)

model6 <- '

HWB =~ HWB1 + HWB2 + HWB3 + HWB4 + HWB5 + HWB6

AMS =~ AMS1 + AMS2 + AMS3 + AMS4 + AMS5 + AMS6

PEB =~ PEB1 + PEB2 + PEB3 + PEB4 + PEB5 + PEB6

PID =~ PID1 + PID2 + PID3 + PID4 + PID5 + PID6

PCS =~ PCS1 + PCS2 + PCS3 + PCS4 + PCS5 + PCS6

CIS =~ CIS1 + CIS2 + CIS3 + CIS4 + CIS5 + CIS6

'

fit_names <- c(

"chisq.scaled", "df.scaled", "pvalue.scaled",

"cfi.scaled", "tli.scaled", "rmsea.scaled", "srmr",

"cfi.robust", "tli.robust", "rmsea.robust"

)

warning_log <- character()

run_group <- function(group_var, label, level_order) {

local <- dat[!is.na(dat[[group_var]]) & dat[[group_var]] != "", ]

local[[group_var]] <- factor(local[[group_var]], levels = level_order)

capture_fit <- function(equal = NULL) {

withCallingHandlers(

cfa(

model6, data = local, group = group_var, ordered = items,

estimator = "WLSMV", parameterization = "delta", std.lv = TRUE,

missing = "listwise", group.equal = equal, parallel = "no"

),

warning = function(w) {

warning_log <<- c(warning_log, paste(label, conditionMessage(w), sep = ": "))

invokeRestart("muffleWarning")

}

)

}

configural <- capture_fit()

thresholds <- capture_fit("thresholds")

thresholds_loadings <- capture_fit(c("thresholds", "loadings"))

fits <- list(

Configural = configural,

Threshold = thresholds,

`Threshold + loading` = thresholds_loadings

)

fit_rows <- do.call(rbind, lapply(names(fits), function(step) {

values <- fitMeasures(fits[[step]], fit_names)

data.frame(

comparison = label,

group_variable = group_var,

model = step,

n = nrow(local),

as.list(values),

converged = lavInspect(fits[[step]], "converged"),

iterations = lavInspect(fits[[step]], "iterations"),

check.names = FALSE

)

}))

rownames(fit_rows) <- NULL

fit_rows$delta_cfi <- c(NA, diff(fit_rows$cfi.scaled))

fit_rows$delta_rmsea <- c(NA, diff(fit_rows$rmsea.scaled))

fit_rows$delta_srmr <- c(NA, diff(fit_rows$srmr))

counts <- as.data.frame(table(local[[group_var]]), stringsAsFactors = FALSE)

names(counts) <- c("group", "n")

counts$comparison <- label

counts$group_variable <- group_var

list(fit = fit_rows, counts = counts)

}

gender <- run_group("gender", "Gender", c("Male", "Female"))

team <- run_group("team_coaching_role", "School team coaching role", c("No", "Yes"))

stage <- run_group("school_stage", "School stage", c("Primary", "Secondary"))

all_fit <- rbind(gender$fit, team$fit, stage$fit)

all_counts <- rbind(gender$counts, team$counts, stage$counts)

write.csv(all_fit, file.path(out, "measurement_invariance_corrected_results.csv"), row.names = FALSE)

write.csv(all_counts, file.path(out, "measurement_invariance_group_counts.csv"), row.names = FALSE)

writeLines(

if (length(warning_log)) unique(warning_log) else "No estimation warnings.",

file.path(out, "measurement_invariance_warnings.txt")

)

writeLines(c(

"Estimator: WLSMV; ordered indicators; delta parameterization; std.lv = TRUE.",

"Model sequence: configural; equal thresholds; equal thresholds and loadings.",

"Reference groups: Male; No school team coaching role; Primary school.",

"Configural identification: item intercepts fixed to 0, item-response scaling factors fixed to 1, latent means fixed to 0, and latent variances fixed to 1 in each group.",

"Threshold model: all four thresholds per item constrained equal; item intercepts and item-response scaling factors freed in the comparison group by lavaan's categorical multiple-group identification rules.",

"Threshold-plus-loading model: thresholds and loadings constrained equal; reference-group latent variances fixed to 1 and comparison-group latent variances estimated.",

"Latent means remained fixed to 0; no intercept, residual-variance, latent-mean, or latent-covariance equality constraints were tested."

), file.path(out, "measurement_invariance_identification_settings.txt"))

sink(file.path(out, "measurement_invariance_sessionInfo.txt"))

sessionInfo()

sink()

print(all_fit[, c(

"comparison", "model", "n", "chisq.scaled", "df.scaled",

"cfi.scaled", "tli.scaled", "rmsea.scaled", "srmr",

"delta_cfi", "delta_rmsea", "delta_srmr", "converged", "iterations"

)])

print(all_counts)

print(if (length(warning_log)) unique(warning_log) else "No estimation warnings.")
